# Bacteria‐Targeted Single‐Atom Nanozyme With Photothermal‐Augmented Multi‐Enzymatic Cascade and NO Delivery for Enhanced Infected Wound Healing

**DOI:** 10.1002/advs.202509621

**Published:** 2025-08-14

**Authors:** Junyang Chen, Qing Chen, Xudong Qin, Haixia Yang, Xin Wang, Jianliang Zhou, Ying‐Wei Yang, Jian Tian

**Affiliations:** ^1^ Department of Cardiovascular Surgery Zhongnan Hospital of Wuhan University School of Pharmaceutical Sciences Wuhan University Wuhan 430071 P. R. China; ^2^ College of Chemistry Jilin University 2699 Qianjin Street Changchun 130012 P. R. China; ^3^ Department of Radiation Oncology China‐Japan Union Hospital of Jilin University Changchun 130033 P. R. China

**Keywords:** antibacterial materials, biomaterials, nanoplatform, nanozymes, photothermal therapy, wound healing

## Abstract

Infected diabetic wound management confronts significant challenges, including bacterial resistance, oxidative stress, and impaired vascular repair, resulting in substantial unmet clinical needs. To address these issues, a multifunctional therapeutic nanoplatform, mCu‐SAE@BNN6@PEG‐Van (CBPV), is developed by sequentially functionalizing mesoporous copper single‐atom nanozymes (mCu‐SAE) loaded with the nitric oxide (NO) donor BNN6 and vancomycin‐conjugated polyethylene glycol (PEG‐Van). CBPV integrates three synergistic therapeutic modalities: 1) pathogen‐specific targeting via Van‐mediated bacterial recognition; 2) NIR‐II photothermally enhanced catalytic therapy via Cu‐N_3_ centers in mCu‐SAE, generating reactive oxygen species; 3) photoactivated NO release from BNN6, enabling peroxynitrite (ONOO^−^) formation through radical coupling. Irradiation of CBPV with a 1064‐nm laser simultaneously enables deep‐tissue photothermal activation, thermally boosted chemodynamic activity, and controlled NO liberation. In vitro and in vivo studies demonstrate that CBPV exhibits remarkable antibiofilm activity and antibacterial efficacy against methicillin‐resistant Staphylococcus aureus (99.6% inactivation) while promoting angiogenesis through NO‐mediated endothelial cell activation. Both epidermal wound and subcutaneous cyst models show accelerated healing with enhanced collagen deposition and neovascularization. By integrating bacterial targeting with NIR‐II‐responsive therapeutic cascades, this work establishes a spatiotemporally controlled therapeutic paradigm that simultaneously addresses infection control and tissue regeneration in chronic wounds, offering a promising translational strategy for managing complex diabetic wounds.

## Graphical abstract

A single‐atom nanozyme‐based multifunctional nanoplatform synergizes bacterial targeting with a NIR‐II responsive therapeutic cascade for effective diabetic wound management. This nanoplatform enhances biofilm destruction through photothermal‐augmented biocatalysis while simultaneously activating endothelial cells by delivering nitric oxide to promote tissue repair. It addresses two critical functions: eliminating drug‐resistant bacteria and facilitating angiogenesis, enabling precise treatment of chronic infectious wounds.

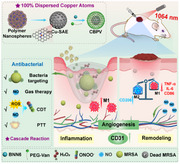

## 1 Introduction

Diabetes mellitus, a prevalent endocrine disorder, exhibits a steadily rising incidence annually.^[^ ^1^ ^]^ Diabetes mellitus can precipitate a myriad of complications, with diabetic foot ulcers (DFUs) ranking among the most severe.^[^ ^2^ ^]^ The high glucose levels in and around diabetic wounds can promote bacterial proliferation and impair the bactericidal efficacy of the immune system, thereby impeding wound healing.^[^ ^3^ ^]^ While conventional antimicrobial treatments brought significant advancements in wound management, their widespread use has resulted in the development of drug‐resistant bacteria, raising concerns about the decreasing number of effective treatment options available.^[^ ^4^ ^]^ Furthermore, even advanced biological therapies, such as growth factors, have not consistently yielded positive outcomes, with complete wound closure being achieved in fewer than 50% of cases, possibly due to protease‐rich wound beds degrading therapeutic proteins.^[^ ^2^, ^5^ ^]^ This therapeutic dilemma underscores the urgent need for next‐generation treatment strategies to effectively target resistant pathogens, enhance cellular healing, and remodel hostile wound microenvironments.

Enzyme‐catalyzed therapy has garnered considerable attention for the treatment of various diseases.^[^ ^6^ ^]^ Natural enzymes in living organisms, such as peroxidase (POD) and oxidase (OXD), exhibit antimicrobial effects by catalyzing the production of hydroxyl radicals (•OH), superoxide anions (•O_2_^−^), and other reactive oxygen species (ROS) from hydrogen peroxide (H_2_O_2_).^[^ ^7^ ^]^ However, the high sensitivity of natural enzymes to environmental factors like temperature and pH severely limits their clinical application.^[^ ^8^ ^]^ Recently, nanozymes, nanomaterials that emulate the catalytic activities of natural enzymes, have emerged as promising candidates for natural enzymes due to their low cost, high stability, and scalable preparation.^[^ ^9^ ^]^ Since the groundbreaking report on Fe_3_O_4_ nanoparticles (NPs) exhibiting POD‐like activity in 2007, nanozymes have garnered considerable interest across various applications, including immunoassays, antibacterial treatments, and biosensors.^[^ ^10^ ^]^ Despite these significant advancements, the metal atoms in most nanozymes remain underutilized, leading to generally suboptimal catalytic activity.^[^ ^11^ ^]^ To address this issue, the reactive sites of NPs have been reduced to the atomic scale, thereby increasing the active center's density and enhancing nanozyme catalytic activity.^[^ ^12^ ^]^ These single‐atom nanozymes (SAzymes) possess more effectively mimic natural enzyme active centers, yielding a similar catalytic mechanism and significantly improved performance.^[^ ^13^ ^]^ A notable example, Xu and co‐workers^[^ ^14^ ^]^ devised SAzymes equipped with Zn‐N_4_ catalytic cores, demonstrating extraordinary POD‐like action and in vitro antibacterial potency, notably achieving a 99.9% inhibition rate against Pseudomonas aeruginosa. Fan et al.^[^ ^3^ ^]^ coordinated copper atoms with one oxygen atom and two nitrogen atoms, resulting in an active center that exhibited superior POD activity compared to natural horseradish peroxidase (HRP). This active center demonstrated a significant antibacterial effect against both Gram‐negative and Gram‐positive bacteria.

Additionally, according to the Arrhenius equation, temperature significantly influences enzymatic activity. As the temperature increases, the ability of nanozymes to generate ROS improves considerably.^[^ ^15^ ^]^ However, directly heating NPs in vivo is infeasible, particularly for subcutaneous infections.^[^ ^16^ ^]^ This challenge has driven interest in photothermal conversion strategies using near‐infrared (NIR)‐responsive nanomaterials, where NIR‐I (700‐900 nm) and NIR‐II (1000–1700 nm) irradiation enables spatiotemporal thermal regulation through nanoparticle‐mediated photothermal therapy (PTT).^[^ ^17^ ^]^ Beyond enhancing enzymatic bactericidal activity, PTT offers multifunctional advantages such as localized hyperthermia, non‐invasive operation, and minimized antimicrobial resistance development.^[^ ^18^ ^]^ While conventional 808‐nm lasers predominate biomedical applications, their limited tissue penetration and low skin tolerance limit efficacy in deep infections while risking collateral damage.^[^ ^19^ ^]^ Emerging NIR‐II systems (1000–1700 nm) demonstrate superior clinical potential through enhanced penetration depth and elevated skin tolerance, achieved via reduced photon scattering and tissue absorption coefficients.^[^ ^15^, ^20^ ^]^ Nevertheless, current single‐mode SAzyme platforms face critical limitations – prolonged irradiation induces cytotoxic temperature spikes and excessive ROS production, causing significant off‐target tissue damage.^[^ ^21^ ^]^ This highlights the urgent need to develop intelligent nanoplatforms that integrate multimodal bactericidal mechanisms with precise targeting capabilities to achieve infection‐selective therapy while preserving host microenvironment homeostasis.

In addition to PTT and chemodynamic therapy (CDT), recent advancements in biomedical gas therapy have elevated nitric oxide (NO) to the forefront of innovation in diabetic wound management.^[^ ^22^ ^]^ As a pleiotropic signaling molecule, NO exerts broad‐spectrum antimicrobial activity through dual mechanisms: direct bacterial eradication via lipid peroxidation cascades and enhancement of reactive nitrogen species (RNS) through ROS‐dependent peroxynitrite (ONOO^−^) formation.^[^ ^23^ ^]^ Beyond its antimicrobial efficacy, NO can also promote angiogenesis and collagen deposition by augmenting fibroblast production.^[^ ^24^ ^]^ Nevertheless, the clinical translation of NO‐based therapies remains constrained by its short biological half‐life and the inherent challenge of achieving spatiotemporal control over gaseous release profiles.^[^ ^25^ ^]^ To address these limitations, current research has focused on developing stimulus‐responsive delivery platforms that integrate molecular targeting with controlled NO release. A lesion‐specific NO‐controlled release system can enhance therapeutic efficacy while reducing damage to normal tissues.^[^ ^26^ ^]^ Molecules targeting bacterial surface receptors, including antibiotics,^[^ ^27^ ^]^ recognition peptides,^[^ ^28^ ^]^ and antimicrobial peptides,^[^ ^29^ ^]^ are widely employed as antimicrobial therapy. As a prototypical antibiotic, vancomycin (Van) exhibits a high affinity for the (D)‐Ala‐(D)‐ Ala peptide residue present in the cell walls of both Gram‐positive and Gram‐negative bacteria.^[^ ^27^ ^]^ Therefore, we hypothesize that integrating Van with thermally activated NO donors may represent an effective strategy. This approach enables the precise release of NO in the wound area during photothermal activation, thereby enhancing the efficacy of gas therapy while mitigating damage to healthy tissues.^[^ ^30^ ^]^

**Scheme 1:**
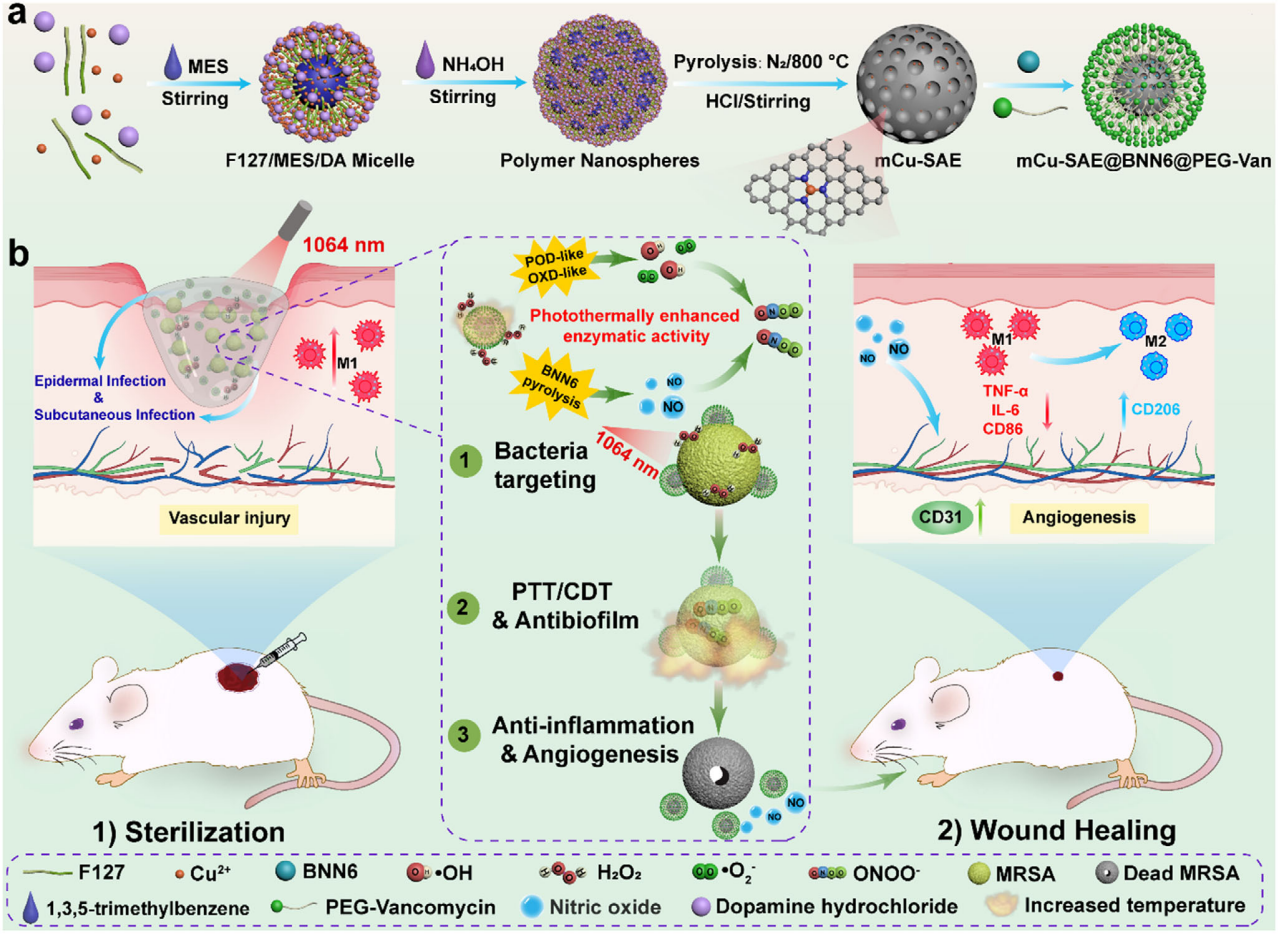
A schematic diagram illustrating the treatment of diabetic epidermal wounds and subcutaneous cysts using CBPV. a) The synthesis process of CBPV. b) Combined PTT, CDT, and NO therapy using CBPV and NIR‐II laser for epidermal and subcutaneous infections.

Building upon these mechanistic insights, we developed a bacteria‐targeted nanoarchitecture (mCu‐SAE@BNN6@PEG‐Van, designated as CBPV) that orchestrates NO/CDT/PTT multimodal therapy through spatiotemporally controlled reactive species generation, thereby mitigating the biosafety concerns associated with single‐modality therapeutic approaches (**Scheme** **1**). CBPV is fabricated by sequentially functionalizing mesoporous copper single‐atom nanozymes (mCu‐SAE) loaded with the NO donor N,N″‐di‐sec‐butyl N,N″‐dinitroso‐1,4‐phenylenediamine (BNN6) and modified with Van‐conjugated polyethylene glycol (PEG‐Van) for bacterial targeting. The single‐atom Cu nanozyme we engineered features an unconventional Cu‐N_3_ coordination structure, distinct from the typical Cu‐N_4_ configuration. Owing to its unique coordination geometry, the Cu‐N_3_ center exhibits enhanced interactions with H_2_O_2_, achieving the unhindered dissociation of H_2_O_2_ in the process of producing •OH‐a phenomenon absent in Cu‐N_4_ systems.^[^ ^31^ ^]^ Consequently, CBPV displays remarkable catalytic activity, mimicking both POD‐like and OXD‐like enzymes. This dual enzymatic behavior drives the robust generation of •OH and •O_2_^−^, resulting in potent antibacterial efficacy within a weakly acidic H_2_O_2_‐enriched microenvironment. Surface‐anchored Van ligands provide precise bacterial targeting, significantly enhancing accumulation at infection sites compared to non‐targeted counterparts while maintaining tissue integrity. The polydopamine‐derived carbon matrices enable superior NIR‐II (1064 nm) photothermal conversion (η = 43.9%), synergistically amplifying CDT efficacy through thermal acceleration of enzyme‐like activities while triggering temperature‐responsive BNN6 decomposition for NO liberation. Notably, the dynamic NO release from CBPV displays biphasic therapeutic logic: 1) In the initial bactericidal phase, NO reacts with primary radicals (•OH/•O_2_^−^) to yield bactericidal ONOO^−^, establishing a self‐reinforcing oxidative cascade. 2) During the subsequent healing phase, a low concentration of NO released over time stimulates fibroblast migration (a 4.8‐fold increase compared to control) and enhances collagen maturation. The NIR‐II activation modality further enables deep‐tissue penetration, effectively eradicating methicillin‐resistant *Staphylococcus aureus* (MRSA) biofilms in subcutaneous infection models. This spatiotemporal orchestration of reactive species chemistry with bacterial precision targeting establishes an innovative platform for synergistic antimicrobial therapy and tissue regeneration.

## 2 Results and Discussion

### 2.1 Design and Characterization of CBPV

By integrating single‐atom copper nanozymes (mCu‐SAE) with the targeting ligand (Van) and NO donors (BNN6), the designed CBPV nanosystem aims to combine bacterial targeting with chemodynamic therapy, photothermal capabilities, and NO delivery functions, achieving spatiotemporally controlled cascade catalytic characteristics. The synthetic route of mCu‐SAE and CBPV is depicted in Scheme 1a. In summary, dopamine hydrochloride (DA) serves as the nitrogen and carbon source for the synthesis of mCu‐SAE, while copper(II) acetylacetonate provides the copper source. The reactants undergo polymerization and self‐assembly in a water/ethanol reaction system, followed by high‐temperature carbonization and hydrochloric acid etching to yield mCu‐SAE. During the synthesis, copper ions can chelate with the amino, hydroxyl, and phenolic hydroxyl groups on DA, which helps anchor them to the support. Pluronic F‐127 (F127), assisted by the stabilizer 1,3,5‐trimethylbenzene (MES), facilitates the formation of mesopores. Upon adding ammonia, DA is oxidized to form quinone, leading to polymerization and the formation of Cu/PDA/F127 polydopamine nanospheres.^[^ ^32^ ^]^ Subsequently, these nanospheres are subjected to high‐temperature calcination in a tubular furnace, resulting in mesoporous carbon nanospheres after the thermal decomposition of F127 and MES. Through structural optimization, the synthesized mCu‐SAE exhibits significant differences from previously reported single‐atom manganese and iron nanozymes.^[^ ^13^, ^33^ ^]^ Specifically, the single‐atom copper in mCu‐SAE has a low‐coordination configuration, coordinating with three nitrogen atoms instead of four. This CuN_3_‐SAzyme, with coordinatively unsaturated active copper sites, facilitates a strong interaction between the copper single atom and H_2_O_2_ molecules, enabling the unimpeded dissociation of H_2_O_2_. In contrast, this interaction is significantly weaker in the conventional CuN_4_‐SAzyme, which hinders H_2_O_2_ from adsorbing at the reaction site.^[^ ^3^, ^31^ ^]^ Consequently, adjusting the coordination environment of the single‐atom copper substantially enhances the enzyme‐like activity of the nanozymes. N‐doped mesoporous carbon nanospheres (NMCNPs), without copper metal, were prepared using the same method, excluding the addition of copper salt.

**Figure 1:**
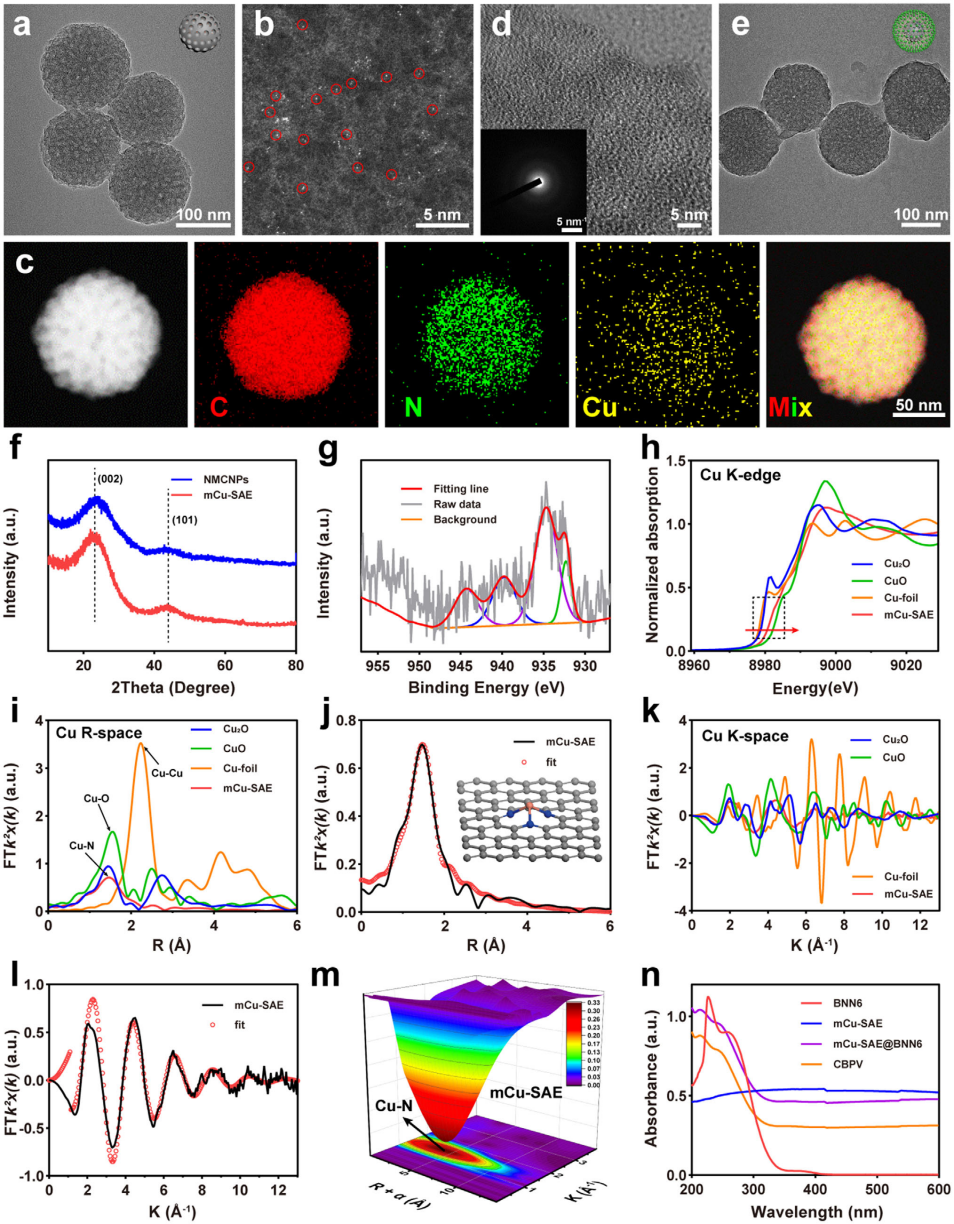
Characterization of mCu‐SAE and CBPV. TEM images of a) mCu‐SAE and e) CBPV. Scale bar = 100 nm. b) HAADF‐STEM image of mCu‐SAE. Scale bar = 5 nm. c) Element mapping images of mCu‐SAE. Scale bar = 50 nm. d) HRTEM image of mCu‐SAE. Scale bar = 5 nm. Illustration: SAED image of mCu‐SAE. Scale bar = 5 nm^−1^. f) XRD patterns of mCu‐SAE and NMCNPs. g) The high‐resolution Cu 2p_3/2_ XPS spectra of mCu‐SAE. h, i) XANES and EXAFS spectra at Cu K‐edge of mCu‐SAE. j) FT‐EXAFS fitting curves at R space of Cu K‐edge for mCu‐SAE. k) K^2^χ(k) space spectra fitting curve of Cu foil, CuO, Cu_2_O, and mCu‐SAE. l) K^2^χ(k) space spectra fitting curve of mCu‐SAE. m) WT‐EXAFS plots of mCu‐SAE. n) UV–vis spectra of BNN6, mCu‐SAE, mCu‐SAE@BNN6 and CBPV.

We then systematically studied the chemical and physical structures of mCu‐SAE using various techniques, including scanning electron microscopy (SEM), transmission electron microscopy (TEM), X‐ray powder diffraction (XRD), X‐ray photoelectron spectroscopy (XPS), X‐ray absorption spectroscopy (XAS), and Raman spectroscopy. The TEM and SEM images clearly illustrate that the NPs are mesoporous spheroids with an approximate diameter of 150 nm, both before and after calcination (**Figure** **1**a; Figure S1a–c, Supporting Information). The aberration‐corrected high‐angle annular dark‐field scanning transmission electron microscopy (HAADF‐STEM) images with atomic resolution reveal that individual copper atoms are well‐dispersed on the carbon matrix (indicated by red circles), with no detectable copper particles or clusters (Figure 1b). Similarly, energy‐dispersive spectroscopy (EDS) mapping indicates that Cu, C, and N elements are uniformly distributed within the NPs (Figure 1c). Furthermore, high‐resolution transmission electron microscopy (HRTEM) images reveal only visible graphite fringes and no copper metal streaks. The selected area electron diffraction (SAED) pattern shows no bright spots, indicating poor crystallinity (Figure 1d). The XRD pattern exhibits only the (002) and (101) planes of amorphous graphitic carbon, with no peaks corresponding to metallic copper (Figure 1f).^[^ ^13^, ^34^ ^]^ These results confirm the successful synthesis of mCu‐SAE, and the NPs are composed of monoatomic copper and amorphous carbon structures without copper clusters. The Cu loading was determined by inductively coupled plasma mass spectrometry (ICP‐MS) to be 0.9 wt%, demonstrating the amount of Cu dispersed in mCu‐SAE. Furthermore, quantification via the dialysis method revealed that CBPV exhibited minimal copper ion liberation in the simulated ROS‐rich microenvironment, with a 4.5% release over 24 h in a 0.1 mM H_2_O_2_ aqueous solution (Figure S2, Supporting Information). This constrained release profile signifies robust chelation between Cu ions and nitrogen coordination sites, effectively impeding dissociation during catalytic processes.

The surface element valence and chemical composition of the prepared mCu‐SAE were assessed by XPS. As depicted in the high‐resolution spectra of Cu 2p and N 1s, the Cu 2p spectrum exhibits two prominent peaks at 932.6 and 934.4 eV, corresponding to Cu^+^ and Cu^2+^, respectively, indicating that copper ions in mCu‐SAE exist in both +1 and +2 oxidation states (Figure 1g).^[^ ^35^ ^]^ The N 1s spectrum of mCu‐SAE can be deconvoluted into four distinct peaks at 398.4, 399.8, 400.9, and 404.4 eV, attributable to pyridinic N, Cu‐N_x_, graphitic N, and oxidized N, respectively (Figure S3, Supporting Information). Among these, pyridinic‐N and graphitic‐N are the most abundant, which is advantageous for stabilizing the structure of Cu single atoms and enhancing catalytic activity.^[^ ^36^ ^]^ To further clarify the oxidation state of copper single atoms in mCu‐SAE, we conducted X‐ray absorption near‐edge structure (XANES) measurements on Cu foil, CuO, Cu_2_O, and mCu‐SAE. The results revealed that the XANES spectrum of mCu‐SAE is positioned intermediate between those of Cu_2_O and CuO, suggesting that the oxidation state of the copper single atoms lies within the +1 to +2 range (Figure 1h). This is consistent with the findings from the XPS experiment. Subsequently, the extended X‐ray absorption fine structure (EXAFS) was analyzed to clarify the coordination environment of the Cu sites. As shown in Figure 1i, the first coordination shell is located at ≈1.5 Å, suggestive of Cu─N or Cu─O coordination. Furthermore, no significant Cu─Cu scattering was detected at 2.2 Å for mCu‐SAE, confirming that the Cu atoms exist in an isolated, single‐atom form. This finding is in agreement with the HAADF‐STEM results. To gain further structural insights, we performed least‐squares fits to the R‐space Fourier‐transformed EXAFS (FT‐EXAFS) plots of Cu foil, CuO, Cu_2_O, and mCu‐SAE (Figure 1j; Figure S4, Supporting Information). The k‐space EXAFS data were fitted (Figure 1k and l; Figure S5, Supporting Information) with the optimized parameters detailed in Table S1. The best‐fit results indicate that the dominant peak at ≈1.5 Å is assigned to the first coordination shell of Cu─N. The calculated average coordination number of Cu─N in mCu‐SAE is ≈ 3, indicating that each Cu atom is coordinated with three N atoms in the mCu‐SAE structure. Moreover, a wavelet transform (WT) analysis based on the EXAFS results was performed to differentiate the backscattering atoms (Figure 1m; Figure S6, Supporting Information). The analysis revealed the presence of only Cu─N coordination peaks in mCu‐SAE, with no detectable Cu─Cu coordination peaks. The WT experimental data further confirmed that the Cu atoms in mCu‐SAE are fully dispersed. To further validate the formation of a Cu‐N_3_ coordination structure in mCu‐SAE instead of the conventional Cu‐N_4_ configuration, we performed electron spin resonance (ESR) spectroscopy on single‐atom specimens pre‐ and post‐acid etching. The spectra revealed a distinct g = 2.003 signal corresponding to nitrogen vacancies exclusively in etched samples, indicating that the hydrochloric acid treatment of calcinated samples induces nitrogen atom liberation, thereby reconstituting the metal coordination sphere (Figure S7a, Supporting Information).^[^ ^37^ ^]^ Additionally, comparative N 1s XPS analysis demonstrated that structural nitrogen loss originated predominantly from N‐(C)_3_ moieties, definitively identifying the active site as Cu‐N_3_ (Figure S7b, Supporting Information). Furthermore, we employed Raman spectroscopy to investigate the effect of copper atom coordination on the graphitic structure of the carrier. The I_D_/I_G_ ratios of mCu‐SAE and NMCNPs are comparable, suggesting that the coordination of copper atoms does not significantly alter the graphitic structure (Figure S8, Supporting Information).

To analyze the mesopore size of mCu‐SAE, we conducted N_2_ adsorption/desorption isotherms (Figure S9, Supporting Information). The Brunauer‐Emmett‐Teller (BET) surface area of mCu‐SAE was measured to be 561.6 m^2^ g^−1^. The pore size distribution, calculated using the Barrett‐Joyner‐Halenda (BJH) model, revealed an average mesopore size of 5.5 nm for mCu‐SAE. These results demonstrate that mCu‐SAE possesses a substantial specific surface area and mesoporous structure, which facilitates drug loading and increases the number of available active copper sites, ultimately enhancing its catalytic activity for POD‐mimic and OXD‐mimic functions.

Subsequently, BNN6 was encapsulated into mCu‐SAE via host‐guest interactions to afford mCu‐SAE@BNN6 (CB), and UV–vis spectroscopy provides substantiation for the successful loading of BNN6. As depicted in Figure 1n, compared to mCu‐SAE, CB and CBPV exhibit an additional absorption peak at 260 nm, signifying the effective loading of BNN6 onto mCu‐SAE.^[^ ^38^ ^]^ The Fourier transform infrared (FT‐IR) spectroscopy also furnishes additional evidence for the successful BNN6 loading. The characteristic N─N═O peak of BNN6 is present at 1400 cm^−1^ in the infrared spectra of both CB NPs and CBPV. In contrast, this peak is absent in the spectra of mCu‐SAE alone (Figure S10, Supporting Information).^[^ ^38^ ^]^ Moreover, by establishing a BNN6 concentration‐absorbance standard curve, we evaluated the drug loading efficacy of using the anti‐solvent crystallization method versus conventional physical agitation. Quantitative analysis revealed a BNN6 loading content of 54.1 wt% for the crystallization protocol‐a 4.8‐fold enhancement over the traditional method (11.2 wt%) (Figure S11, Supporting Information).

The targeting moiety PEG‐Van is synthesized through amide‐bonding conjugation (Figure S12, Supporting Information).^[^ ^39^ ^]^ The UV–Vis spectrum shows an additional absorption peak at 280 nm, indicating the successful conjugation of MPEG_5000_‐NH_2_ and Van to obtain PEG‐Van (Figure S13a, Supporting Information).^[^ ^40^ ^]^ In addition, the FT‐IR spectrum of PEG‐Van displays amide I, amide II, and amide III bands at 1568, 1555, and 1236 cm^−1^, respectively, further corroborating the successful formation of amide bonds after conjugation (Figure S13b, Supporting Information).^[^ ^41^ ^]^ Furthermore, spectrophotometric quantification via a PEG‐Van concentration‐absorbance standard curve established a loading content of 7.5 ± 2.7 wt% (12.7 ± 4.9 nmol mg^−1^) in CBPV (Figure S14, Supporting Information). The surface decoration of PEG‐Van on mesoporous carbon nanospheres improves biocompatibility and adds bacteria‐targeting capabilities. The TEM and SEM images reveal that the CBPV have a smooth spherical surface due to the BNN6 loading and a thin layer of PEG‐Van coating, whereas the mCu‐SAE NPs exhibit a concave surface (Figure 1e; Figure S1d, Supporting Information). Furthermore, we analyzed the elemental mapping images of CBPV. We found that, in addition to the three elements of mCu‐SAE (C, N, and Cu), the characteristic element Cl of Van was also present, confirming the successful coating of PEG‐Van on mCu‐SAE (Figure S15, Supporting Information).^[^ ^42^ ^]^ The PEG‐Van coating led to an increase in the total hydrodynamic size of the NPs. Dynamic light scattering (DLS) measurements (Cu/PDA/F127: 247.7 ± 5.7 nm, mCu‐SAE: 217.5 ± 3.8 nm, CBPV: 284.2 ± 3.0 nm) confirmed this observation and demonstrated that CBPV has good stability in water (Figure S16, Supporting Information).

### 2.2 Photothermal Effect and NO Release of CBPV

**Figure 2:**
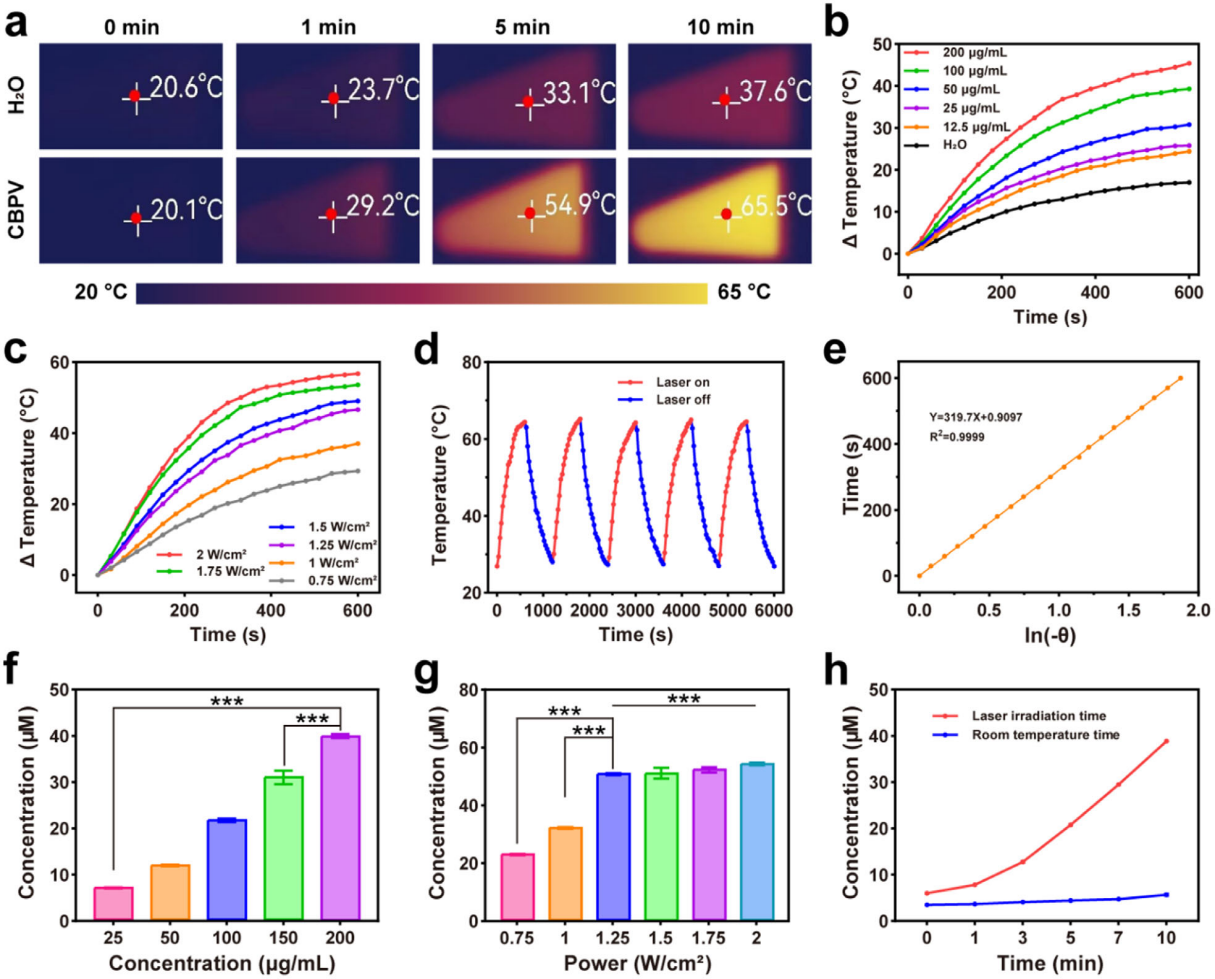
Photothermal effect and NO release of the CBPV. a) Real‐time photothermal images of CBPV (200 µg mL^−1^) under 1064 nm laser irradiation. b) Temperature change curves for different concentrations of CBPV irradiated by a laser (1.25 W cm^−2^). c) Temperature change curve of CBPV (200 µg mL^−1^) under laser irradiation with varying power densities. d) Temperature fluctuations of CBPV during five on/off cycles of laser irradiation at 1.25 W cm^−2^. e) The relationship between time and the negative natural logarithm of temperature (ln(‐θ)). f) NO release from different concentrations of CBPV after 10 min of laser irradiation (1.25 W cm^−2^). g) NO release from CBPV (200 µg mL^−1^) under laser irradiation with different power densities. h) NO release from CBPV (200 µg mL^−1^) under varying laser irradiation times (1.25 W cm^−2^) and incubation times at room temperature. Data are presented as mean ± SD (*n* = 3). ^***^ *p* < 0.001.

We first evaluated the optical properties of CBPV using UV–vis‐NIR spectroscopy. CBPV at various concentrations exhibits significant absorption in the NIR‐II region, implying that they may have the photothermal conversion capability triggered by NIR‐II irradiation (Figure S17, Supporting Information). Subsequently, we conducted a detailed investigation of the photothermal performance of CBPV. As illustrated in **Figure** **2**a,b, when exposed to a 1064‐nm laser with a power of 1.25 W cm^−2^ for 10 min, the temperature of the aqueous dispersion of CBPV (200 µg mL^−1^) increased to 65.5 °C, demonstrating a pronounced concentration‐dependent behavior, while the temperature of pure water exhibited only a marginal change. Notably, under the identical laser irradiation condition, the equivalent concentration of mCu‐SAE (85 µg mL^−1^) exhibited a temperature elevation from room temperature to 66.0 °C (Figure S18, Supporting Information). This comparable photothermal conversion efficiency confirms that the carbon matrix retains its intrinsic photothermal properties after the BNN6 loading and PEG‐Van coating. Furthermore, we investigated the effect of laser power on the heating performance of the NPs and found that as the laser power increased, the maximum temperature rise of CBPV also increased (Figure 2c). These results indicate that the carbon framework exhibits exceptional photothermal performance following high‐temperature calcination. Moreover, after five heating‐cooling cycles, the heating effect of CBPV remained almost unchanged, demonstrating their excellent photothermal stability (Figure 2d). Based on established methods in the literature report, we calculated the photothermal conversion efficiency (η) of CBPV under 1064 nm laser irradiation to be 43.9% (Figure 2e).^[^ ^43^ ^]^

BNN6 is a well‐recognized NO donor. Under high‐temperature conditions, the nitrosyl moiety of BNN6 decomposes to release NO.^[^ ^44^ ^]^ We employed both the Griess reagent kit and the 4‐Amino‐5‐Methylamino‐2′,7′‐Difluorofluorescein Diacetate (DAF‐FM DA) probe to assess the NO release behavior of CBPV under photothermal excitation. Initial NO quantification standardization was established via the Griess reagent calibration (Figure S19, Supporting Information). A 1064 nm laser at 1.25 W cm^−2^ was used to irradiate varying concentrations (25, 50, 100, 150, and 200 µg mL^−1^) of CBPV for 10 min. Subsequent measurements and calculations revealed that the NO release ranged from 6.8 to 39.5 µM (Figure 2f). We then investigated the effect of different laser powers on NO release and found that the amount of NO released increased with the elevated power. However, this proportional relationship significantly diminished when the laser power reached 1.25 W cm^−2^ (Figure 2g). Comparative analysis of the NPs with and without laser irradiation showed that CBPV (200 µg mL^−1^) released 38.8 µM of NO after 10 min of laser irradiation, which is markedly higher than the 5.7 µm released by the non‐irradiated NPs (Figure 2h). This observation indicates that the photothermal effect generated by laser irradiation promotes the decomposition of nitroso groups in BNN6 and the subsequent release of NO. Moreover, to verify the accuracy of NO quantification, we utilized the NO‐specific fluorescent probe DAF‐FM DA for real‐time monitoring of NO liberation. Quantitative analysis using the fluorescence intensity ratio (F_c_/F_0_) demonstrates that NO generation exhibits significant concentration dependence on the reactive material (Figure S20, Supporting Information), where F_c_ and F_0_ represent fluorescence intensities at the test concentration and baseline (0 µg mL^−1^), respectively. This concentration‐response relationship further validated the previous Griess assay measurements of the NO release. Additionally, we discovered that CBPV can slowly and continuously release NO at 37 °C (Figure S21, Supporting Information). This phenomenon is highly advantageous, as low NO concentrations benefit angiogenesis in the later stages of diabetic wound healing.

These results demonstrate that CBPV possess significant photothermal conversion capabilities, efficiently transforming NIR‐II laser energy into heat. This capability enables PTT‐based antibacterial treatment. Furthermore, the photothermal conversion elevates the temperature of the reaction system, facilitating a substantial and controlled release of NO, which, in conjunction with PTT, could effectively inhibit bacterial growth.

### 2.3 POD‐Like and OXD‐Like Activities of CBPV

**Figure 3:**
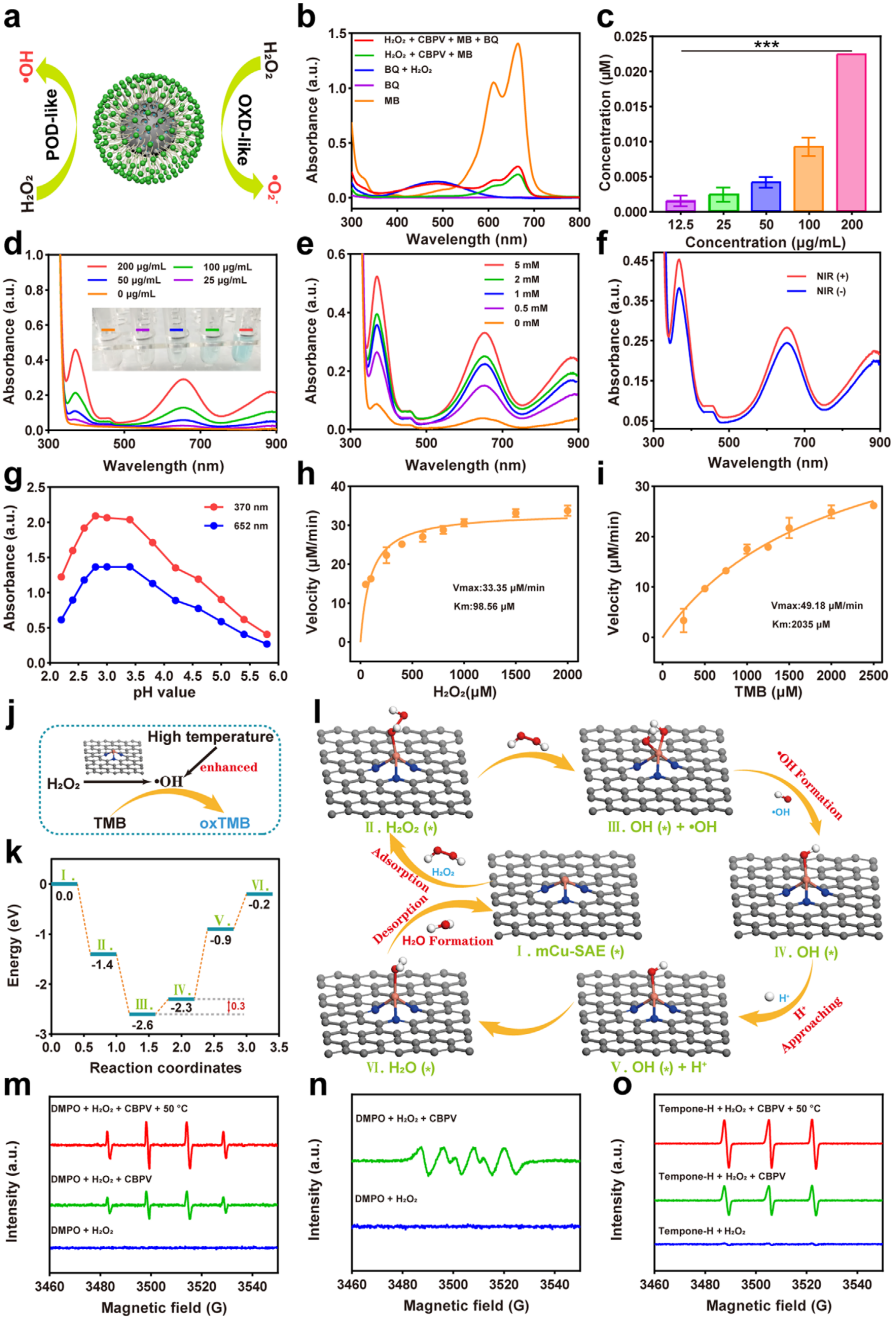
Evaluation of the enzyme‐like activities of CBPV. a) Schematic diagram illustrating the mechanism of enzyme‐like catalytic reactions. b) The degradation process of MB in the presence of BQ. c) Measurement of •O_2_^−^ yield from CBPV at different concentrations using an •O_2_^−^ detection kit. d) UV–vis absorption spectra of TMB oxidized by CBPV at different concentrations, with an inset showing the colorimetric reaction of TMB. e) UV–vis absorption spectra of TMB oxidized by CBPV (200 µg mL^−1^) and different concentrations of H_2_O_2_. f) UV–vis absorption spectra of TMB oxidation by CBPV with and without laser irradiation. g) UV absorbance values of TMB at 370 and 652 nm at different pH conditions. Steady‐state kinetic assay of CBPV for h) H_2_O_2_ and i) TMB substrate. j) Schematic diagram of the activity of POD‐like enzymes in mCu‐SAE. k) The free energy diagrams of mCu‐SAE during the catalytic process in an acidic environment. l) Schematic of the catalytic mechanism of mCu‐SAE in an acidic environment. m) ESR spectra of •OH detected using DMPO under different conditions. n) ESR spectra of •O_2_^−^ detected using DMPO. o) ESR spectra of ONOO^−^ detected using Tempone‐H under different conditions. Data are presented as mean ± SD (*n* = 3). ^***^ *p* < 0.001.

CBPV exhibits catalytic activities reminiscent of both POD‐like and OXD‐like, as illustrated by the enzyme‐mimetic catalytic mechanisms in **Figure** **3**a. 1,4‐Benzoquinone (BQ) is frequently utilized to scavenge •O_2_^−^ and is therefore commonly employed to determine whether NPs exhibit OXD enzyme activity.^[^ ^45^ ^]^ Upon the addition of BQ to the CBPV + H_2_O_2_ group, a significant increase in the absorbance of MB at 664 nm was observed, indicating that BQ effectively scavenged the generated •O_2_^−^ in the reaction system. This observation indirectly demonstrates that CBPV possess OXD enzyme activity (Figure 3b). Additionally, we evaluated the OXD enzyme activity of CBPV using a Superoxide Anion Activity Content Assay Kit and quantified the •O_2_^−^ release behavior of the NPs by establishing a •O_2_^−^ standard curve (Figure S22, Supporting Information). After mixing different concentrations of CBPV (12.5, 25, 50, 100, and 200 µg mL^−1^) with H_2_O_2_ and incubating them in a shaker at 37 °C for a specified duration, the •O_2_^−^ release was detected using the assay kit, ranging from 0.0015 to 0.0224 µm (Figure 3c). These experimental results indicate that CBPV can catalyze the generation of •O_2_^−^ from H_2_O_2_.

To assess the POD‐like biocatalytic activity of CBPV, we characterized their ability to decompose H_2_O_2_ and generate •OH in a mildly acidic environment (pH = 6.5) using 3,3′,5,5′‐tetramethylbenzidine (TMB) as an indicator. •OH can oxidize TMB to produce a blue product, oxTMB, characterized by prominent absorbance at 370 and 652 nm.^[^ ^46^ ^]^ With increasing concentrations of mCu‐SAE, CBPV, and the substrate H_2_O_2_, the reaction system in a mildly acidic environment exhibits a colorimetric response, with two distinct absorption peaks at 370 and 652 nm observable in the UV–vis‐NIR spectrum (Figure 3d and e; Figure S23a,b, Supporting Information). This finding indicates that mCu‐SAE and CBPV can catalyze the generation of •OH from H_2_O_2_. Notably, at equivalent mCu‐SAE concentrations, CBPV and mCu‐SAE exhibit comparable POD‐like kinetics, implying that the BNN6 encapsulation and PEG‐Van functionalization didn't significantly affect the integrity of catalytic active sites. Furthermore, we verified the POD‐like biocatalytic activity of CBPV using methylene blue (MB) as a colorimetric substrate. With increasing NP concentration, the absorbance of MB at 664 nm gradually diminishes, indicating the generation of more reactive ROS, which results in MB oxidation (Figure S24, Supporting Information).^[^ ^47^ ^]^ These results underscore that CBPV exhibits notable POD‐like biocatalytic activity, capable of catalyzing the generation of •OH from H_2_O_2_, with a concentration‐dependent effect. Notably, we observed that the temperature significantly influences the generation of •OH. Using NIR‐II laser irradiation to increase the reaction environment temperature, it was found that more oxTMB was detected by UV–vis spectroscopy compared to the group without laser irradiation, indicating that the thermal effect can enhance the biocatalytic reaction (Figure 3f).

Additionally, we performed steady‐state kinetic assays to characterize the POD‐like activity of CBPV. The pH‐dependent activity profiling identified the optimal catalytic efficiency at pH 2.8 (Figure 3g). Subsequent Michaelis‐Menten kinetics analysis at this pH optimum quantified both H_2_O_2_ concentration‐dependent TMB oxidation (Figure 3h) and TMB concentration‐dependent reaction kinetics (Figure 3i). Lineweaver‐Burk transformations of Beer‐Lambert‐derived initial velocities yielded Michaelis constants (K_m_) of 98.6 µM for H_2_O_2_ and 2035.0 µM for TMB, with corresponding maximum velocities (V_max_) of 33.4 µM min^−1^ and 49.2 µM min^−1^. These V_max_ values are significantly higher than those of well‐studied Fe_3_O_4_/CDs, Cu_3_P/CDs, and FeP nanozymes (Table S2, Supporting Information), verifying the potent POD‐like activity of CBPV.

The POD‐like activity pathway of mCu‐SAE is illustrated in Figure 3j. To further investigate the POD‐like catalytic mechanism of mCu‐SAE in a slightly acidic environment, we conducted density functional theory (DFT) calculations to elucidate the atomic‐level catalytic process. As shown in Figure 3k, l, the geometrically optimized H_2_O_2_ molecule was readily adsorbed at the single‐atom Cu site of mCu‐SAE (I.), forming an activated H_2_O_2_* (II.) with an adsorption energy of 1.4 eV. The activated H_2_O_2_ molecule was then cleaved by the single‐atom Cu site, producing a reactive •OH radical and a hydroxyl group (OH*) bound to the Cu site (III.). Subsequently, the reactive •OH (IV.) was released, a critical step that involves an energy barrier of 0.3 eV, which can be easily overcome at room temperature. In contrast, the energy barrier for the Cu‐N_4_ active site in the same •OH‐releasing step was reported to exceed 0.5 eV,^[^ ^3^ ^]^ implying that the Cu‐N_3_ site has a better POD‐like catalytic activity. Subsequently, in a weakly acidic environment, a protonated hydrogen atom approached the remaining OH* (V.), forming an adsorbed H_2_O molecule (H_2_O*) (VI.) on the mCu‐SAE surface. The subsequent desorption of H_2_O* restored mCu‐SAE to its initial state (I.), and the total Gibbs free energy of the whole POD‐like reaction decreased significantly (‐0.9 eV). After this cycle, mCu‐SAE underwent the next cycle of catalyzing H_2_O_2_ to generate •OH, thereby exhibiting efficient POD‐like catalytic activity.

Based on the previous experimental results, it is evident that CBPV can slowly release NO at room temperature or 37 °C and release a substantial amount of NO at elevated temperatures. The released NO can react with •OH and •O_2_^−^ generated from the decomposition of H_2_O_2_ to form ONOO^−^, which exhibits high antimicrobial activity. To monitor the generation of ONOO^−^, we employed the dihydrorhodamine 123 (DHR123) fluorescent probe, which demonstrates maximal sensitivity and oxidation efficiency toward ONOO^−^ among various ROS, yielding the most intense fluorescence signal.^[^ ^48^ ^]^ The CBPV treatment group exhibited significantly enhanced fluorescence intensity relative to controls, confirming robust ONOO^−^ formation via reactive coupling between photogenerated ROS and NO. This peroxynitrite yield was further amplified under laser irradiation (Figure S25, Supporting Information). To further substantiate the generation of •OH, •O_2_^−^, and ONOO^−^, ESR spectroscopy was employed for detection. As depicted in Figure 3m–o and 5,5‐dimethyl‐1‐pyrroline N‐oxide (DMPO) was utilized as a capturing agent for •OH and •O_2_^−^, while Tempone‐H was used to detect the formation of ONOO^−^. In the reaction system containing only the scavenger and H_2_O_2_, no ESR signals were observed, indicating that the presence of the capturing agent and H_2_O_2_ alone does not generate these ROS. Upon adding CBPV, the ESR spectra exhibited 1:2:2:1, 1:1:1:1, and 1:1:1 signal peaks, respectively, confirming that the NPs catalyze the generation of •OH, •O_2_^−^, and ONOO^−^.^[^ ^49^ ^]^ Notably, when the temperature of the reaction system increased from 25 to 50 °C, more intense •OH and ONOO^−^ signal peaks were detected, suggesting that temperature enhancement significantly augments the POD‐like activity of CBPV.

### 2.4 Antibacterial and Antibiofilm Activities of CBPV

Encouraged by the exceptional photothermal conversion ability of CBPV and the capacity to generate highly antibacterial ONOO^−^, we investigated their in vitro antibacterial and anti‐biofilm properties. MRSA typically affects the early stages of infection, while *Escherichia coli* (*E. coli*) influences the depth of the infection, thereby delaying wound recovery.^[^ ^50^ ^]^ Consequently, we selected these two bacteria as the subjects of our study.

**Figure 4:**
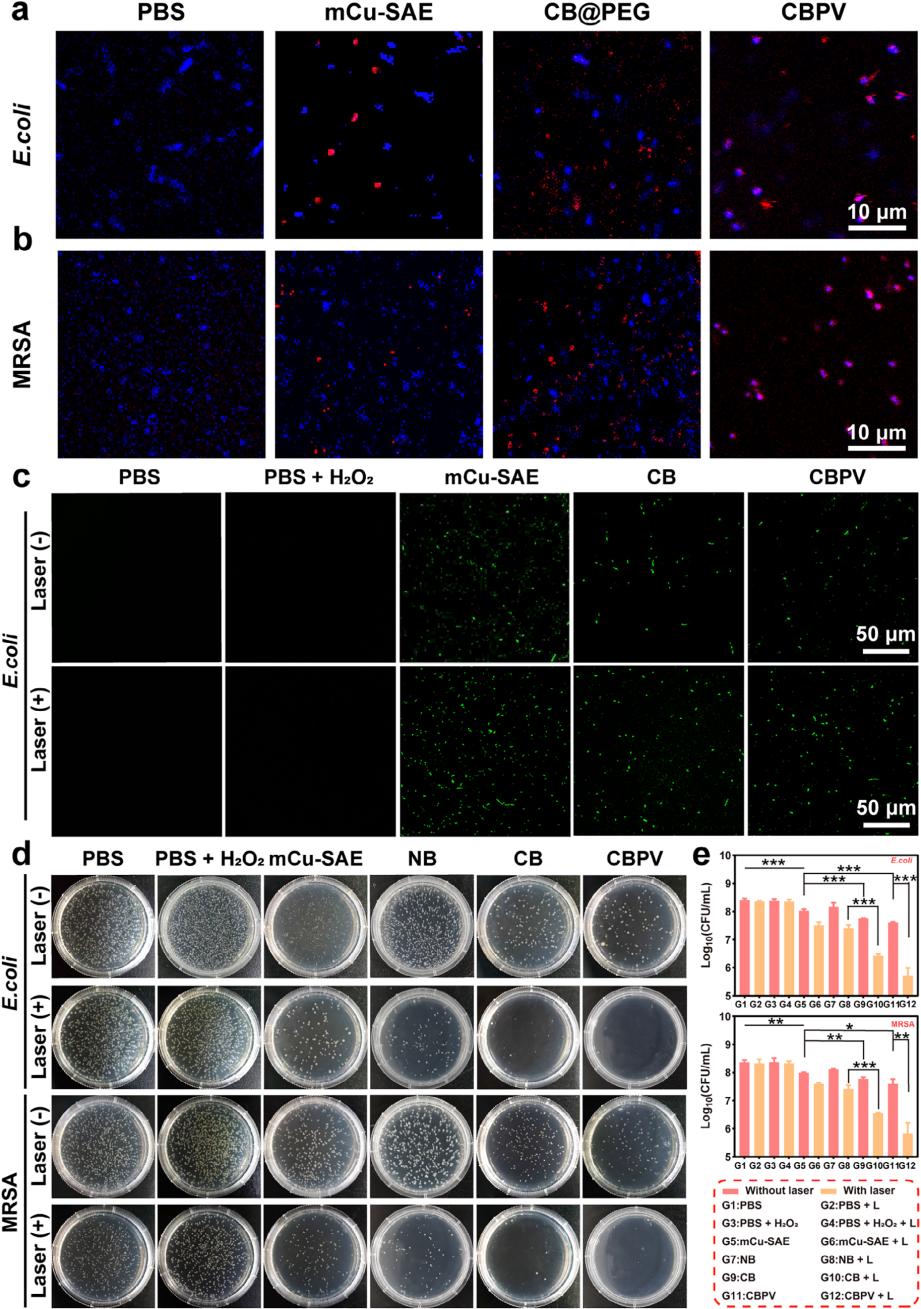
Antibacterial activity of CBPV in vitro. a) Confocal fluorescence images showing the interaction between CBPV and *E. coli*. Scale bar = 10 µm. (Blue fluorescence: DAPI; Red fluorescence: rhodamine). b) High‐resolution confocal fluorescence image of the interaction between CBPV and MRSA. Scale bar = 10 µm. (Blue fluorescence: DAPI; Red fluorescence: rhodamine). c) Fluorescence images of ROS in *E. coli* after various treatments. Scale bar = 50 µm. d) Evaluation of CBPV antibacterial activity against *E. coli* and MRSA by agar plate method. e) Colony‐forming unit (CFU) counts for *E. coli* and MRSA. Data are presented as mean ± SD (*n* = 3). *p < 0.05, **p < 0.01, ***p < 0.001.

Initially, we investigated the capability of mCu‐SAE, CB@PEG, and CBPV to target and capture bacteria. We first synthesized CB@PEG NPs (Figure S26, Supporting Information) and then used Rhodamine B (RhB)‐labeled mCu‐SAE, CB@PEG, and CBPV to incubate with the two bacteria. It is reported that Van targets bacteria by binding to precursor molecules essential for bacterial cell wall synthesis.^[^ ^27^ ^]^ Confocal laser scanning microscopy images revealed that mCu‐SAE and CB@PEG did not adhere to the bacteria due to the lack of targeting Van moiety. In contrast, CBPV with surface‐anchored Van ligands exhibited extensive overlap of red (represented for CBPV) and blue (represented for bacteria) fluorescence, indicating their ability to target and capture bacteria. This trend was evident for both MRSA and *E. coli* (**Figure** **4**a,b). The co‐localization rates of CBPV with *E. coli* and MRSA were quantified at 74.9% and 80.7%, respectively, suggesting that CBPV effectively captured the bacteria (Figure S27, Supporting Information). Subsequently, we quantified the levels of ROS in bacterial cells following various treatments. The NPs can generate copious amounts of ROS within the bacteria, leading to lysis or death. Using 2′,7′‐Dichlorofluorescein diacetate (DCFH‐DA) to label the generated ROS, confocal laser scanning microscopy images and their quantitative analysis demonstrated that the mCu‐SAE + L group exhibited the strongest green fluorescence, attributable to the enhanced enzymatic catalytic activity of the NPs due to thermal effects. The fluorescence intensity of the CB group and the CBPV group decreased sequentially, primarily due to a reduction in the proportion of total active metal sites caused by the loading of BNN6 and PEG‐Van while using the identical dosage of nanomaterials (Figure 4c; Figure S28, Supporting Information). Moreover, we quantitatively assessed ONOO^−^ generation in bacteria across different treatment groups. Confocal laser scanning microscopy with DHR123 fluorescent probing revealed significantly enhanced green fluorescence intensity in the CBPV + L group, indicating maximal ONOO^−^ production (Figure S29, Supporting Information). This phenomenon is mechanistically attributed to the photothermally potentiated generation of NO and ROS at elevated temperatures. Notably, the CBPV + L group outperformed the mCu‐SAE@PEG‐Van + L group by 4.2‐fold in fluorescence intensity, consistent with the exceptional sensitivity and signal amplification of DHR123 toward peroxynitrite derivatives.

Subsequently, the agar plate method was employed to evaluate in vitro antibacterial efficacy of each bactericidal pathway (Figure 4d). The number of colonies in the PBS group, PBS + H_2_O_2_ group, and their corresponding laser‐irradiated groups did not change, indicating that laser irradiation alone and H_2_O_2_ alone do not possess bactericidal effects. The addition of mCu‐SAE resulted in a mild antibacterial effect, attributed to the generation of ROS via mCu‐SAE catalyzing H_2_O_2_. When the mCu‐SAE group was subjected to 1064‐nm laser irradiation, the excellent photothermal properties of the NPs raised the temperature around the bacteria to ≈50 °C, significantly reducing bacterial viability. The bacterial killing rate reached ≈85%, demonstrating the superior antibacterial effect of PTT. To verify the potent antibacterial toxicity of ONOO^−^, mCu‐SAE loaded only with BNN6 (CB) was used to treat the bacteria, while NMCNPs loaded with BNN6 (NMCNPs@BNN6) was used as the Control. The antibacterial effect of the non‐laser group was significantly enhanced compared to the mCu‐SAE group and the NMCNPs@BNN6 group, indicating that the slowly released NO, in combination with •OH and •O_2_^−^ to form ONOO^−^, exhibits potent antibacterial properties. With the addition of laser irradiation, a substantial amount of ONOO^−^ is generated, which synergizes with the PTT effect, achieving a 99% antibacterial efficacy. In contrast, the antibacterial efficacy of combined NO and PTT treatment was assessed using NMCNPs@BNN6 under laser irradiation, which revealed ≈90% bacterial eradication, suggesting that NO‐PTT treatment is insufficient for complete bacterial elimination. These findings demonstrate the highly potent antibacterial effect of ONOO^−^. After conjugating with the targeting Van moiety, CBPV demonstrated antibacterial capabilities comparable to CB NPs, achieving a 99% antibacterial effect under laser irradiation (Figure 4e). The live/dead bacterial staining experiment further corroborated the antibacterial efficacy of CBPV. We employed SYTO‐9 and propidium iodide (PI) for the live/dead bacterial staining. SYTO‐9 stains both live and dead bacteria with green fluorescence, while PI selectively penetrates bacteria with compromised cell membranes. Upon entry into damaged bacteria, PI quenches the green fluorescence of SYTO‐9 and emits its characteristic red fluorescence.^[^ ^21a^ ^]^ The control groups (PBS, PBS + L, PBS + H_2_O_2_, and PBS + H_2_O_2_ + L) for both bacterial strains exhibited only green fluorescence with negligible red fluorescence, indicating that laser irradiation alone and H_2_O_2_ treatment do not significantly reduce bacterial viability. In contrast, the mCu‐SAE + L group, CB + L group, and CBPV + L group displayed substantial red fluorescence, signifying that most bacteria had been damaged or killed. Notably, the CB + L and CBPV + L groups showed virtually no green fluorescence, suggesting that the PTT effect of the NPs synergistically enhances the bactericidal activity of ONOO^−^ (Figure S30, Supporting Information).

**Figure 5:**
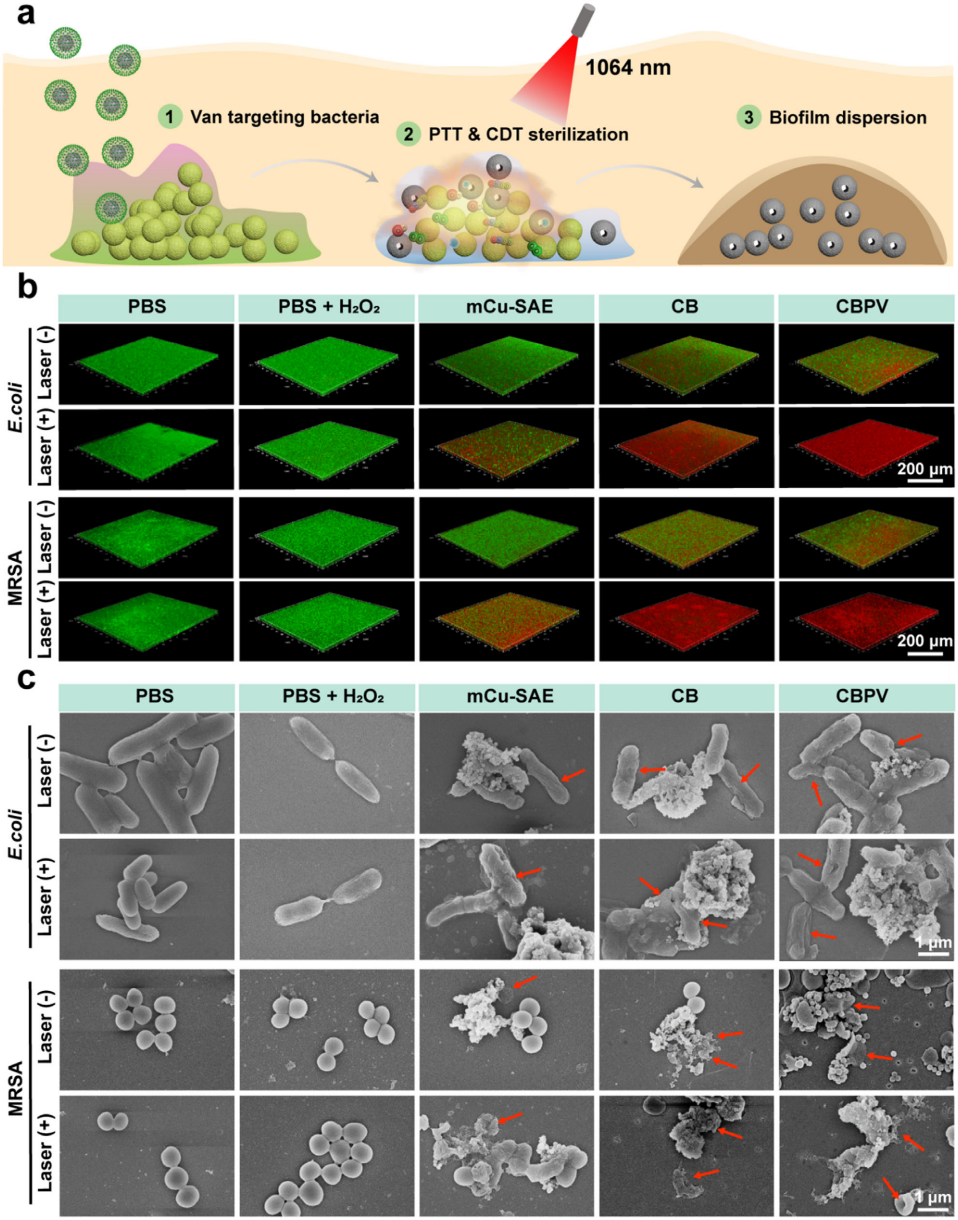
Evaluation of CBPV in disrupting bacterial biofilms. a) Schematic illustration of anti‐biofilm treatment by CBPV. b) Evaluation of anti‐biofilm effects against MRSA using SYTO‐9/PI dual fluorescence staining. Scale bar = 200 µm. c) SEM images of *E. coli* and MRSA following different treatments. Scale bar = 1 µm. (Red arrow: bacterial contraction and destruction).

Bacterial biofilms consist of 3D extracellular polymeric substances (EPS), which provide essential nutritional support for bacteria, facilitate bacterial surface adhesion, and play a critical role in shielding bacteria from antibiotics or host immune responses.^[^ ^51^ ^]^ Therefore, the ability to penetrate or disrupt biofilms is essential to effectively treat chronic infections. To evaluate the anti‐biofilm activity of CBPV, we treated mature biofilms with NPs, both with and without 1064 nm laser irradiation (**Figure** **5**a). We then evaluated the integrity of the biofilm after different treatments using SYTO‐9/PI staining assays. Confocal laser scanning microscopy revealed that the mCu‐SAE group had a modest impact on the integrity of the bacterial biofilm, which can be attributed to the destruction of bacteria and EPS by the •OH and •O_2_^−^ generated through catalysis. However, this effect was limited and did not significantly disrupt the biofilm. After introducing NO for antibacterial treatment, a substantial amount of red fluorescence was observed in the 3D images. This observation indicates that the highly toxic RNS, ONOO^−^, effectively eliminated bacteria and EPS in the biofilm, leading to significant biofilm disruption. This phenomenon became even more pronounced after laser irradiation due to the synergistic effects of PTT, ROS, and nitrogen species. On one hand, the photothermal effect significantly compromised the integrity of the biofilm. On the other hand, the elevated temperature enhanced the production of ONOO^−^. The combined effect led to the near‐complete eradication of bacteria and the complete degradation of EPS. The 3D biofilm images of the CB + L group and the CBPV + L group, characterized by predominantly red fluorescence, clearly demonstrated this effect (Figure 5b).

Next, we used SEM to examine the surface morphology of the bacteria after various treatments. As depicted in Figure 5c, the control groups, including PBS, PBS + L, PBS + H_2_O_2_, and PBS + H_2_O_2_ + L, exhibited smooth and intact bacterial surfaces. In contrast, starting from the mCu‐SAE group, the bacteria displayed morphological alterations such as wrinkling. More strikingly, in the CBPV + L group subjected to laser irradiation, the bacterial surfaces revealed numerous perforations and extensive leakage of intracellular contents. This observation further emphasizes the potent chemical and photothermal antibacterial properties of CBPV.

### 2.5 Cytotoxicity, Cell Migration, Angiogenic Promotion, and Hemocompatibility of CBPV

**Figure 6:**
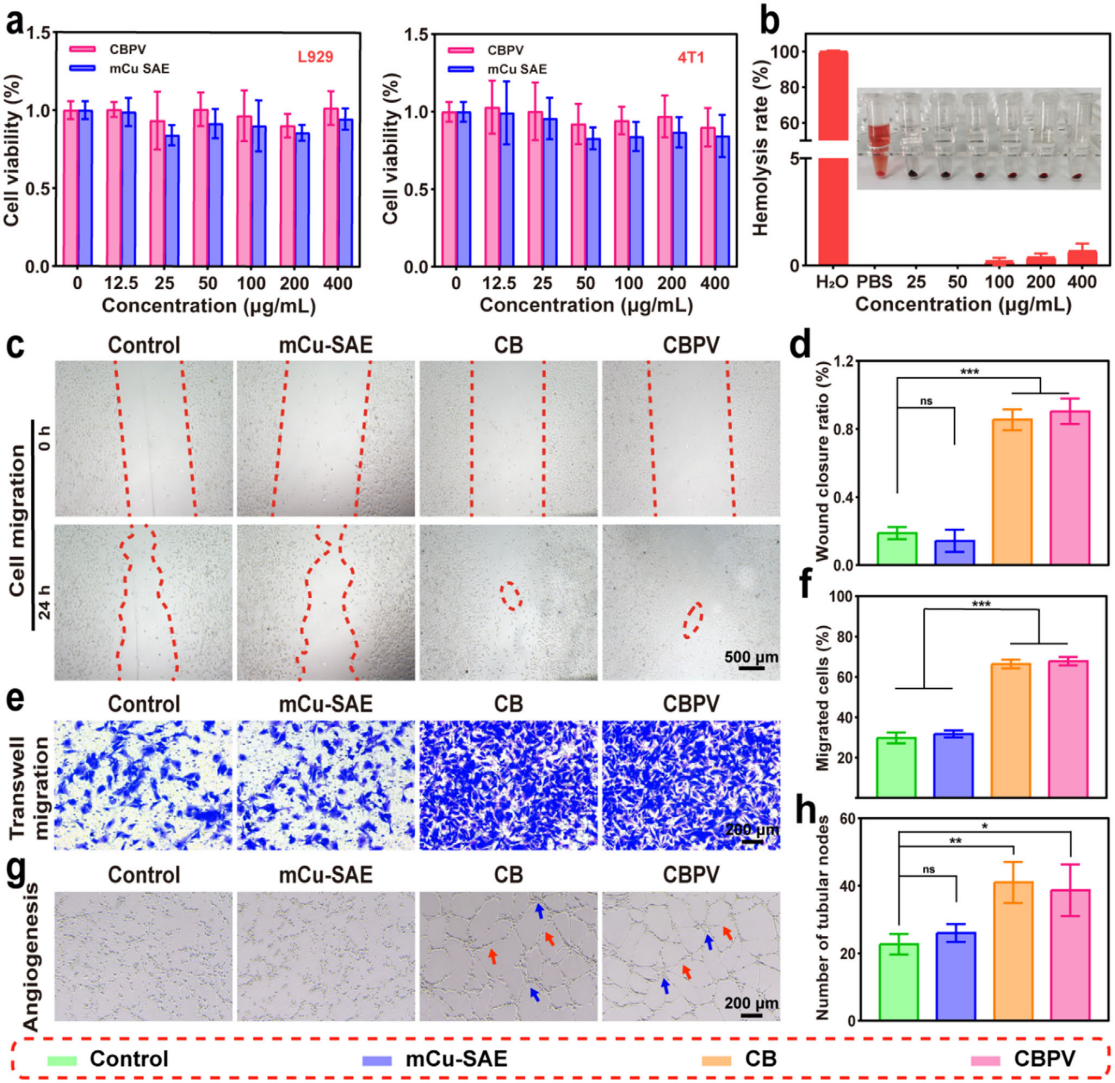
Biocompatibility, cell migration, and angiogenic promotion performance of CBPV. a) Cell viability of 4T1 and L929 cells after co‐incubation with different concentrations of mCu‐SAE and CBPV. b) Hemolysis results of CBPV at different concentrations, with ultrapure water as the positive control and PBS as the negative control. Inset: corresponding digital photographs. c) Images of L929 cell migration and d) quantitative analysis of wound closure rate after 24 h. Scale bar = 500 µm. e) Crystal violet staining image of HUVECs cell migration and f) quantification of HUVECs migration within 24 h. Scale bar = 200 µm. g) To evaluate the ability of HUVECs to form blood vessels in different treatment groups, and h) to provide quantitative statistics of the number of tubular nodes in different treatment groups. Scale bar = 200 µm. (Red arrow: blood vessels; Blue arrow: nodules). Data are presented as mean ± SD (*n* = 3). ns denotes no significance, *p < 0.05, **p < 0.01, ***p < 0.001.

The application of nanomaterials necessitates good biocompatibility, a crucial criterion for assessing their performance. To evaluate the biocompatibility of CBPV, we conducted the MTT colorimetric assay (**Figure** **6**a). Tumor cells (4T1) and normal cells (L929) were selected for this cytotoxicity study. The results from the MTT assay revealed that the viability of both 4T1 and L929 cells treated with 400 µg mL^−1^ of mCu‐SAE or CBPV exceeded 85%, indicating that the cytotoxicity of these materials is minimal, with negligible cell damage. Additionally, at equivalent concentrations, mCu‐SAE exhibited a higher cytotoxic effect than CBPV, which can be attributed to the enhanced biocompatibility of CBPV following PEG‐Van encapsulation. Furthermore, we assessed the blood compatibility of CBPV using a hemolysis assay. After incubating the materials with red blood cells at various concentrations (25, 50, 100, 200, and 400 µg mL^−1^) for a specified duration, the hemolysis rate did not exceed 5%, indicating that CBPV exhibit excellent blood compatibility (Figure 6b).

Cell migration serves as a crucial indicator of wound repair. To assess the wound‐healing efficacy of CBPV, we evaluated their influence on cellular motility through a cell scratch assay and a Transwell migration assay. As depicted in Figure 6c, a scratch wound was created and subsequently incubated with various materials. Following 24 h of treatment, the impact of both the Control group and the mCu‐SAE group on the migration of L929 cells was minimal. In contrast, the wound closure rates for the CB NPs group and the CBPV group were 85.5% and 90.4%, respectively, substantially exceeding the control group (18.8%) (Figure 6d). This phenomenon was also evident in the Transwell cell migration assay. The migratory capacity of HUVECs was significantly augmented in both the CB NPs and CBPV groups. Quantitative analysis revealed that the number of migrated cells in the CBPV group was ≈2.3 times greater than that in the control group (Figure 6e,f). This observation suggests that NO release from CBPV under physiological conditions plays a pivotal role in augmenting the migration of L929 cells to the wound site. Moreover, the pro‐angiogenic potential of CBPV was substantiated by the tube formation assay. As illustrated in Figure 6g, h, the co‐culture of HUVECs with CB NPs and CBPV resulted in a pronounced tubular network with a significantly higher number of tubular nodes than the control group. Collectively, these findings substantiate that CBPV enhance fibroblast migration and expedite angiogenesis at the wound site, thereby facilitating the wound healing process.

### 2.6 In Vivo Treatment of Epidermal Diabetic Wound Healing

**Figure 7:**
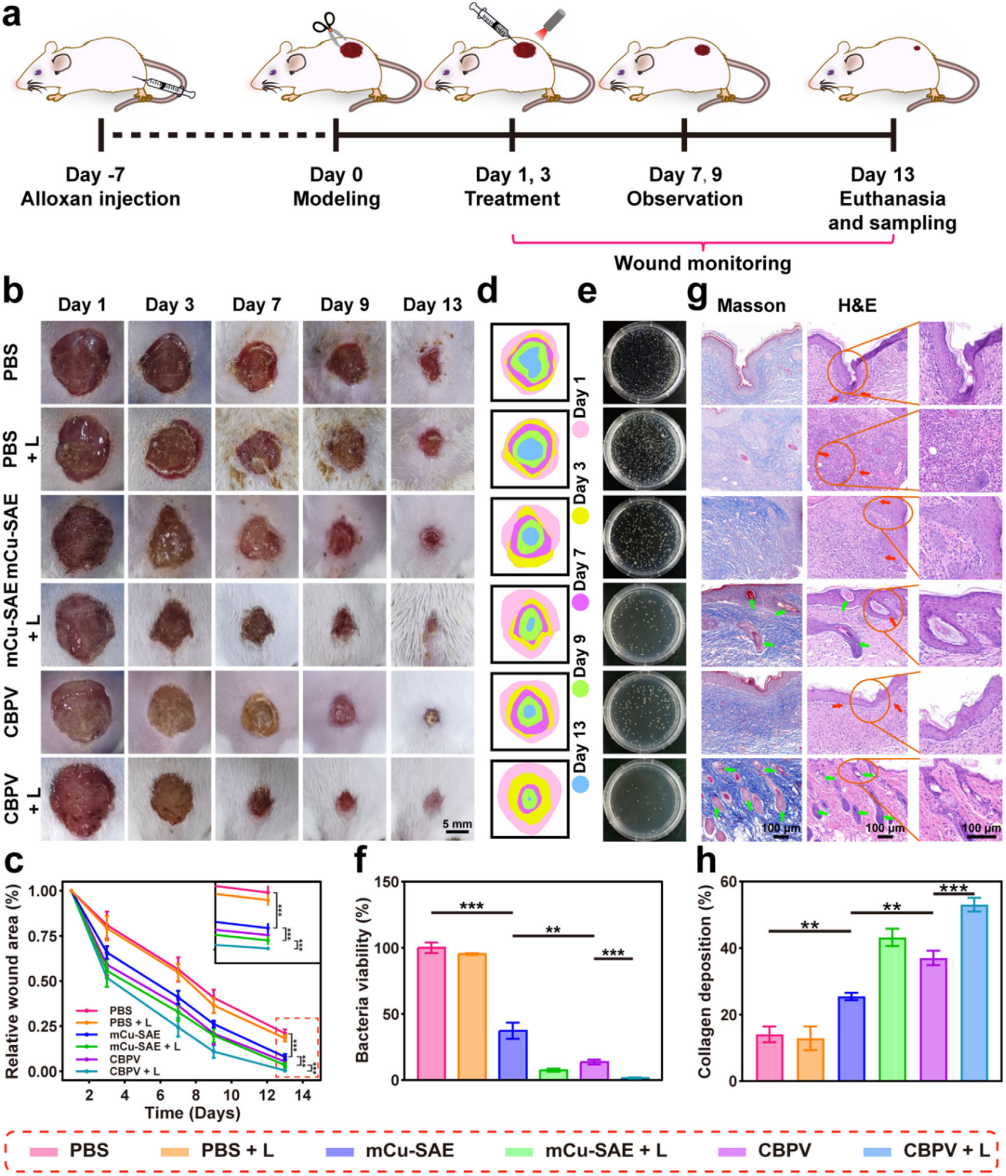
Efficacy of CBPV in diabetic epidermal wound healing. a) Establishment of a diabetic epidermal wound infection model and schematic representation of the treatment protocol. b) Representative images of wounds in different groups of mice at days 1, 3, 7, 9, and 13. Scale bar = 5 mm. c) Statistical analysis of the relative wound area in various groups (*n* = 6, mean ± SD). d) Wound trace simulation. e) Photographs of colony growth in wounds from different groups on day 3. f) Quantitative analysis of colony growth in wounds from different groups on day 3 (*n* = 3, mean ± SD). g) H&E staining and Masson's trichrome staining of regenerated skin on day 13. Scale bar = 100 µm. (Red arrow: inflammatory cells; Green arrow: hair follicles and glands). h) Quantitative analysis of collagen deposition in the epidermal wound model (*n* = 3, mean ± SD). **p < 0.01, ***p < 0.001.

To investigate the in vivo antibacterial effects of CBPV, we established a model of MRSA‐infected diabetic epidermis and subcutaneous cysts. First, we evaluated the epidermal antibacterial capability of the NPs, with the entire treatment process illustrated in **Figure** **7**a. The wounds were inoculated with MRSA on day 0, followed by treatment of the epidermal wounds in the mice on days 1 and 3. During the treatment, a thermal infrared imaging camera was employed to monitor the temperature of the wound area. After 10 min of laser irradiation, the PBS + L group, mCu‐SAE + L group, and CBPV + L group exhibited temperatures of 42.2, 51.8, and 51.6 °C, respectively (Figure S31, Supporting Information). This result indicates that CBPV can generate desired PTT effects. Photographs of the mouse wounds were captured on days 1, 3, 7, 9, and 13 (Figure 7b). The images reveal that mice treated with mCu‐SAE alone had smaller wound areas than the two control groups (PBS and PBS + L). This observation suggests that the •OH and •O_2_^−^ generated by mCu‐SAE catalysis exerted a bactericidal effect. Compared to the mCu‐SAE group, the CBPV group showed markedly smaller wound areas, indicating that the ONOO^−^ produced by CBPV exhibits higher toxicity against bacteria. Following the laser irradiation, the wound areas were reduced relative to those in mice that did not receive laser treatment, which can be attributed to the significant bactericidal effect of PTT on the bacteria in the wounds. The quantification of the wound areas provides a more direct demonstration of the in vivo antibacterial effects of CBPV (Figure 7c). Due to the bactericidal effects of ROS and PTT, from the third day of treatment, the wound areas in the mCu‐SAE group, mCu‐SAE + L group, CBPV group, and CBPV + L group were significantly smaller than those in the two control groups. This difference became even more pronounced on day 7, with the mCu‐SAE + L group exhibiting a 67.1% reduction in the wound area and the CBPV + L group achieving a 75.6% reduction, whereas the PBS group showed only a 45.3% reduction. By the end of the treatment on day 13, the wounds in the CBPV + L group were nearly fully healed, with only 0.4% of the wound area remaining unhealed, whereas the control group still had 18.3% of the unhealed wound area. Analysis of the simulated wound images from each group revealed that the CBPV + L group demonstrated a markedly superior wound infection treatment effect (Figure 7d). Moreover, on the third day, exudates from the wound surfaces were collected and spread on agar plates to evaluate the residual bacterial levels in each group (Figure 7e,f). Notably, the final bacterial residue in the CBPV + L group was 1.6%, significantly lower than the 37.3% in the mCu‐SAE group, indicating that the combination of PTT and ONOO^−^ can effectively eradicate MRSA in the wounds and promote wound healing.

Histological analysis of regenerating skin at the wound site was conducted to evaluate the healing of diabetic wounds. On the third day of treatment, skin samples from each group of mice were collected and subjected to hematoxylin and eosin (H&E) staining. The control groups (PBS and PBS + L) exhibited severe inflammatory infiltration and tissue damage, indicating a significant bacterial infection in the wounds. The inflammatory conditions in the mCu‐SAE and CBPV groups progressively improved, suggesting that the generated ROS exerted a bactericidal effect, thereby reducing inflammation at the wound site. Following exposure to 1064 nm laser irradiation, the inflammatory conditions further improved. The mCu‐SAE + L group showed a substantial reduction in inflammatory factors compared to the mCu‐SAE group, while the inflammatory factors in the CBPV + L group were virtually eliminated (Figure S32, Supporting Information). These results indicate that the synergistic effect of ONOO^−^ and PTT can effectively eradicate bacteria and thereby ameliorate inflammatory conditions in the body.

Skin samples harvested on treatment days 7 and 13 underwent H&E and Masson's trichrome staining to evaluate the healing progression (Figure 7g, Figure S33, Supporting Information). Histopathological analysis revealed time‐dependent epithelial restitution and collagen maturation. Notably, CBPV + L groups exhibited a marked enhancement in dermal architecture restoration and collagen deposition relative to controls, indicating accelerated tissue remodeling. On the thirteenth day, the CBPV + L group displayed the most complete epidermal repair, with hair follicles, glands, and tissue fibers fully regenerated and almost no inflammatory infiltration. The Masson's trichrome staining results also indicated that the final group had the highest collagen deposition. Quantitative analysis revealed that the collagen content in the final group was 53.0%, which is ≈39.0% higher than that in the control group (Figure 7h). Collagen plays a crucial role in skin tissue regeneration and angiogenesis. These findings demonstrate that CBPV are highly effective in treating epidermal infections and promoting wound recovery.

On the seventh day, we collected epidermal samples for immunofluorescence and immunohistochemical staining to observe changes in inflammatory markers and the formation of blood vessels in the skin tissue. Immunohistochemistry revealed that the levels of pro‐inflammatory cytokines IL‐6 and TNF‐α were significantly lower in the treatment groups compared to the control group post‐treatment. Notably, the CBPV + L group exhibited the lowest residual levels of IL‐6 and TNF‐α, at only 5.8% and 8.5%, respectively. This indicates that CBPV can effectively mitigate inflammation induced by bacterial infection (Figure S34, Supporting Information).

**Figure 8:**
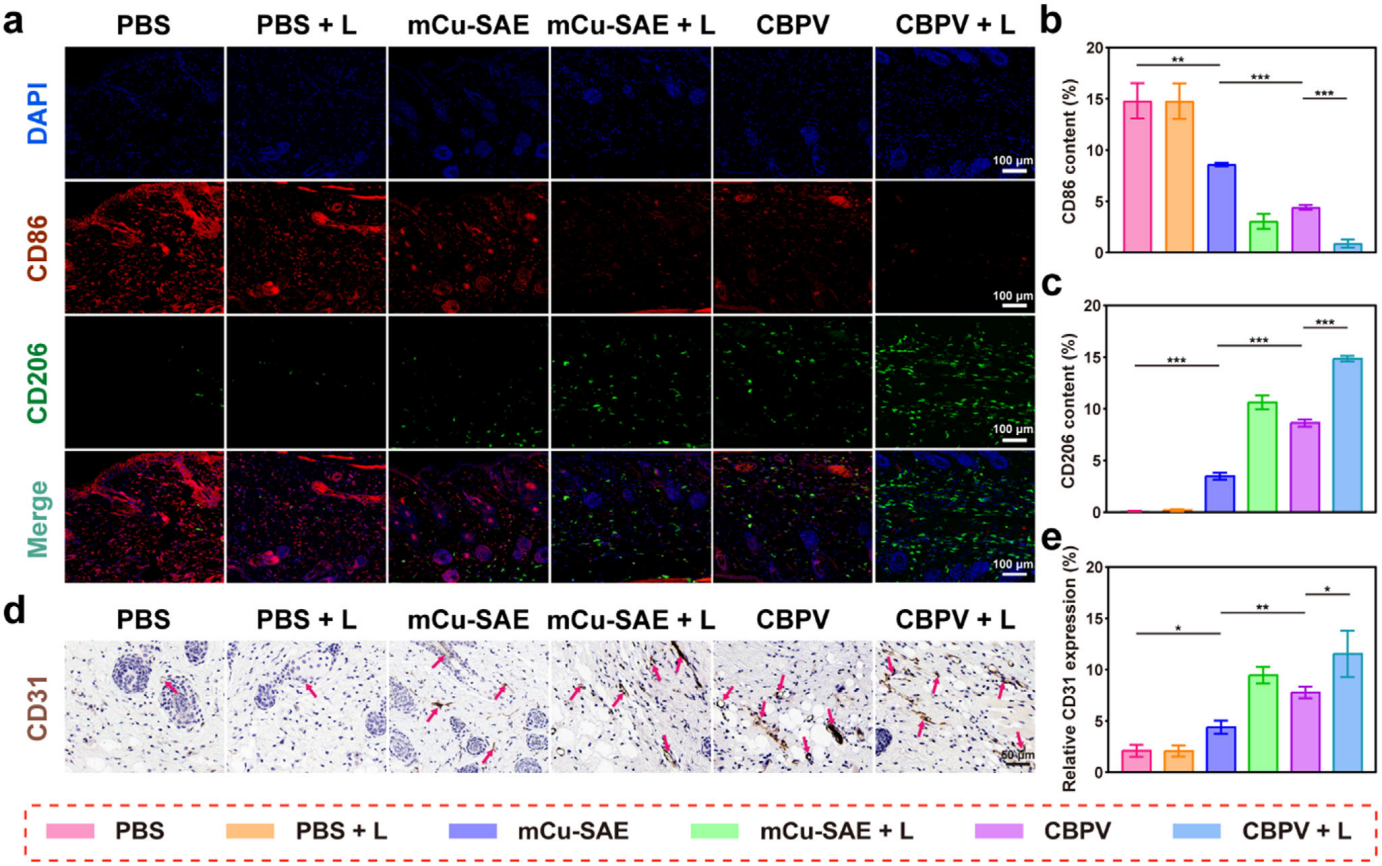
Immunofluorescence and immunohistochemical analysis of regenerated skin tissue following treatment in an epidermal diabetic wound model in mice. a) Immunofluorescence staining for CD86 and CD206 in regenerated skin at the wound site of mice from each group on day 7. Scale bar = 100 µm. b) Quantitative analysis of CD86 expression. c) Quantitative analysis of CD206 expression. d) Immunohistochemical analysis of CD31 expression on day 7. Scale bar = 50 µm. (Pink arrow: CD31‐positive endothelial cells). e) Quantitative analysis of CD31 in the epidermal wound model. Data are presented as mean ± SD (*n* = 3). *p < 0.05, **p < 0.01, ***p < 0.001.

To further elucidate the mechanism underlying the inflammatory changes in infected skin, we performed dual immunofluorescence staining of the epidermis using CD86 (red fluorescence) and CD206 (green fluorescence). Our findings revealed that the M1 macrophage marker protein CD86 decreased from 14.7% to 0.8% before and after treatment, while the M2 macrophage marker protein CD206 increased from 0.2% to 14.8% (**Figure** **8**a–c). M1 macrophages are responsible for producing pro‐inflammatory cytokines, whereas M2 macrophages secrete anti‐inflammatory cytokines. The observed shift from M1 to M2 macrophages may be the key factor driving the modulation of the inflammatory microenvironment in the skin. These results suggest that CBPV can facilitate the transition of M1 macrophages to M2 macrophages, thereby effectively alleviating inflammation in skin tissue following bacterial infection of epidermal wounds.

CD31 immunohistochemical staining was conducted on the wound skin tissue to evaluate inter‐group variations in angiogenesis. The control group (PBS and PBS + L) exhibited only a minimal number of platelet‐endothelial cell adhesion molecules (stained brown), indicative of limited angiogenic activity (Figure 8d,e). In contrast, the mCu‐SAE, mCu‐SAE + L, CBPV, and CBPV + L groups demonstrated a significant increase in blood vessel density. The CBPV + L group displayed the highest CD31 expression, reaching 11.5%, substantially higher than the 2.1% observed in the PBS group. This pronounced enhancement can be attributed to the synergistic bactericidal effects of ONOO^−^ and PTT and the promotion of angiogenesis by NO. The aforementioned histological analysis results indicate that CBPV, in conjunction with laser irradiation, can effectively eliminate bacteria at the wound site, thereby ameliorating the inflammatory response. Furthermore, these NPs promote collagen deposition in the epidermis and enhance angiogenesis at the wound site, thus facilitating the healing of diabetic epidermal wounds.

### 2.7 In Vivo Treatment of Subcutaneous Bacterial Infections

**Figure 9:**
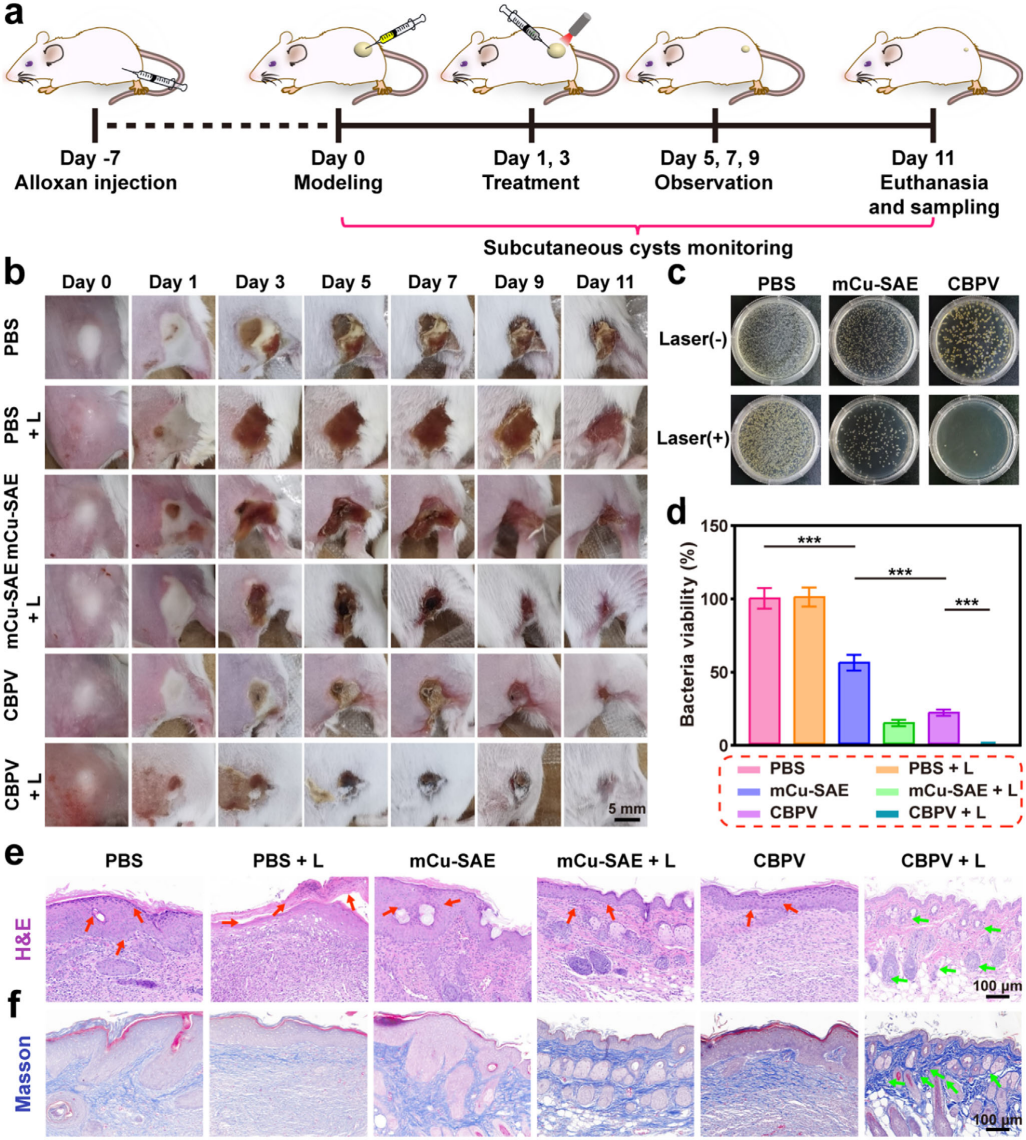
Assessment of the efficacy of CBPV in enhancing the healing of diabetic subcutaneous cysts. a) Establishment of a diabetic subcutaneous cyst model and schematic representation of the treatment protocol. b) Representative images of subcutaneous cysts in different groups of mice at days 0, 1, 3, 5, 7, 9, and 11. Scale bar = 5 mm. c) Photographs depicting colony growth in subcutaneous cysts from different groups on day 11. d) Quantitative analysis of colony growth in subcutaneous cysts from different groups on day 11. e, f) H&E and Masson's trichrome staining of regenerated skin on day 11. Scale bar = 100 µm. (Red arrow: inflammatory cells; Green arrow: hair follicles and glands). Data are presented as mean ± SD (*n* = 6). ***p < 0.001.

Subsequently, a subcutaneous cyst model was employed to evaluate the anti‐infection capability of CBPV in deep tissues. The entire treatment protocol is illustrated in **Figure** **9**a. Day 0 was designated as the day on which a mixture of MRSA and mineral oil was injected into the backs of the mice. The mice were then treated on days 1 and 3. During the treatment process, a thermal infrared imaging camera was utilized to monitor the temperature of the cyst area. After 10 min of laser irradiation, the PBS + L group, mCu‐SAE + L group, and CBPV + L group achieved temperatures of 42.2, 51.7, and 50.8 °C, respectively (Figure S35, Supporting Information). These results indicate that the 1064 nm laser exhibits excellent tissue penetration and can effectively induce PTT effects in subcutaneous tissues. Photographs of the subcutaneous cysts in the mice were taken on days 0, 1, 3, 5, 7, 9, and 11 (Figure 9b). The cyst images demonstrate a treatment trend consistent with that observed in the epidermal wounds, with the CBPV + L group exhibiting the most favorable therapeutic outcome. This finding suggests that the synergistic effect of highly toxic ONOO^−^ and PTT can effectively eradicate bacteria in subcutaneous tissues. On the eleventh day of treatment, bacteria from the subcutaneous cysts in the mice were collected and quantified using agar plates. As illustrated in Figure 9c,d, the mCu‐SAE group and the CBPV group achieved bacterial inhibition rates of 44.2% and 78.6%, respectively, due to the ROS they generated through nanozyme‐mediated biocatalysis. The addition of laser irradiation further enhanced the antibacterial effects. Compared to the mCu‐SAE group, the mCu‐SAE + L group exhibited a 41.5% increase in bacterial inhibition rate. Notably, the CBPV + L group demonstrated the lowest bacterial content, with a residual bacterial level of only 0.6%, indicating near‐total bacterial elimination.

Afterward, the histological analysis was performed on mice in the subcutaneous cyst model to evaluate the recovery of subcutaneous tissues treated with CBPV. Skin samples harvested on post‐treatment days 7 and 11 underwent H&E and Masson's trichrome staining to assess the anti‐infective efficacy of CBPV and the tissue regenerative capacity. Comparative histopathological analysis revealed a progressive restoration of subcutaneous architecture across sequential time points, demonstrating significant mitigation of infectious pathology and acceleration of tissue remodeling (Figure S36, Supporting Information). The H&E staining revealed that the mCu‐SAE group demonstrated improved inflammatory infiltration compared to the control group, with a trend of hair follicle regeneration. This improvement can be attributed to the bactericidal effect of ROS generated by mCu‐SAE. On the eleventh day in the CBPV + L group, the synergistic effects of PTT and ONOO^‐^ led to the near‐complete elimination of bacteria in the subcutaneous cysts. At this stage, inflammatory infiltration in the skin tissue was largely absent, and the hair follicles and glands were fully reconstituted (Figure 9e). Masson's trichrome staining results indicated that the skin tissue recovery in the treatment groups was superior to that in the control group. The CBPV + L group exhibited the highest collagen deposition, reaching 50.7%, which is significantly higher than the 37.6% in the CBPV group and the 41.4% in the mCu‐SAE + L group (Figure 9f; Figure S37, Supporting Information). These results demonstrate that CBPV, when assisted by lasers, are highly effective in eliminating subcutaneous bacteria and promoting the repair of skin tissue surrounding the cyst.

Next, an assessment of the inflammatory response was conducted for all groups to evaluate the changes of inflammation in the skin surrounding the cysts. First, the levels of pro‐inflammatory factors IL‐6 and TNF‐α were quantitatively assessed using immunohistochemistry. The treatment groups exhibited significantly lower levels of these pro‐inflammatory factors than the control group. Specifically, in the final group, the levels of IL‐6 and TNF‐α were reduced to merely 7.5% and 3.0%, respectively, which is consistent with the anti‐infective efficacy of CBPV in epidermal tissue (Figure S38, Supporting Information). This result suggests that CBPV exhibit superior deep‐seated anti‐infective and anti‐inflammatory properties under laser irradiation. The dual staining experiments for CD86 and CD206 also yielded similar results to those observed in the epidermal anti‐infection model. The content of CD86 decreased from 11.8% in the PBS group to 0.3% in the CBPV + L group, while the content of CD206 increased from 0.2% in the PBS group to 13.9% in the CBPV + L group. These findings indicate that CBPV can facilitate the transformation of M1 macrophages to M2 macrophages in subcutaneous tissues, thereby effectively mitigating inflammation in skin tissues following subcutaneous bacterial infection (Figure S39a–c, Supporting Information).

Additionally, we performed CD31 immunohistochemical staining on the skin surrounding the cysts to assess angiogenesis in the subcutaneous cyst area post‐treatment. In the control groups (PBS and PBS + L), angiogenesis was observed at a mere 2.1%, whereas in the CBPV + L group, the vascular increase reached 11.5%. This significant difference confirms that CBPV can effectively promote angiogenesis through multifunctional synergistic antibacterial activity and with the assistance of NO (Figure S39d,e, Supporting Information). The histological analysis results demonstrate that CBPV, when assisted by lasers, effectively eliminates subcutaneous bacteria and consequently improves the inflammatory response in wounds. Furthermore, they promote collagen deposition in the skin tissue surrounding the subcutaneous cysts and enhance angiogenesis at the cyst site.

Following the treatment, we assessed the in vivo biocompatibility of CBPV in both the epidermal wound and subcutaneous cyst models. The in vivo toxicity of CBPV was evaluated by monitoring changes in body weight. The results indicated that CBPV treatment had no significant effect on the body weight of mice (Figure S40, Supporting Information). Moreover, the major organs (heart, liver, spleen, lung, and kidney) from each group of mice were harvested after the treatment. H&E staining revealed no detectable lesions or inflammation in these organs (Figure S41, Supporting Information). Concurrently, blood samples were collected from the eyes of mice in both the PBS control group and the CBPV + L treatment group for routine hematological examinations and assessing liver and kidney functions. The test results indicated that the biochemical indicators in the treatment group were essentially consistent with those in the control group (Figure S42, Supporting Information). These findings demonstrate that CBPV exhibits excellent biocompatibility and safety in mice. Therefore, CBPV show remarkable biocompatibility in both epidermal wound and subcutaneous cyst models, laying a robust foundation for their biomedical applications.

### 2.8 In Vivo Therapeutic Mechanism via RNA Sequencing Analysis

**Figure 10:**
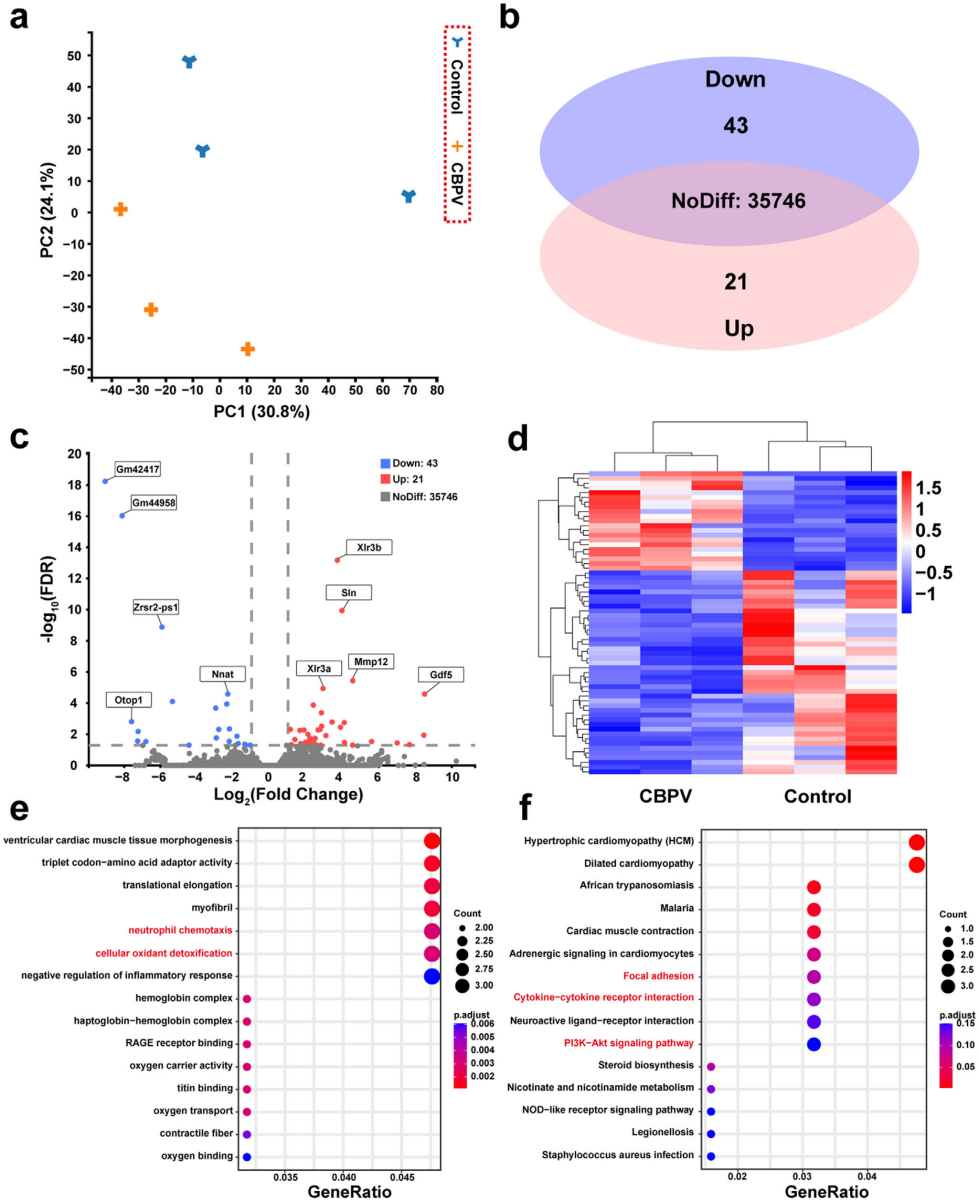
Analysis of RNA sequencing results for CBPV treatment. a) PCA analysis based on the differentially expressed genes in the skin tissues of the Control group and the CBPV + L group. b) Venn plots of up‐regulated and down‐regulated expressed genes in the control group and CBPV + L group. c) Volcano plot and d) heat map showing up‐ and down‐regulated genes. e) Significant enrichment of GO items for genes in two groups. f) KEGG pathway analysis of identified differentially expressed genes.

To identify genes or pathways potentially critical for wound healing, we performed transcriptomic analysis, utilizing in vivo RNA sequencing to elucidate the antibacterial mechanisms and wound‐healing promotion mediated by CBPV. Principal component analysis (PCA) distinguished the samples, revealing significant transcriptional differences between the CBPV + L group and the Control group (**Figure** **10**a). The Venn diagram and Volcano plot analysis identified 64 differentially expressed genes (DEGs) between the two groups, comprising 43 downregulated and 21 upregulated genes (Figure 10b,c). A heatmap depicts expression patterns for 64 DEGs between the Control and CBPV + L groups (Figure 10d). Gene Ontology (GO) enrichment analysis of these DEGs encompassed biological processes (BP), cellular components (CC), and molecular functions (MF). This GO analysis (Figure 10e) suggests that CBPV‐promoted wound healing may involve pathways such as neutrophil chemotaxis and cellular oxidant detoxification. Further Kyoto Encyclopedia of Genes and Genomes (KEGG) database analysis elucidated the impact of CBPV on signaling pathways (Figure 10f).^[^ ^52^ ^]^ Results indicate that Cytokine‐cytokine receptor interaction, Focal adhesion, and the PI3K‐Akt signaling pathway are significantly associated with the CBPV treatment group. These pathways directly drive the conversion of M1‐type macrophages into M2‐type macrophages, thereby effectively suppressing inflammation, promoting neovascularization, and contributing to the normalization of the pathological microenvironment.

## 3 Conclusion

In summary, we designed and fabricated a copper single‐atom nanozyme‐based therapeutic nanoplatform, CBPV, by strategically integrating multifunctional components to achieve spatiotemporally controlled antimicrobial and tissue‐regenerative effects. The hierarchically porous carbon matrix of CBPV, derived from Cu/DA/F127 self‐assembly and pyrolysis, achieves two significant breakthroughs: it maintains 100% atomic dispersion of Cu‐N_3_ catalytic sites and exhibits superior photothermal conversion efficiency (η = 43.9%) under NIR‐II irradiation. This nanoarchitecture, featuring coordinatively unsaturated active copper sites, facilitates potent dual‐enzyme mimetic activity (POD and OXD), which drives the simultaneous generation of ROS, NO, and RNS through thermally amplified cascade reactions. By synergizing Van‐mediated targeting with NIR‐II‐responsive PTT, CDT, and NO therapy, the CBPV platform demonstrates complete biofilm eradication via ONOO^−^/hyperthermia dual attack mechanism, accelerated diabetic wound closure by modulating the inflammatory microenvironment and promoting NO‐mediated angiogenesis, and deep‐tissue sterilization capability enabled by superior NIR‐II laser penetration depth. The combination of multi‐modal sterilization methods in the CBPV nanoplatform effectively overcomes the limitations of traditional single‐modal antimicrobial methods. Significantly, the demonstrated spatiotemporal control of biocatalysis, photothermal, and gas therapeutics provides a blueprint for developing intelligent nanozyme‐based systems in precision medicine. Overall, this work not only advances the fundamental understanding of multi‐enzyme mimicking nanomaterials but also establishes a versatile therapeutic platform for managing complex infections, with immediate implications for diabetic wound care and deep‐seated biofilm eradication.

## 4 Experimental Section

Experimental details, including materials, instruments, photothermal performance, and photothermal stability experiments, bacterial culture, bacterial ROS level detection, biocompatibility assessment of nanomaterials, and statistical analysis, are listed in the Supporting Information.

### Preparation of CBPV

First, 1.0 g of Pluronic F127 and 0.5 g of DA were added to a 100 mL mixture of water and ethanol (water: ethanol = 1: 1) under ultrasound to ensure thorough dissolution. Then, 1.3 mg (0.005 mmol) of Cu(C_5_H_7_O_2_)_2_ was added and dissolved completely. Under stirring conditions, 2 mL of MES was slowly added to the solution, and the reaction took 30 min to obtain an emulsion. Next, 5 mL of NH_3_·H_2_O was added slowly, and the mixture was stirred for 60 min. The resultant product, Cu/PDA/F127, was collected by centrifugation at 10 000 rpm for 10 min and washed three times with anhydrous ethanol. The product was then placed in a vacuum‐drying oven and heated at 60 °C overnight. Following this, the dried Cu/PDA/F127 was transferred to a tubular furnace and heated under nitrogen flow at 350 °C for 3 h with a heating rate of 1 °C per min, followed by calcination at 800 °C for 2 h with the same heating rate. After cooling, the calcinated sample was added to the hydrochloric acid (0.5 M), and the mixture was stirred in an oil bath at 60 °C for 24 h to obtain mCu‐SAE after filtration.

To prepare the CBPV, Van (30 mg, 0.02 mmol), EDC (80 mg, 0.4 mmol), and NHS (60 mg, 0.5 mmol) were dissolved in 1 mL of ultrapure water. The mixture was allowed to activate for 1 h. Subsequently, MPEG5000‐NH_2_ (60 mg, 0.01 mmol) was introduced, and the mixture was stirred for 6 h. Afterward, the mixture underwent dialysis for 3 days to purify PEG‐Van. BNN6 was subsequently loaded into mCu‐SAE via the anti‐solvent crystallization method. Specifically, 10 mg BNN6 was dissolved in 0.5 mL ethanol, followed by a dropwise addition of 10 mL mCu‐SAE (0.5 mg mL^−1^) aqueous solution under continuous stirring. The mixture was equilibrated for 12 h to facilitate efficient drug loading through controlled crystalline deposition. The synthesized PEG‐Van was then added to the mixture and stirred overnight. Excess reactants were removed by centrifugation at 10 000 rpm for 10 min. The product was then washed three times with ethanol and ultrapure water to yield pure CBPV.

### POD‐Like Activity Experiment

this study utilized MB and TMB to evaluate the •OH generation capacity of NPs. For the TMB assay, solutions with a gradient of NP concentrations (0, 25, 50, 100, and 200 µg mL^−1^) were prepared. Hydrogen peroxide (H_2_O_2_) at a concentration of 1 mMM and TMB at 0.02 M were added to these solutions, and the mixtures were incubated for a specified period. The changes in absorbance were measured using a UV–vis spectrophotometer. Additionally, the effect of different concentrations of H_2_O_2_ (0, 0.5, 1, 2, and 5 mm) on the generation of •OH was assessed using the same method. To further validate the photothermal promotion of •OH production, TMB colorimetric reactions were conducted both with and without laser irradiation. For the MB experiment, solutions with a similar concentration gradient of NPs (0, 25, 50, 100, and 200 µg mL^−1^) were prepared. H_2_O_2_ at 1 mM and MB at 15.6 µM were added to these solutions, followed by a brief incubation period. The changes in absorbance were again detected using a UV–vis spectrophotometer.

### Steady‐State Enzyme Kinetics Studies

The Michaelis–Menten constant (K_m_) and maximum velocity (V_max_) were determined based on the Michaelis–Menten saturation curve.

### OXD‐Like Activity Experiment

This study used BQ and a superoxide anion detection kit to measure •O_2_^−^ production. Two identical test tubes were prepared, each containing CBPV, H_2_O_2_, and MB. BQ was added to one of the test tubes, and both tubes were then incubated at 37 °C for a specified period. The changes in absorbance for both tubes were subsequently measured using a UV–vis spectrophotometer. Additionally, the •O_2_^−^ detection kit was employed to quantify •O_2_^−^ production. A series of samples with varying concentrations of CBPV (12.5, 25, 50, 100, and 200 µg mL^−1^) were prepared. The experimental protocol provided by the kit was followed, and the absorbance of each sample was measured at 530 nm using a UV–vis spectrophotometer. The •O_2_^−^ content for each concentration was determined by using a standard curve generated from standard solutions.

### In vitro NO Release

CBPV were dispersed in deionized water at various concentrations (25, 50, 100, 150, and 200 µg mL^−1^). The samples were then irradiated with a NIR laser (1064 nm, 1.25 W cm^−2^) for 10 min, followed by centrifugation at 10,000 rpm for 10 min. Subsequently, 50 µL of the supernatant was collected, and the NO release under laser irradiation for each concentration was detected using the Griess assay kit. Similarly, the effects of various incubation intervals (0, 10, 20, 40, 60, 120, 240, and 360 min) at 37 °C and different laser irradiation durations (0, 1, 3, 5, 7, and 10 min) on NO release were evaluated. Additionally, the impact of different laser power settings (0.5, 0.75, 1, 1.25, 1.5, 1.75, and 2 W cm^−2^) on NO release was assessed, as well as the changes in NO release at room temperature over varying incubation intervals (0, 1, 3, 5, 7, and 10 min).

Additionally, the DAF‐FM DA fluorescent probe was employed to investigate the NO release properties. DAF‐FM DA (5 µL, 5 µM) was mixed with varying concentrations of CBPV (0, 25, 50, 100, 150, and 200 µg mL^−1^), and NO production was analyzed using a fluorescence spectrophotometer after laser irradiation for a consistent duration.

### In Vitro ONOO^−^ Detection

Dihydrorhodamine 123 (DHR123) serves as a fluorescent probe exhibiting preferential oxidation kinetics toward ONOO^−^ over other ROS, generating rhodamine 123 with significantly enhanced quantum yield. For quantitative analysis, DHR123 (5 µM) was reacted with test materials and H_2_O_2_ (100 µL, 1 mM) at 37 °C for 60 min, followed by spectrofluorometric quantification. Complementarily, spatiotemporal ONOO^−^ generation during bactericidal activity was monitored by co‐incubating DHR123 with H_2_O_2_, nanomaterials, and bacteria for a specified period before the confocal imaging using a Leica STELLARIS 8 STED system with 63 × oil immersion objective.

### Bacterial Capture

The fluorescent dye Rhodamine B (RhB) was used to label various NPs. By stirring RhB (5 mg) with 5 mL of the mCu‐SAE (1 mg mL^−1^), CB@PEG (1 mg mL^−1^), or CBPV (1 mg mL^−1^) aqueous solution for 12 h and then washing with deionized water until the supernatant is colorless, NPs with fluorescent emission capabilities are obtained. The RhB‐labeled NPs (1 mg mL^−1^, 200 µL) were then added to 100 µL of a bacterial suspension and 700 µL of PBS in a 1 mL centrifuge tube and incubated at 37 °C for 2 h. After incubation, the mixture was centrifuged at 8,000 rpm for 5 min, and the supernatant was removed. The pellet was treated with 400 µL of DAPI and incubated at room temperature for 2 h. Following this, the sample was washed three times with PBS. Finally, the pellet was resuspended in 20 µL of PBS, and 10 µL of the resuspended sample was placed on a microscope slide for observation using a Leica‐CS‐SP8‐STED LSCM.

### In vitro Antimicrobial Activity Evaluation

The in vitro antibacterial activity of CBPV was evaluated using the agar plate method. *E. coli* or MRSA (1 mL, 1 × 10⁷ CFU mL^−1^) cell suspension and NPs (1 mL, 50 µg mL^−1^) were mixed. The experiment was conducted using twelve different treatment methods, as follows: 1) PBS treatment alone, 2) 1064 nm laser irradiation (1.25 W cm^−2^) of PBS‐treated bacteria, 3) 250 µM H_2_O_2_ treatment, 4) 1064 nm laser irradiation (1.25 W cm^−2^) of H_2_O_2_‐treated bacteria, 5) co‐treatment with 50 µg mL^−1^ mCu‐SAE and 250 µM H_2_O_2_, 6) 1064 nm laser irradiation (1.25 W cm^−2^) of mCu‐SAE and H_2_O_2_ co‐treated bacteria, 7) co‐treatment with 50 µg mL^−1^ NMCNPs@BNN6 NPs and 250 µM H_2_O_2_, 8) 1064 nm laser irradiation (1.25 W cm^−2^) of NMCNPs@BNN6 NPs and H_2_O_2_ co‐treated bacteria, 9) co‐treatment with 50 µg mL^−1^ CB NPs and 250 µM H_2_O_2_, 10) 1064 nm laser irradiation (1.25 W cm^−2^) of CB NPs and H_2_O_2_ co‐treated bacteria, 11) co‐treatment with 50 µg mL^−1^ CBPV and 250 µM H_2_O_2_, and 12) 1064 nm laser irradiation (1.25 W cm^−2^) of CBPV and H_2_O_2_ co‐treated bacteria. After treatment, each group was incubated at 37 °C for 2 h. Subsequently, the cell suspensions were diluted with PBS, and 40 µL of the diluted suspension was evenly spread on solid LB agar plates. The plates were inverted, sealed, and incubated at 37 °C overnight. The following day, the agar plates were photographed, and colony counts were recorded.

### Live/Dead Bacterial Staining

After grouping and treating the samples using the agar plate method, they were incubated at 37 °C for 2 h. The samples were then centrifuged at 8,000 rpm for 5 min, and the supernatant was discarded. Following this, 10 µL of SYTO‐9 and 10 µL of PI were added, and the mixture was incubated at 37 °C for 30 min. SYTO‐9 stains both live and dead bacteria green, while PI specifically stains only dead bacteria red. The samples were washed three times with PBS, and 10 µL of the stained bacterial suspension was transferred onto a glass slide. Finally, the staining was observed using LSCM to assess the antibacterial activity of the different functions of CBPV.

### 3D Biofilm Removal Experiment

One milliliter of *E. coli* or MRSA (1 × 10^8^ CFU mL^−1^) was added to the confocal dish and incubated at 37 °C for 48 h, with the LB liquid medium being changed every 12 h. The biofilm was then washed three times with sterile PBS. Following this, the samples were grouped and treated according to the agar plate method and incubated at 37 °C for 2 h. The live and dead bacteria on the biofilm were stained using SYTO‐9 and PI. After a 30‐min incubation at 37 °C, the excess dye was washed away with sterile PBS. Finally, LSCM was used to observe the extent of biofilm disruption in each group.

### Bacterial Morphological Analysis

First, E. coli or MRSA was diluted in a liquid LB medium to achieve the appropriate concentration. The samples were grouped and treated using the agar plate method, then incubated at 37 °C for 2 h. Next, 500 µL of the treated bacterial suspension was added to poly‐L‐lysine‐coated cell coverslips. After incubating at 37 °C for 12 h, the supernatant was removed. The bacteria were then fixed by adding 200 µL of 2.5% glutaraldehyde and incubating for at least 6 h. Following fixation, gradient dehydration was carried out using ethanol at various concentrations. Finally, SEM was used to observe the bacterial morphology in each group.

### Cell Scratch Assay

We investigated the impact of CBPV on the migratory behavior of vascular cells using in vitro scratch assays and Transwell migration assays. Initially, a cell scratch assay was performed. L929 cells were cultured in 12‐well plates until they reached 90% confluence. Subsequently, a cell‐free wound was generated in each well using a 200 µL sterile pipette tip. Following multiple washes with PBS, mCu‐SAE, CB, and CBPV (1 mL, 25 µg mL^−1^) were added to the respective groups. Cell migration was subsequently monitored at predetermined time intervals using an inverted fluorescence microscope.

To further assess the impact of NO released from CBPV on the migratory behavior of vascular cells, Transwell migration assays were conducted. HUVECs (1 × 10^4^ cells well^−1^) were suspended in 100 µL of DMEM medium containing 25 µg mL^−1^ NPs and 1% FBS. Subsequently, the cells were seeded in the upper chamber of the Transwell, while DMEM (1 mL, containing 20% FBS) was added to the lower chamber. Following a 24‐h incubation at 37 °C and 5% CO_2_, non‐migrated cells remaining on the upper layer were gently removed with a cotton swab. The cells that migrated to the lower surface of the Transwell were then stained with a 1% crystal violet solution and visualized using an inverted fluorescence microscope.

### Tube Formation Analysis

Matrigel was coated onto 12‐well plates and allowed to solidify for 30 min at 37 °C. Subsequently, HUVECs (2 × 10^4^ cells well^−1^) were seeded in each well and cultured in serum‐free DMEM supplemented with 25 µg mL^−1^ NPs. After incubation for 6 h in an incubator at 37 °C and 5% CO_2_, the cells were imaged using an inverted fluorescence microscope.

### Animal Ethics Statement

All animal experiments were carried out with the permission of the Institutional Animal Protection and Utilization Committee of Wuhan University in China (Approval Number: WQ20210366). All procedures followed the animal ethical standards.

### Diabetic Mouse Model

A type 1 diabetes mouse model was established using 4‐5‐week‐old, 18–20 g female Kunming mice (Henan Skobes Biotechnology Co., Ltd., China). First, the mice, which had been fasted overnight, were injected with alloxan (10 mg mL^−1^) at a dosage of 150 mg kg^−1^ for three consecutive days. After the three‐day injection period, blood glucose levels were measured. Successful establishment of the type 1 diabetes mouse model is indicated by fasting blood glucose levels >11 mmol L^−1^ within one week.

### Epidermal Wound Infection Model

An epidermal wound infection model was established in mice with successfully induced diabetes. A full‐thickness circular wound with a diameter of 8 mm was created on the back of each mouse. Following this, 100 µL of MRSA (1 × 10^7^ CFU mL^−1^) was inoculated onto the wound to establish the epidermal wound infection model, with day 0 designated as the establishment day. The diabetic mice were randomly divided into six groups (*n* = 10): Group 1 (PBS treatment), Group 2 (PBS + laser treatment), Group 3 (mCu‐SAE treatment), Group 4 (mCu‐SAE + laser treatment), Group 5 (CBPV treatment), and Group 6 (CBPV + laser treatment). Treatments were administered on days 1 and 3, and wound temperature changes were recorded using a photothermal imaging system. Mouse body weight and infection wound status were monitored on days 1, 3, 7, 9, and 13. On day 3 after treatment, exudates from the wounds in each group were plated to observe bacterial growth at the wound sites. Epidermal tissue samples were collected on days 3, 7, and 13 for H&E staining, Masson staining, immunohistochemistry, and immunofluorescence experiments to evaluate wound healing. To assess the toxicity of the treatments, major organs (heart, liver, spleen, lungs, and kidneys) along with whole blood and serum were collected on day 13 for H&E staining, complete blood count, and liver and kidney function tests.

### Subcutaneous Cyst Infection Model

A subcutaneous cyst infection model was established in mice with successfully induced diabetes. The diabetic mice were randomly divided into six groups (*n* = 10): Group 1 (PBS treatment), Group 2 (PBS + laser treatment), Group 3 (mCu‐SAE treatment), Group 4 (mCu‐SAE + laser treatment), Group 5 (CBPV treatment), and Group 6 (CBPV + laser treatment). A 100 µL mixture of bacteria and mineral oil (1:1) was injected into the backs of the mice in each group to establish the subcutaneous cyst infection model, with day 0 designated as the establishment day. Treatments were administered on days 1 and 3, and changes in cyst temperature were recorded using a photothermal imaging system. Mouse body weight and changes in subcutaneous cyst size were monitored on days 0, 1, 3, 5, 7, 9, and 11. On day 11, bacteria from the cyst site were plated to observe the residual bacterial count. On days 7 and 11, skin tissue surrounding the cyst was collected for H&E staining, Masson staining, immunohistochemistry, and immunofluorescence assays to evaluate the skin recovery around the cyst. On the final day, the main organs (heart, liver, spleen, lungs, and kidneys), as well as whole blood and serum, were collected. H&E staining, complete blood count, and liver and kidney function tests were performed to evaluate the biosafety of the entire treatment process.

### Statistical Analysis

All data are expressed as the mean ± standard deviation (mean ± SD) and were analyzed using GraphPad Prism 8 software. Each experiment was performed with at least three replicates, and t‐tests were used to determine statistical differences between groups. A *p* < 0.05 is considered indicative of a significant difference, with significance levels denoted as *p < 0.05, **p < 0.01, and ***p < 0.001.

## Conflict of Interest

The authors declare no conflict of interest.

## Supporting information



Supporting Information

## Data Availability

The data that support the findings of this study are available from the corresponding author upon reasonable request.
