# Supplementary material for: Bacteria‐Targeted Single‐Atom Nanozyme With Photothermal‐Augmented Multi‐Enzymatic Cascade and NO Delivery for Enhanced Infected Wound Healing
Source: Adv Sci (Weinh). 2025 Aug 14;12(42):e09621. doi: 10.1002/advs.202509621 (PMC12622438; doi:10.1002/advs.202509621)
Supplement: Supplementary file 1 — Supporting Information [file ADVS-12-e09621-s001.docx]

Supporting Information

Bacteria-Targeted Single-Atom Nanozyme with Photothermal-Augmented Multi-Enzymatic Cascade and NO Delivery for Enhanced Infected Wound Healing

Junyang Chen, Qing Chen, Xudong Qin, Haixia Yang, Xin Wang, Jianliang Zhou*, Ying-Wei Yang*, Jian Tian*

J. Chen, Q. Chen, X. Qin, H. Yang, Prof. Dr. J. Zhou, Prof. Dr. J. Tian

Department of Cardiovascular Surgery, Zhongnan Hospital of Wuhan University, School of Pharmaceutical Sciences, Wuhan University, Wuhan 430071, P. R. China

*E-mail: zjl20210802@whu.edu.cn (J.Z.); jian.tian@whu.edu.cn (J.T.)

Prof. Dr. X. Wang, Prof. Dr. Y.-W. Yang

College of Chemistry, Jilin University, 2699 Qianjin Street, Changchun 130012, P. R. China

*E-mail: ywyang@jlu.edu.cn (Y.-W.Y.)

Prof. Dr. X. Wang, Prof. Dr. Y.-W. Yang

Department of Radiation Oncology, China-Japan Union Hospital of Jilin University, Changchun 130033, P. R. China

**Supporting Experimental Section**

**1.** **Materials**

Pluronic F-127 (F127), Dopamine hydrochloride (DA), 1,3,5-Trimethylbenzene (MES), Ammonium hydroxide solution (NH₃·H₂O, 28%), Copper(II) acetylacetonate (Cu(C_5_H_7_O_2_)_2_), Vancomycin (Van), Mlneral oll, (3-(4,5-Dimethyl-thiazolyl)-2,5-dimethylthiazol-2-yl)-2,5-diphenyltetrazolium bromide (MTT), Methylene blue (MB) and 3,3',5,5'-tetramethylbenzidine dihydrochloride (TMB) were all purchased from Aladdin (China). MPEG_5000_-NH_2_ was bought from Shang Hai Ponsure Biotech, Inc. (China). N-Hydroxysuccinimide (NHS) was provided by Energy Chemicals (China). 1,4-Benzoquinone (BQ) was bought from RHAWN Chemical Agents (China). Hydrochloric acid (HCl), ethanol absolute, and dimethyl sulfoxide (DMSO) were all bought from Sinopharm Chemical Reagent Co., Ltd. (China). N, N′-di-sec-butyl-N, N′-dinitroso-1,4-phenylenediamine (BNN6) was bought from Xi'an Ruixi Biological Technology Co., Ltd. (China). N-(3-dimethylaminopropyl)-n-ethylcarbodiimide hydrochloride (EDC) was purchased from Macklin (China). 2′,7′-Dichlorofluorescein diacetate (DCFH-DA), Calcein-AM/PI Double Stain Kit, NO Assay Kit, and 4',6-diamidino-2-phenylindole (DAPI) were all purchased from the Beyotime Institute of Biotechnology (China). Superoxide Anion Activity Content Assay Kit was bought from Solarbio (China). SYTO-9 Green Fluorescent Nucleic Acid Stain was bought from Thermo Scientific (USA). Alloxan was provided by Shanghai Yuanye Bio-Technology Co. Ltd. (China). Dulbecco's modified Eagle's medium (DMEM) and fetal bovine serum were bought from Gibco Life Technologies (USA). All materials were purchased from reagent vendors and used without further purification.

**2. Instruments**

The UV-vis spectra of CBPV were measured through the Shimadzu UV-vis spectrophotometer (UV-2600, Japan). The FT-IR spectra were measured through the Fourier transform infrared spectrometer (THERONIO, USA). Confocal laser fluorescence microscope (CLSM) images were obtained from the Confocal laser fluorescence microscope (TCS SP8, German). The TEM experiment was conducted using the Field Emission Transmission Electron Microscope (JEM-F200, Japan). The SEM experiment was performed using the Field Emission Scanning Electron Microscope (Zeiss GeminiSEM 500, United Kingdom). The NIR-II absorption of the NPs was measured by the UV-VIS-NIR Spectrometer (UH5700, Japan). The hydrodynamic particle size and zeta potential of the NPs were analyzed using the Malvern Zetasizer Nano series ZS-90. The HRTEM images, the EDS elemental mapping analysis, the HAADF-STEM images, and the SAED images were all measured through the Double-spherical aberration corrected field emission transmission electron microscopy (JEM-ARM200CF, Japan). SAzymes were synthesized using a tube furnace (OTF-1200X-S, China).

**3. BNN6 drug loading assay**

BNN6 standard solutions were prepared at a series of concentrations (5, 10, 20, 40, and 80 μg mL^-1^). The absorbance was measured at 260 nm to generate a BNN6 concentration-absorbance standard curve. Drug loading capacity (LC) of BNN6 was determined by measuring the residual amount of BNN6 in the supernatant after loading, and the formula used is as follows:

$LC\%=\frac{m_{0}-m_{1}}{m_{0}-m_{0}+m}$ (S1)

Where, m_0_ is the feeding amount of BNN6; m_1_ is the mass of BNN6 in the supernatant; m is the mass of mCu-SAE.

**4. Surface loading of PEG-Van**

PEG-Van standard solutions were prepared at a series of concentrations (0.25, 0.3, 0.5, 0.6, and 1 μg mL^-1^). The absorbance was measured at 280 nm to generate a PEG-Van concentration-absorbance standard curve. LC of PEG-Van was determined by measuring the residual amount of PEG-Van in the supernatant after loading. The calculation formula is given by **Equation (S1)**.

**5. Release of Cu ions**

The released Cu ions were detected using the dialysis method. CBPV (1 mL, 2.1 mg mL^-1^) and H_2_O_2_ (10 μL, 100 mM) were added to the dialysis bag, with 35 mL of PBS added outside the dialysis bag. Samples were collected from the centrifuge tube at different time points (0, 2, 15, 30, 120, and 1440 min) for ICP-MS analysis.

**6. Photothermal performance and photothermal stability experiments**

CBPV were dispersed in water at various concentrations (12.5, 25, 50, 100, and 200 μg mL^-1^). The samples were then irradiated with a NIR laser (1064 nm, 1.25 W cm^-2^) for 10 min, using water as the control. Additionally, NPs at a concentration of 200 μg mL^-1^ were irradiated with 1064 nm lasers at different power densities (0.5, 0.75, 1, 1.25, 1.5, 1.75, and 2 W cm^-2^). The photothermal stability of the NPs was evaluated by subjecting them to five heating and cooling cycles. Temperature changes during the photothermal heating and natural cooling processes were recorded for both the NPs and the control group. The photothermal conversion efficiency (PCE) of the NPs is determined using **Equation (S2)**, as follows:

$x=\frac{hS(T_{\max}-T_{\mathrm{sur}})-Q_{\mathrm{dis}}}{I(1-{10}^{-A1064})}$ (S2)

Where, h is the heat transfer coefficient, S is the surface area of the container, T_max_ is the maximum temperature during the heating process, T_surr_ is the initial temperature before laser irradiation, Q_dis_ is the heat input from the laser to the container, I is the laser power, A_1064_ is the absorbance of the sample at 1064 nm, and the value of hS can be calculated using **Equation (S3)** as follows:

$\mathrm{hS}=\frac{M_{D}C_{D}}{\tau}$ (S3)

Where, M_D_ is the mass of the first-level water, C_D_ is the specific heat capacity of the first-level water, and τ is the time constant, which can be calculated using **Equation (S4)** and **Equation (S5)** as follows:

$\tau=\frac{t}{-ln\theta}$ (S4)

$\theta=\frac{T-T_{\mathrm{sur}}}{T_{\max}-T_{\mathrm{sur}}}$ (S5)

The Q_dis_ in (1) can be calculated using **Equation (S6)** as follows:

$Q_{\mathrm{dis}}=hS(T_{max(H_{2}O)}-T_{\mathrm{sur}})$ (S6)

**7.** **Bacterial culture**

*E. coli* (Gram-negative) and MRSA (Gram-positive) were used as the experimental strains. The bacteria were cultured overnight in Luria-Bertani (LB) medium (15 mL).

**8. Bacterial ROS level detection**

After treating the bacteria according to the agar plate method, the samples were incubated at 37 °C for 2 h. Next, 10 μL of a 10 mM DCFH-DA probe was added, and the samples were incubated in a 37 °C incubator for 30 min. Finally, the samples were washed three times with sterile PBS, placed on slides, and observed using LSCM to assess the ROS levels in each group of bacteria.

**9. Hemolysis assay**

First, blood samples were collected from the ocular region of the mice. The whole blood was then centrifuged at 3000 rpm for 5 min, and the red blood cells were washed with PBS five times until the supernatant was colorless. Next, solutions with concentration gradients (25, 50, 100, 200, and 400 μg mL^-1^) were prepared and incubated with 10 μL of red blood cells at 37 °C for 2 h. PBS was used as the negative control, and distilled water was used as the positive control. The incubated mixtures were centrifuged at 8000 rpm for 5 min, and 100 μL of the supernatant was transferred to a 96-well plate. The absorbance at 450 nm was measured using a microplate reader. Finally, the hemolysis rate was calculated using **Equation (S7)** as follows: $Hemolysis rate (\%)=\frac{I-I_{0}}{I_{H_{2}O}-I_{0}}\times100 \%$ (S7)

where I is the absorbance of the sample group supernatant, *I_0_* is the absorbance of the negative control group supernatant, and I*_H2O_* is the absorbance of the positive control group supernatant.

**10. Cytotoxicity assay**

This study assessed the *in vitro* cytotoxicity of CBPV using the MTT assay. L929 or 4T1 cells were added to each well of a 96-well plate in a volume of 100 μL. The plate was then incubated in a cell culture incubator for 24 h. After incubation, the supernatant was discarded, and 100 μL of CBPV at different concentrations (0, 12.5, 25, 50, 100, 200, and 400 μg mL^-1^) was added to each well. The plate was incubated again in the cell culture incubator for 24 h. Subsequently, the supernatant was aspirated, and 100 μL of MTT solution (0.5 mg mL^-1^) was dispensed into each well following three sequential washes with PBS. The plate was then incubated in the cell culture incubator for 4 h. The medium was carefully removed, and 100 μL of DMSO was added to each well. After a 5-minute reaction, the absorbance at 491 nm was measured using a microplate reader.

**Supporting Figures**


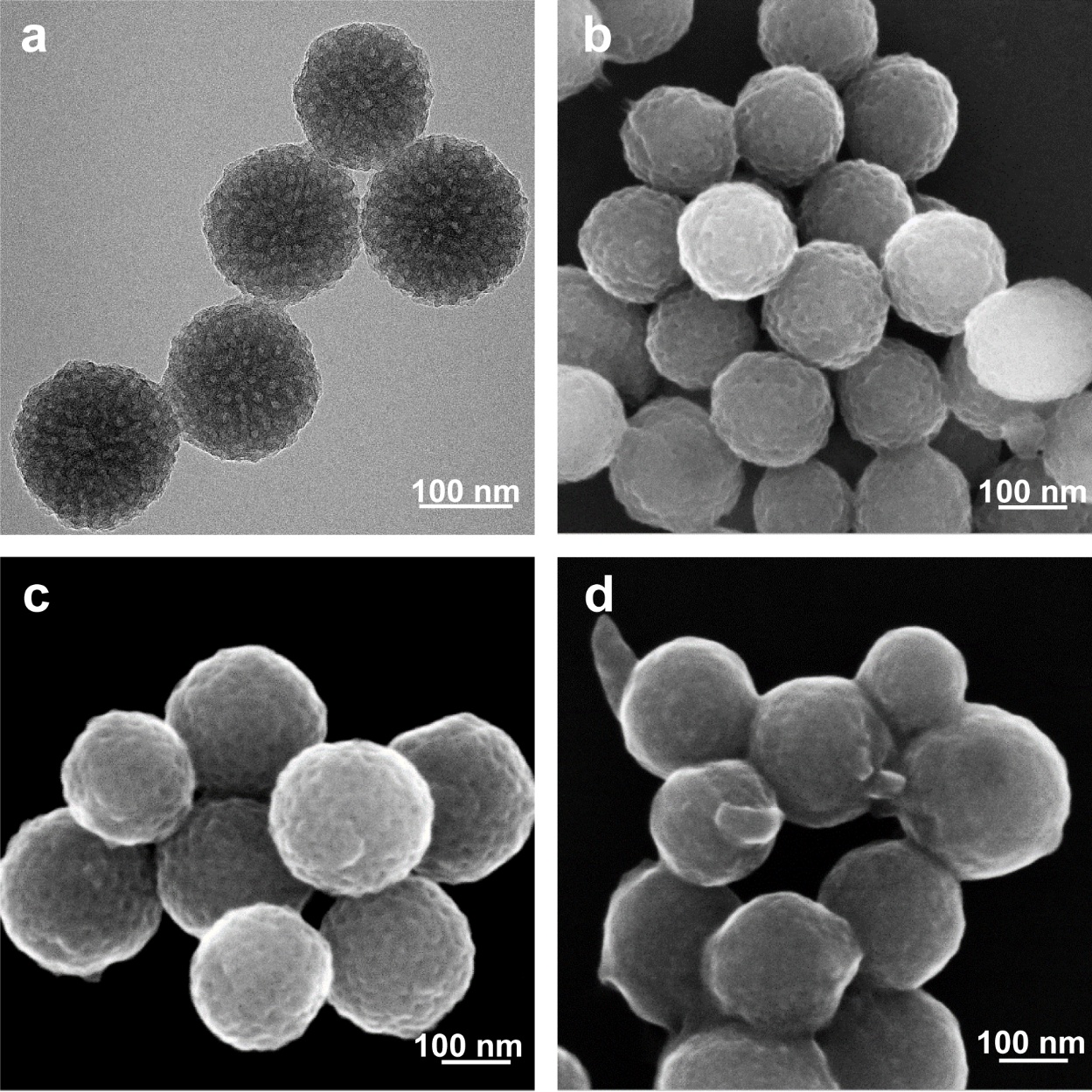


**Figure S1.** a) TEM images of Cu/PDA/F127. SEM images of b) Cu/PDA/F127, c) mCu-SAE, and d) CBPV. Scale bar = 100 nm.


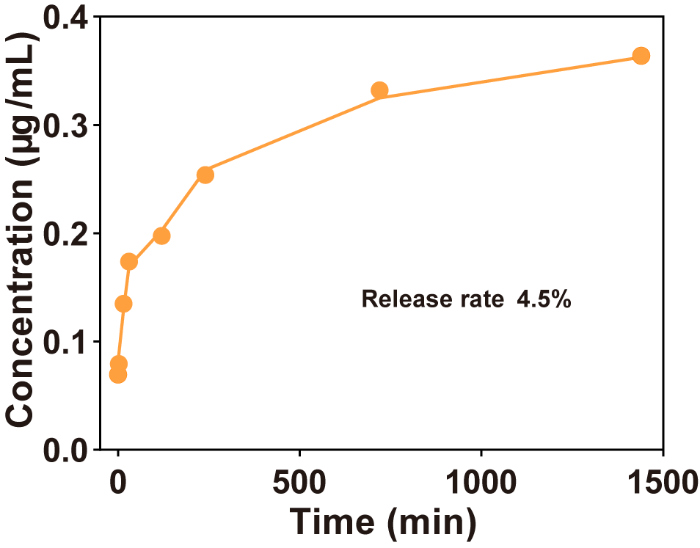


**Figure S2.**  The release curve of Cu from mCu-SAE after incubated in H_2_O_2_ (1 mM) for 24 hours.


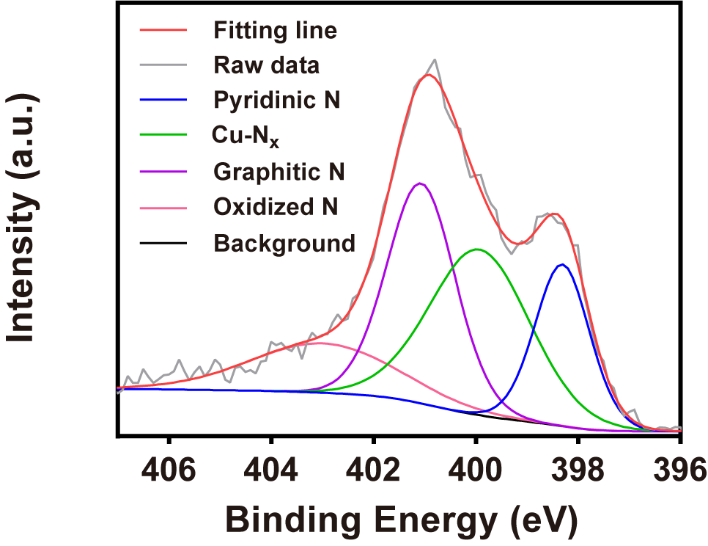


**Figure S3.** High-resolution spectrum of N 1s of mCu-SAE. In line with the literature report^[1]^, the metal-N_x_ peak is observed at approximately 399.8 eV, while the pyrrole N peak is not detected.


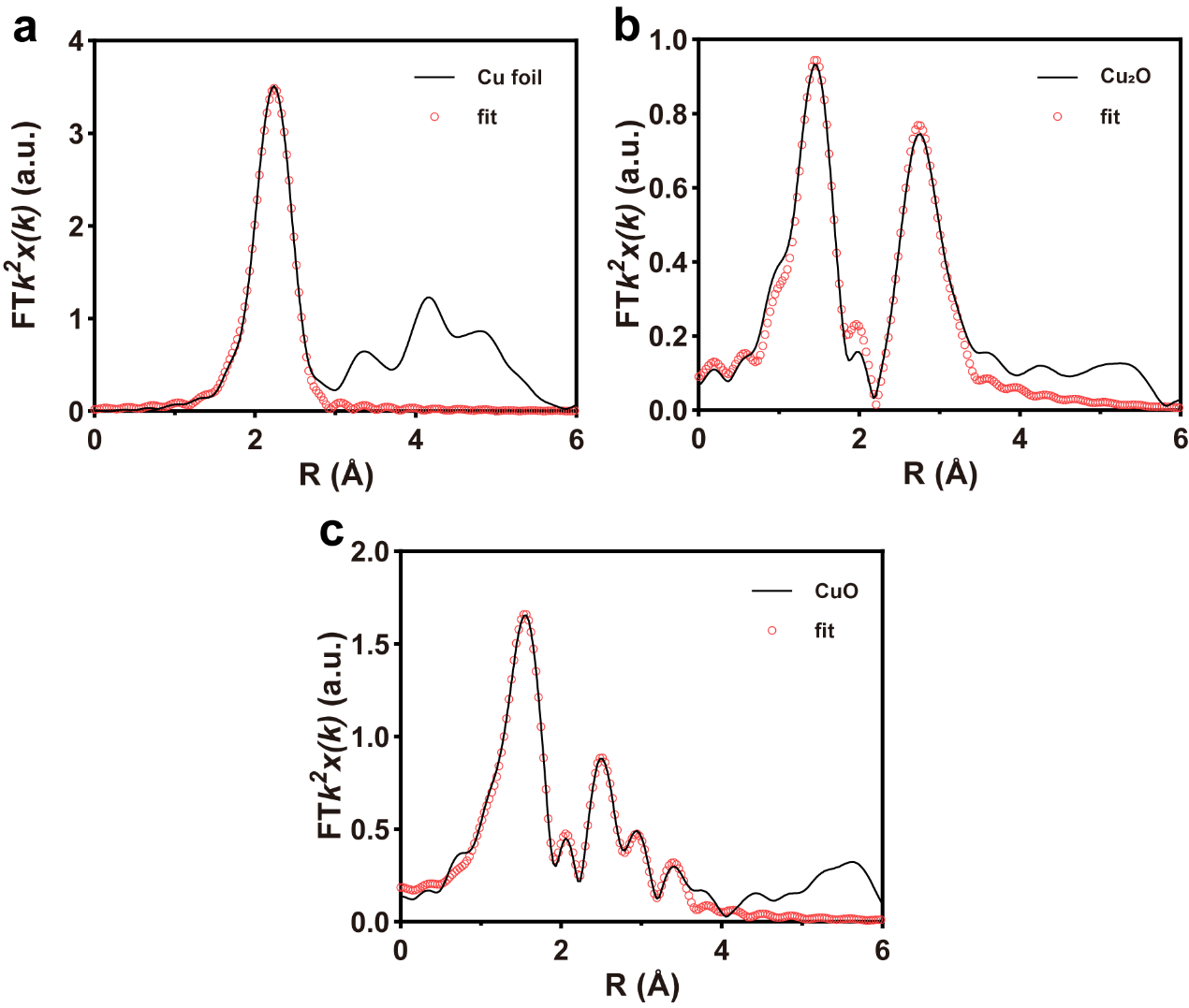


**Figure S4.** a) FT-EXAFS fitting curves at R space of Cu K-edge for Cu foil, b) Cu_2_O, and c) CuO.


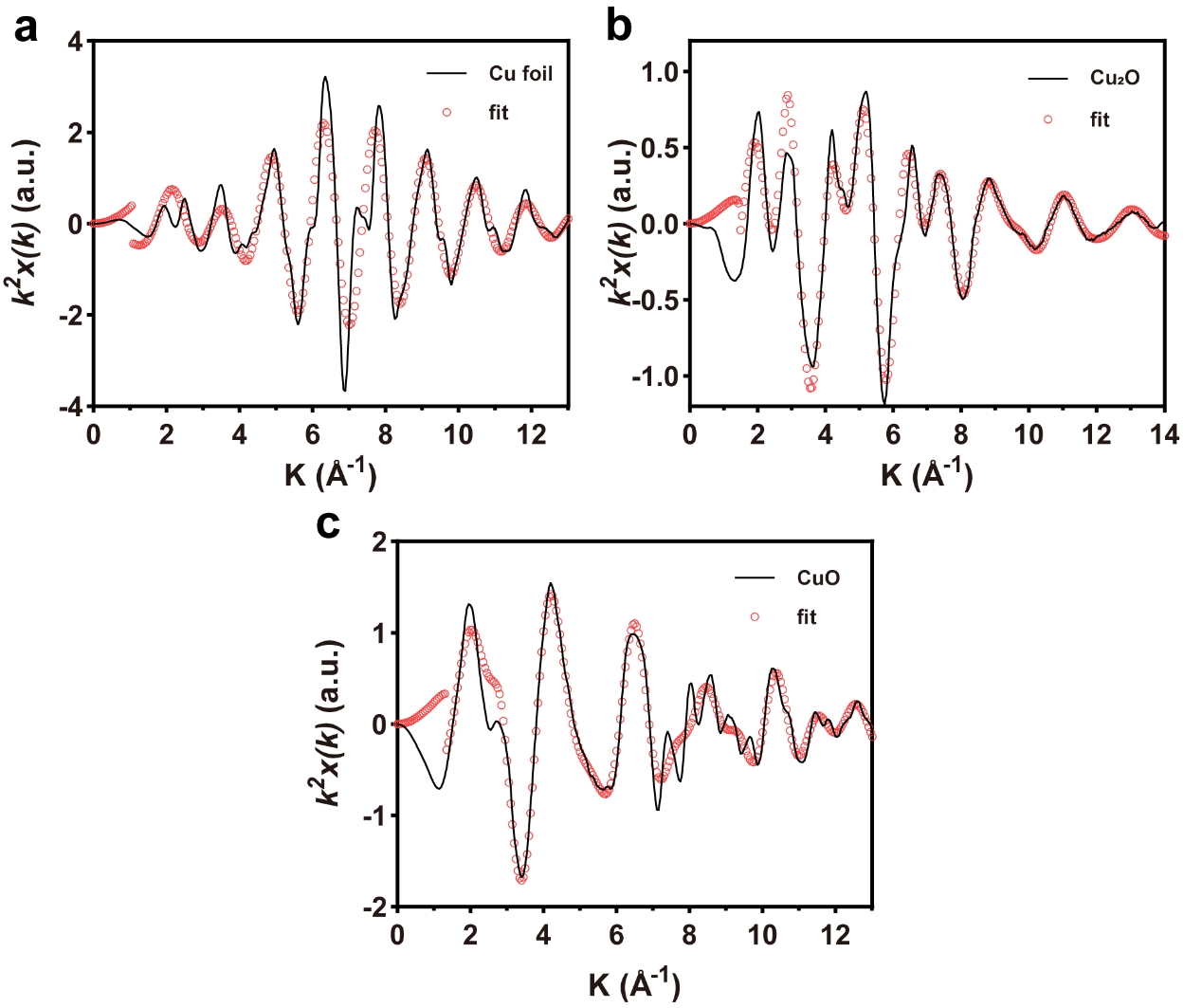


**Figure S5.** K^2^χ(k) space spectra fitting curve of a) Cu foil, b) Cu_2_O, and c) CuO.


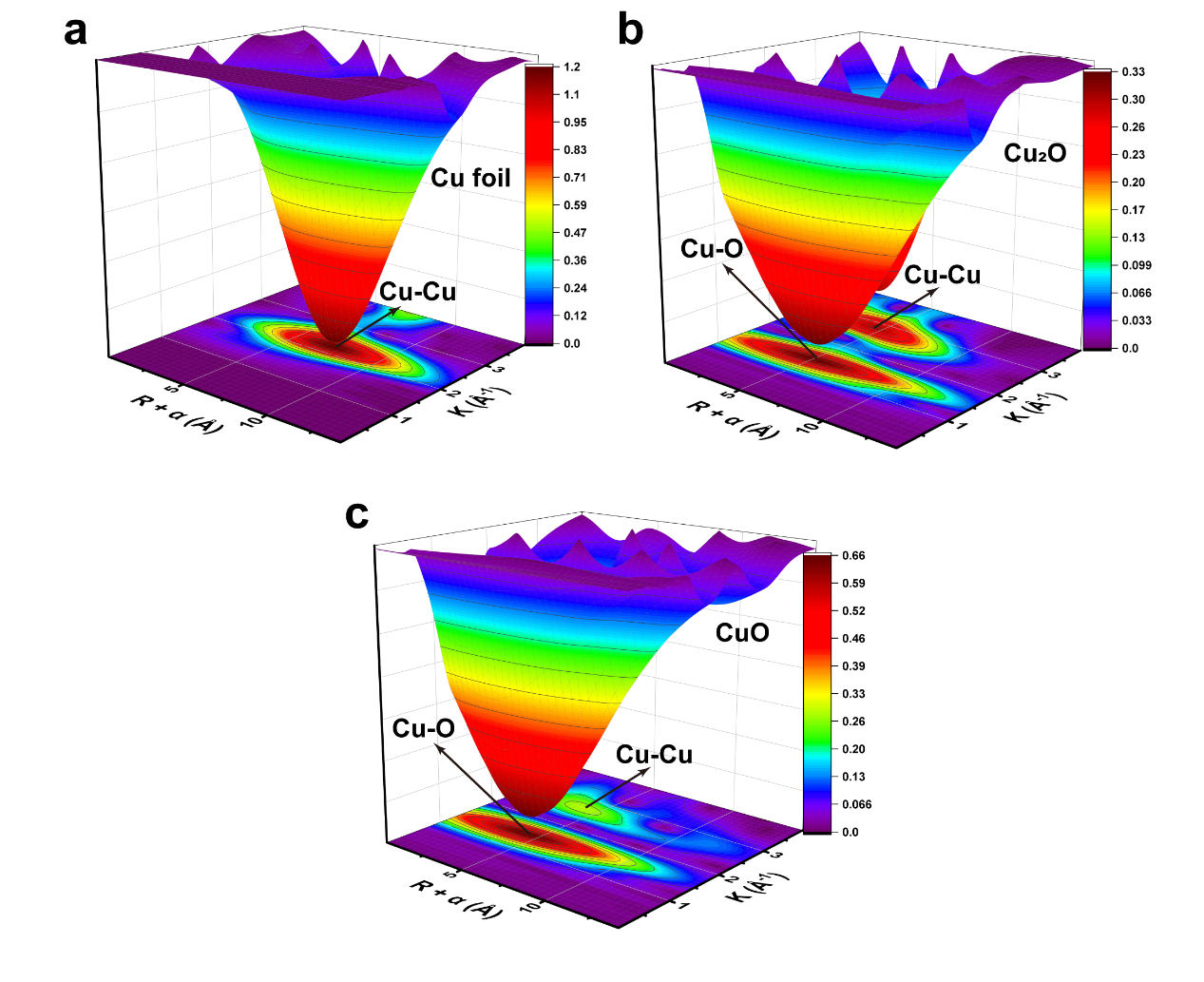


**Figure S6.** WT-EXAFS plots of a) Cu foil, b) Cu_2_O, and c) CuO.


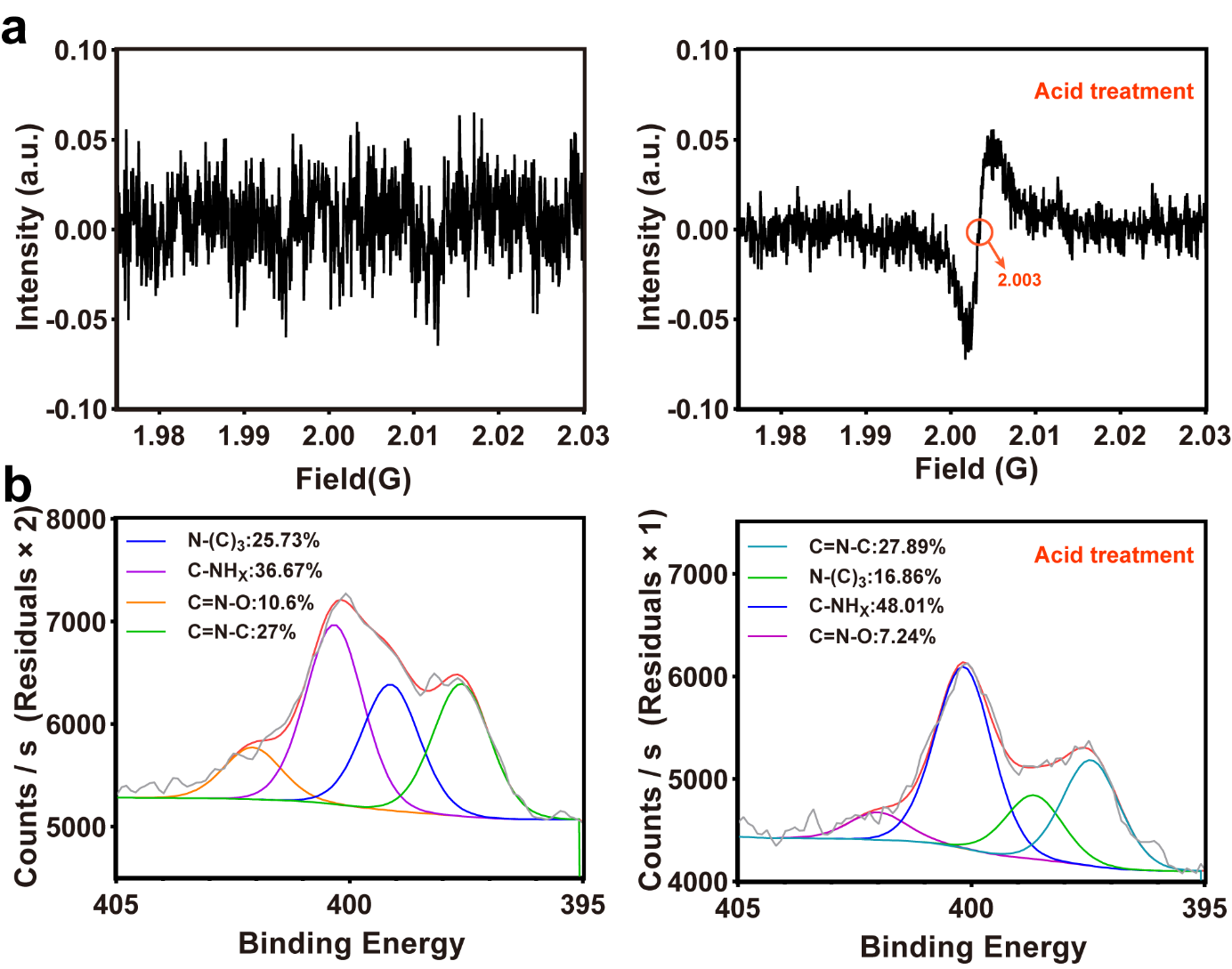


**Figure S7.** a) ESR images of the carbonized samples before and after the hydrochloric acid treatment. b) High-resolution spectrums of N 1s of the samples before and after the acid treatment.


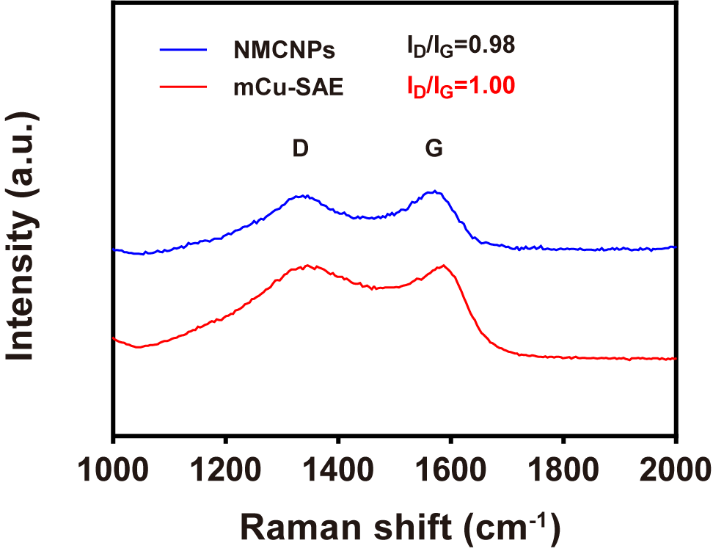


**Figure S8.** Raman spectrum of NMCNPs and mCu-SAE.


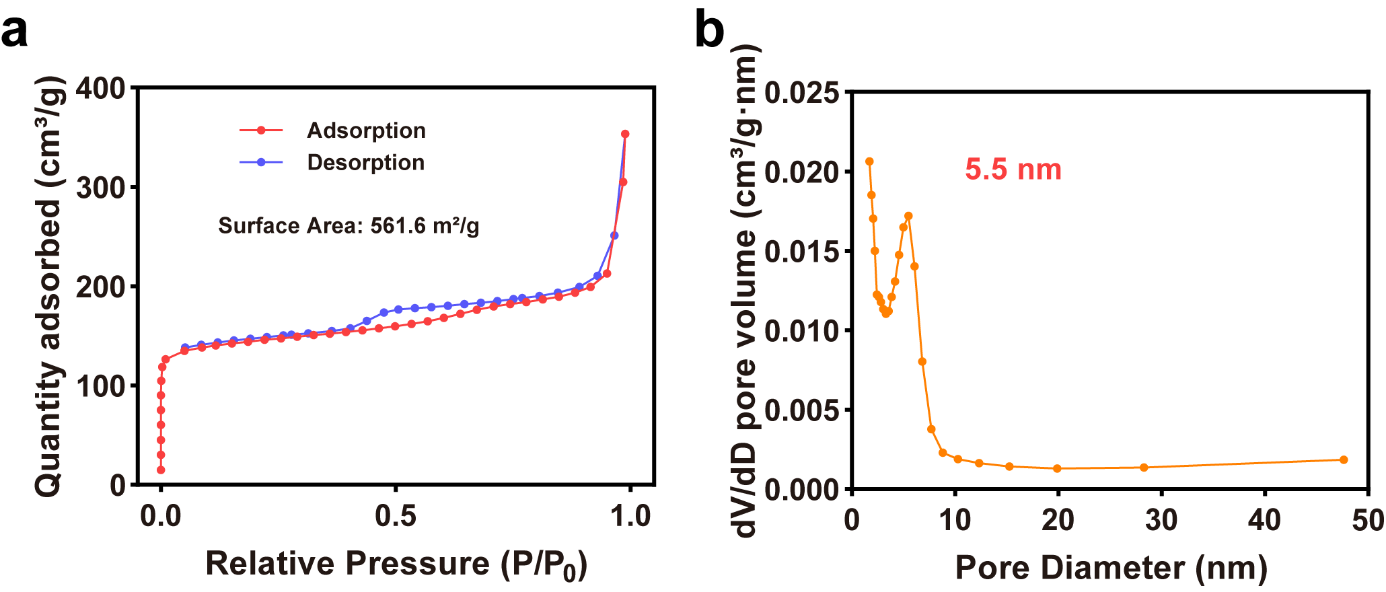


**Figure S9.** a) N_2_ absorption-desorption isotherms of mCu-SAE. b) Pore size distribution of mCu-SAE.


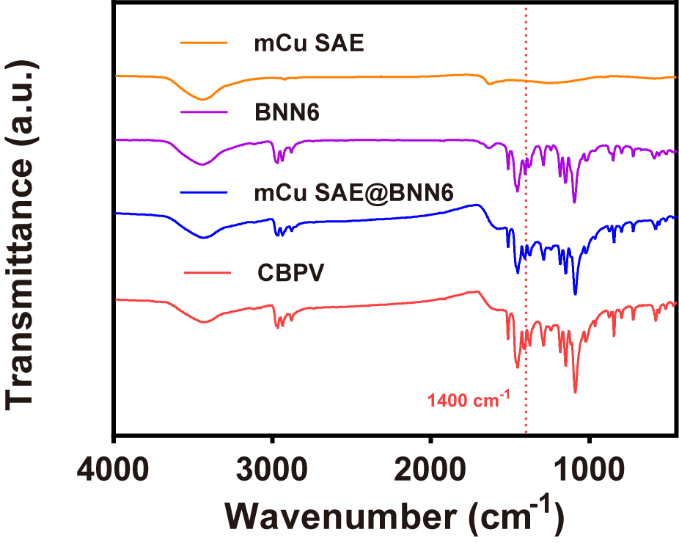


**Figure S10.** FT-IR spectra of BNN6, mCu-SAE, mCu-SAE@BNN6, and CBPV.


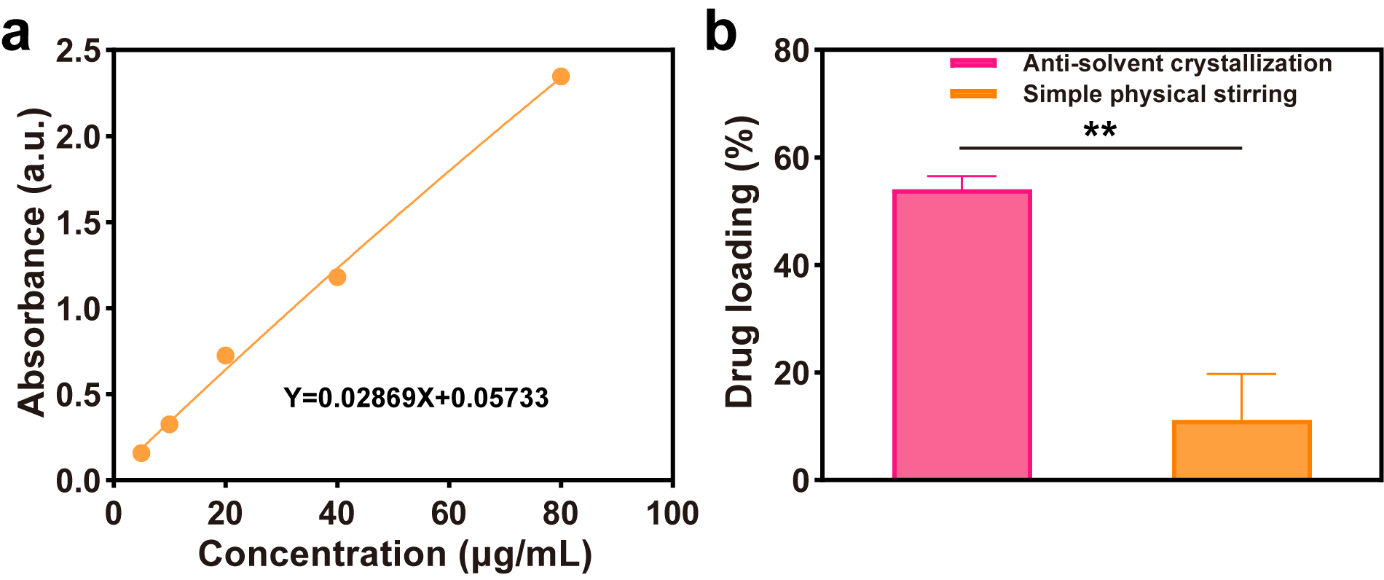


**Figure S11.** a) The concentration-absorbance standard curve of BNN6 . b) Comparison of the anti-solvent crystallization method and simple physical stirring method for drug loading. Data are presented as mean ± SD (*n* = 3). ***p* < 0.01.


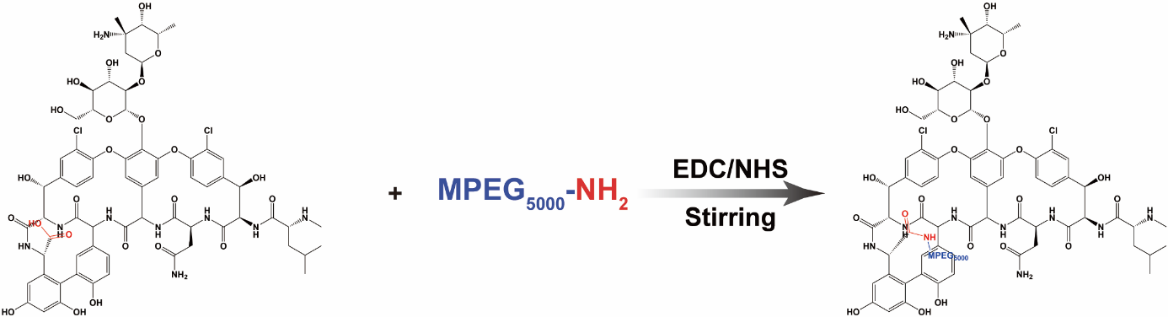


**Figure S12.** The synthesis of the PEG-Van from vancomycin and MPEG_5000_-NH_2_.


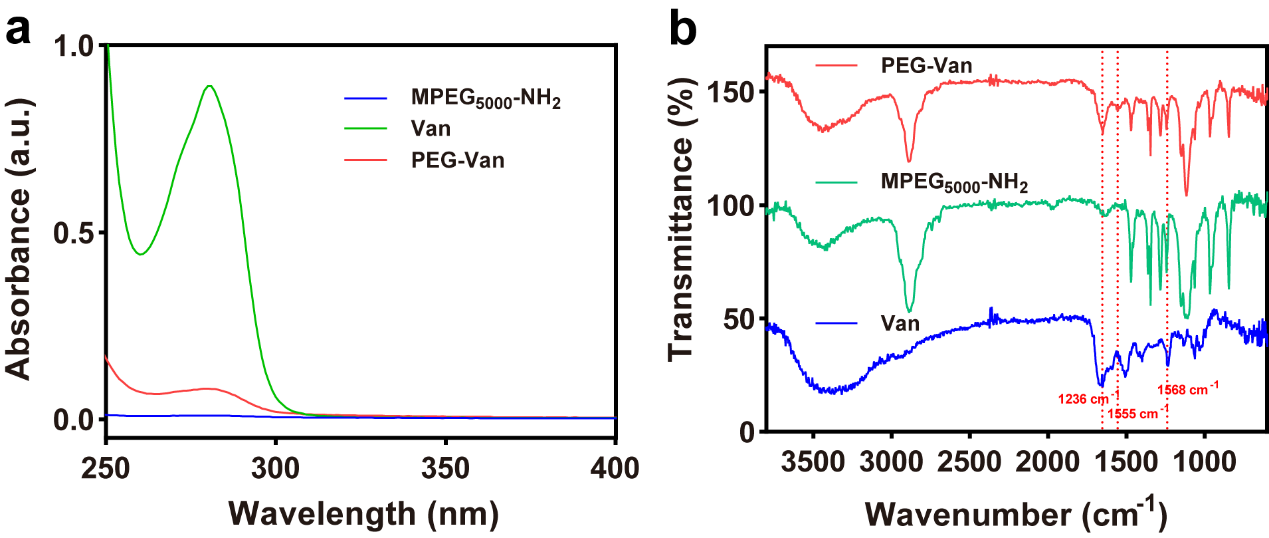


**Figure S13.** a) UV-vis spectra of MPEG_5000_-NH_2_ before and after Van conjugation. b) Infrared spectra of MPEG_5000_-NH_2_ before and after Van conjugation.


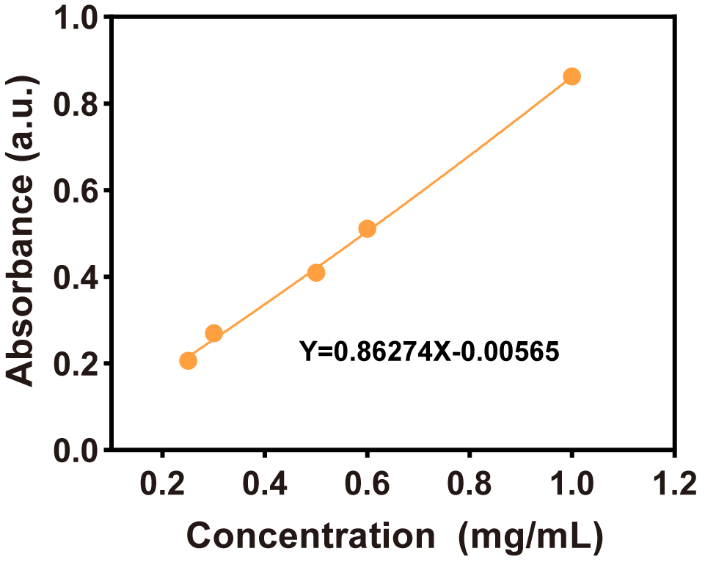


**Figure S14.**  The concentration-absorbance standard curve of PEG-Van.


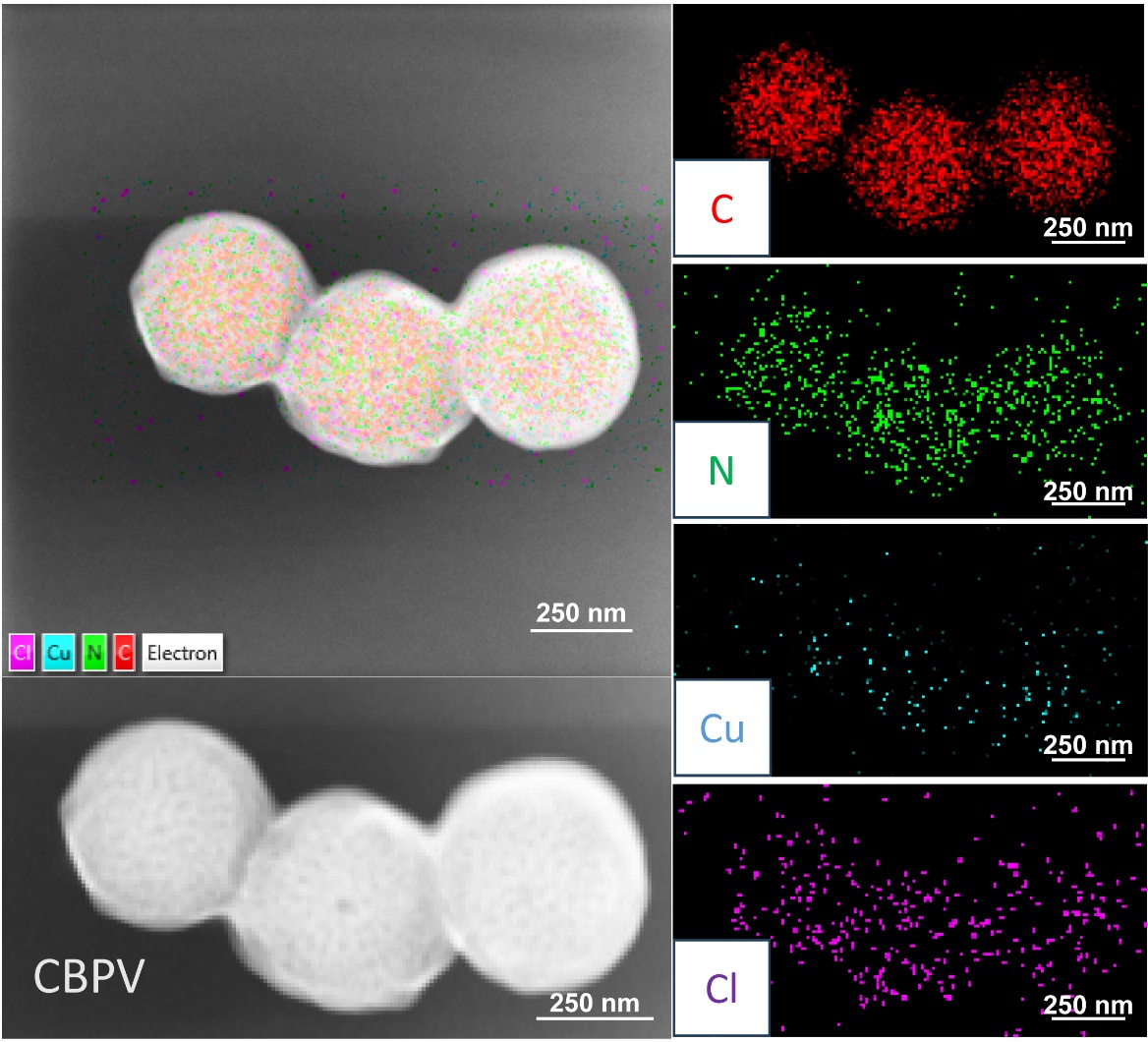


**Figure S15.** Elemental mapping images of CBPV. Scale bar = 250 nm.


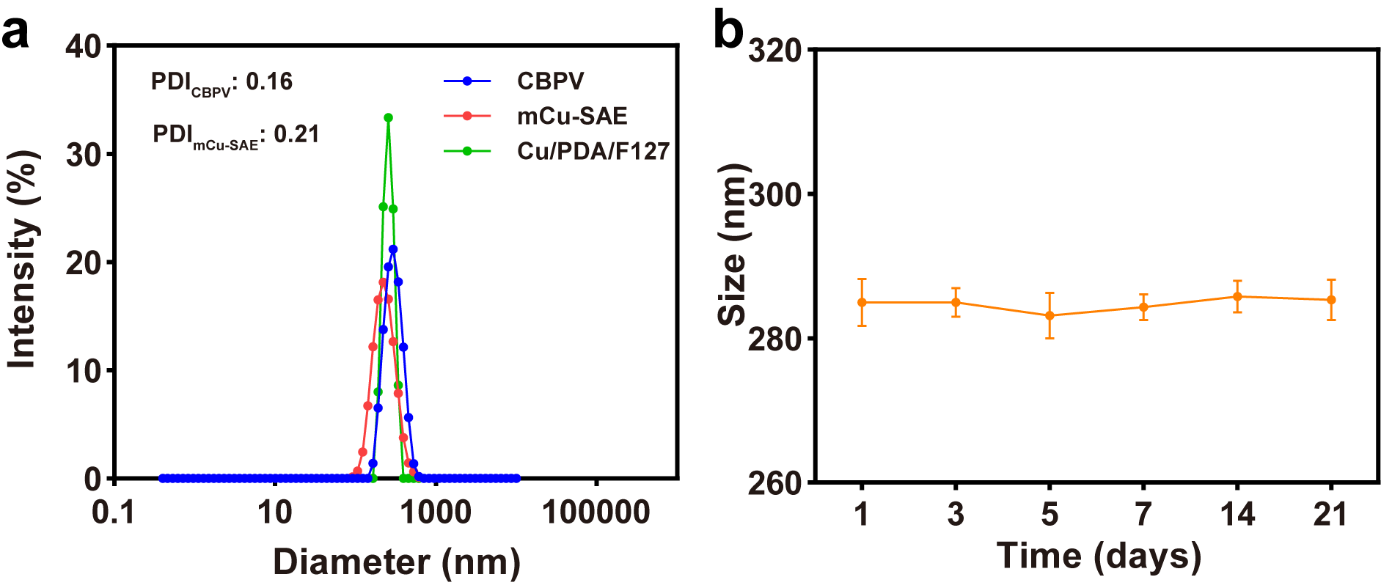


**Figure S16.** a) Hydrodynamic sizes of Cu/PDA/F127, mCu-SAE, and CBPV measured by DLS. b) The stability of CBPV in the aqueous solution over a period of 21 days. Data are presented as mean ± SD (*n* = 3).


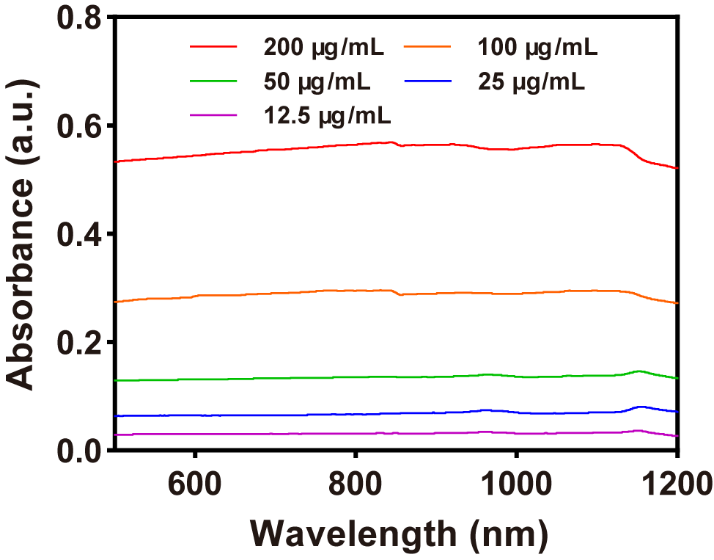


**Figure S17.** UV–vis–NIR spectra of the CBPV with different concentrations.


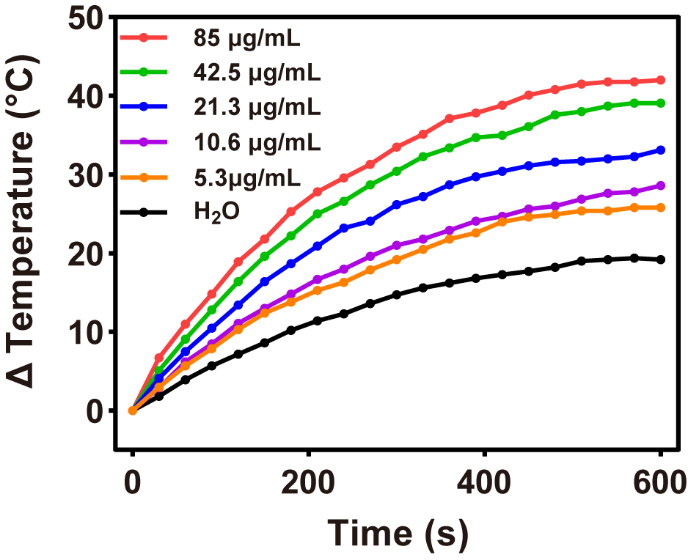


**Figure S18.** Temperature change curves for different concentrations of mCu-SAE irradiated by a 1064 nm laser at 1.25 W cm^-2^.


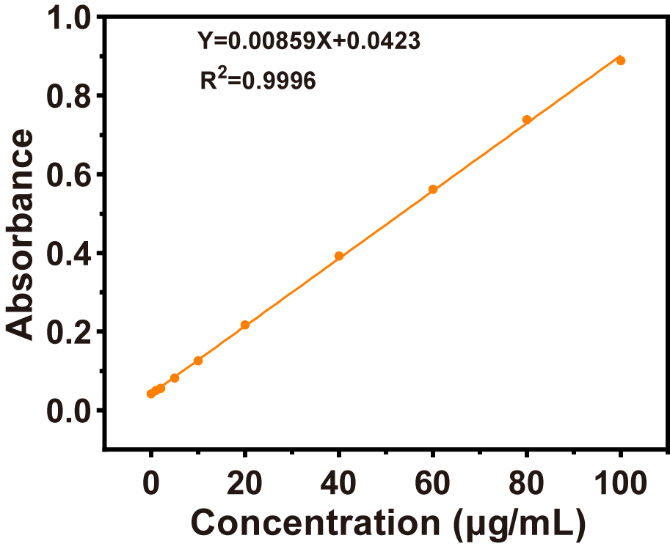


**Figure S19.** The standard absorption curve for NO detection using the Griess method (absorbance values at 540 nm).


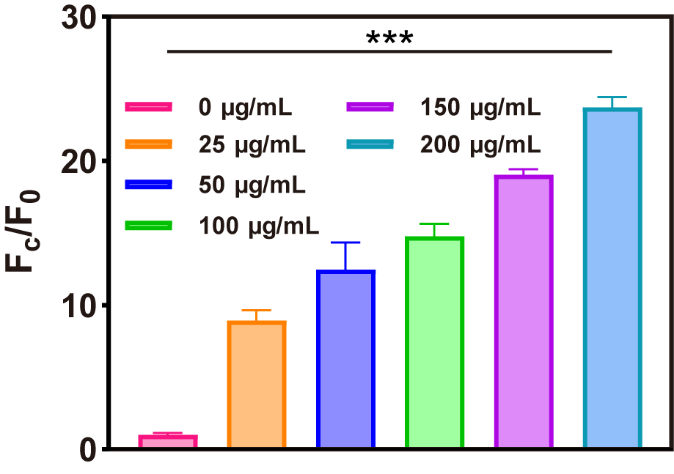


**Figure S20.** Detection of NO production at various concentrations of CBPV using the DAF-FM DA probe. Data are presented as mean ± SD (*n* = 3). ****p* < 0.001.


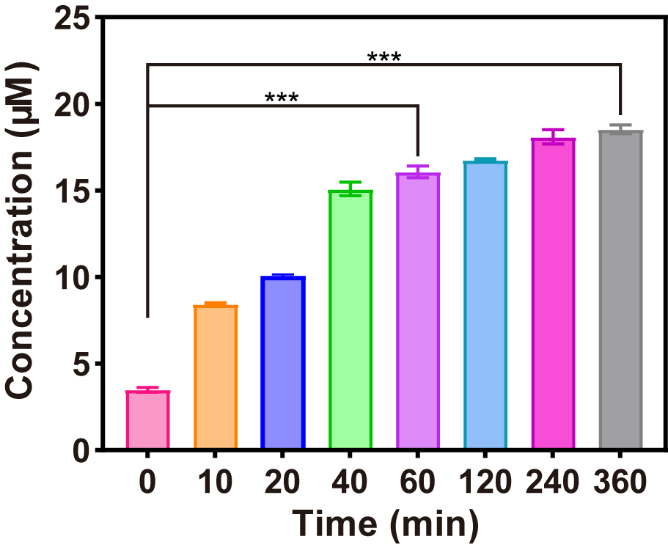


**Figure S21.** NO release from CBPV incubated at 37 °C for 0, 10, 20, 40, 60, 120, 240, and 360 min. Data are presented as mean ± SD (*n* = 3). ****p* < 0.001.


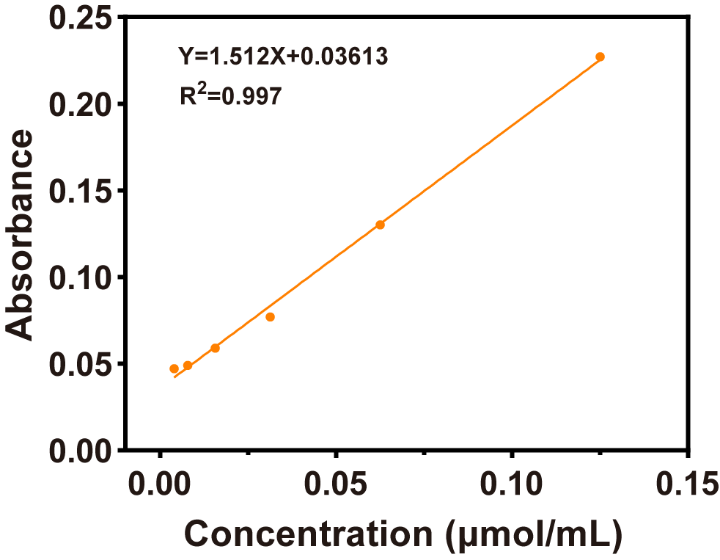


**Figure S22.** Standard absorption curve (the absorbance values at 530 nm) for •O_2_^-^ detection.


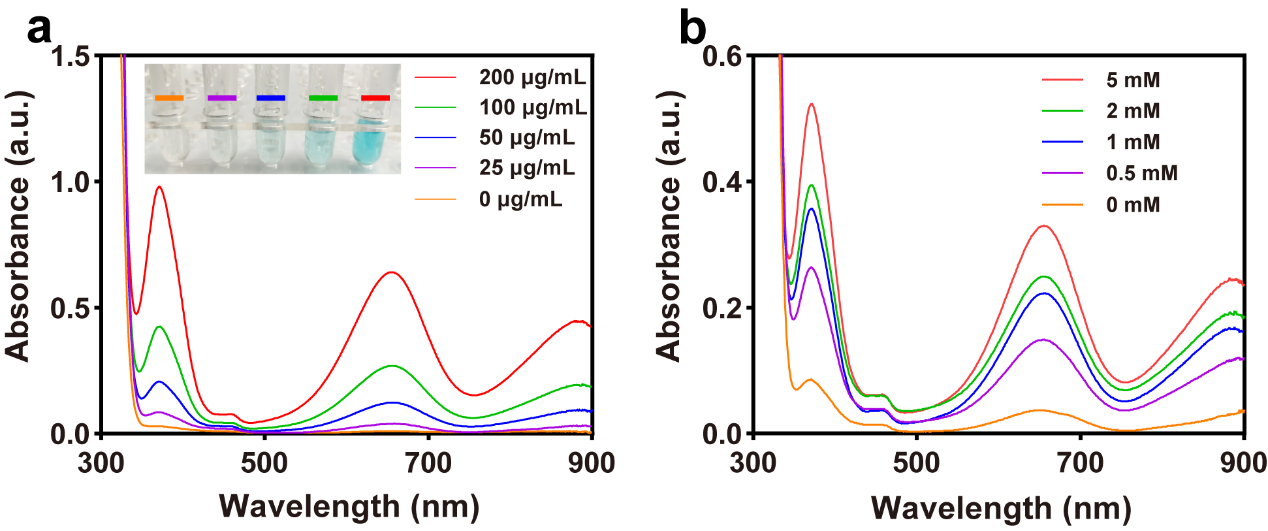


**Figure S23.** a) UV-vis absorption spectra of TMB oxidized by mCu-SAE at different concentrations, with inset showing the colorimetric reaction of TMB. b) UV-vis absorption spectra of TMB oxidized by mCu-SAE (200 μg mL^-1^) and different concentrations of H_2_O_2_.


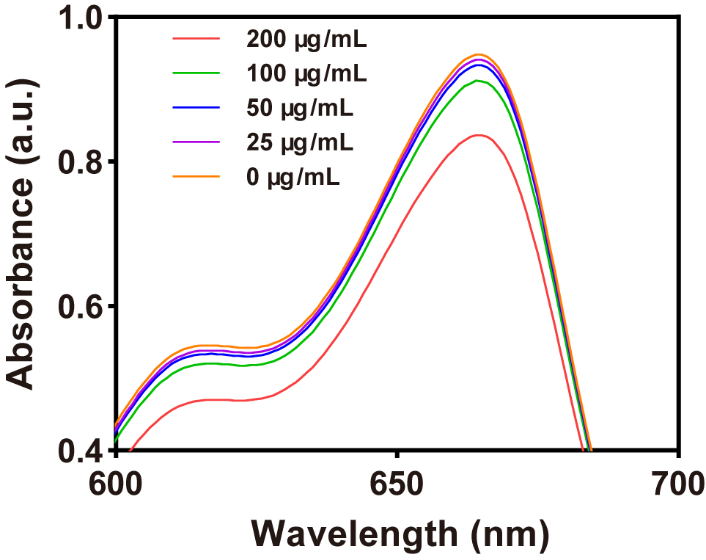


**Figure S24.** MB degradation by ROS generated under different concentrations of the CBPV.


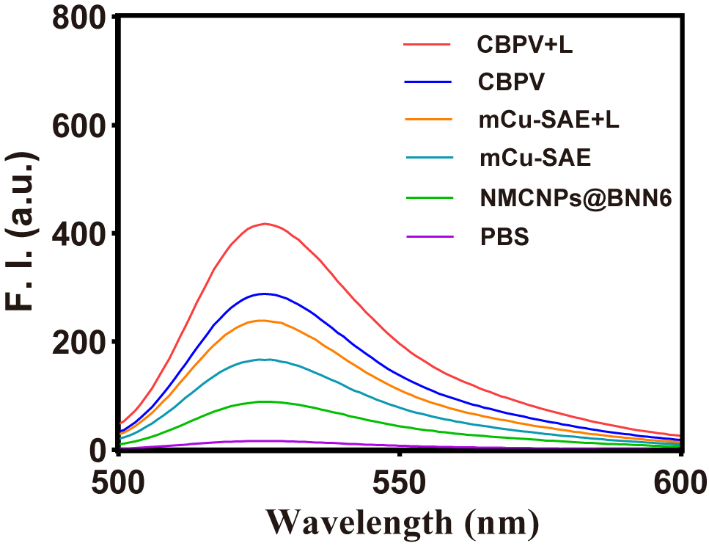


**Figure S25.** Fluorescent spectra of DHR123 solution with different treatments for ONOO^−^ detection.


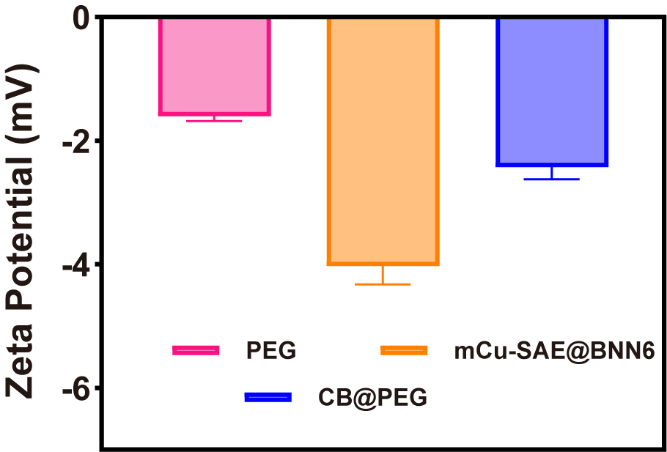


**Figure S26.** Zeta potential of PEG, mCu-SAE@BNN6, and CB@PEG.


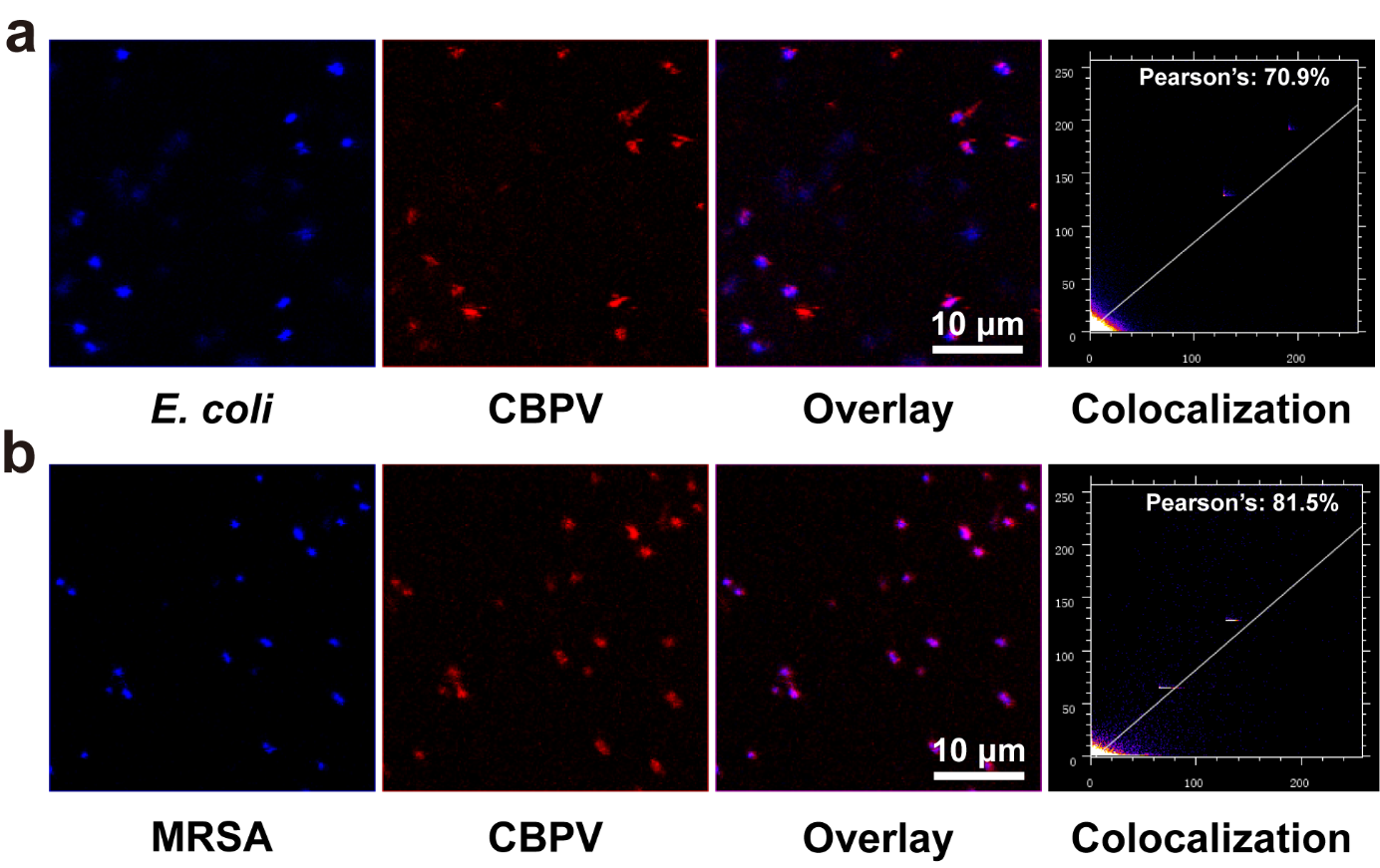


**Figure S27.** Quantitative diagrams of the co-localization rates of CBPV with a) *E. coli* and b) MRSA. Scale bar = 10 μm.


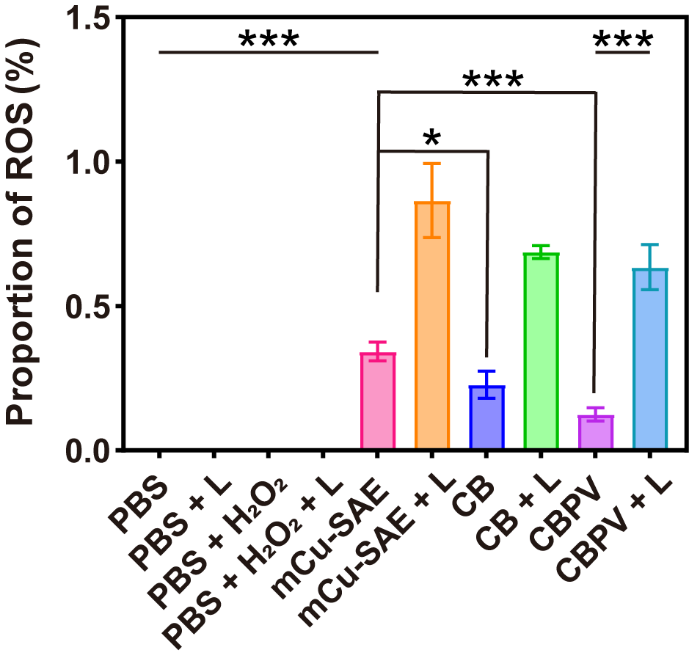


**Figure S28.** Quantitative analysis of ROS generation by various treatments. Data are presented as mean ± SD (*n* = 3). **p* < 0.05, ****p* < 0.001.


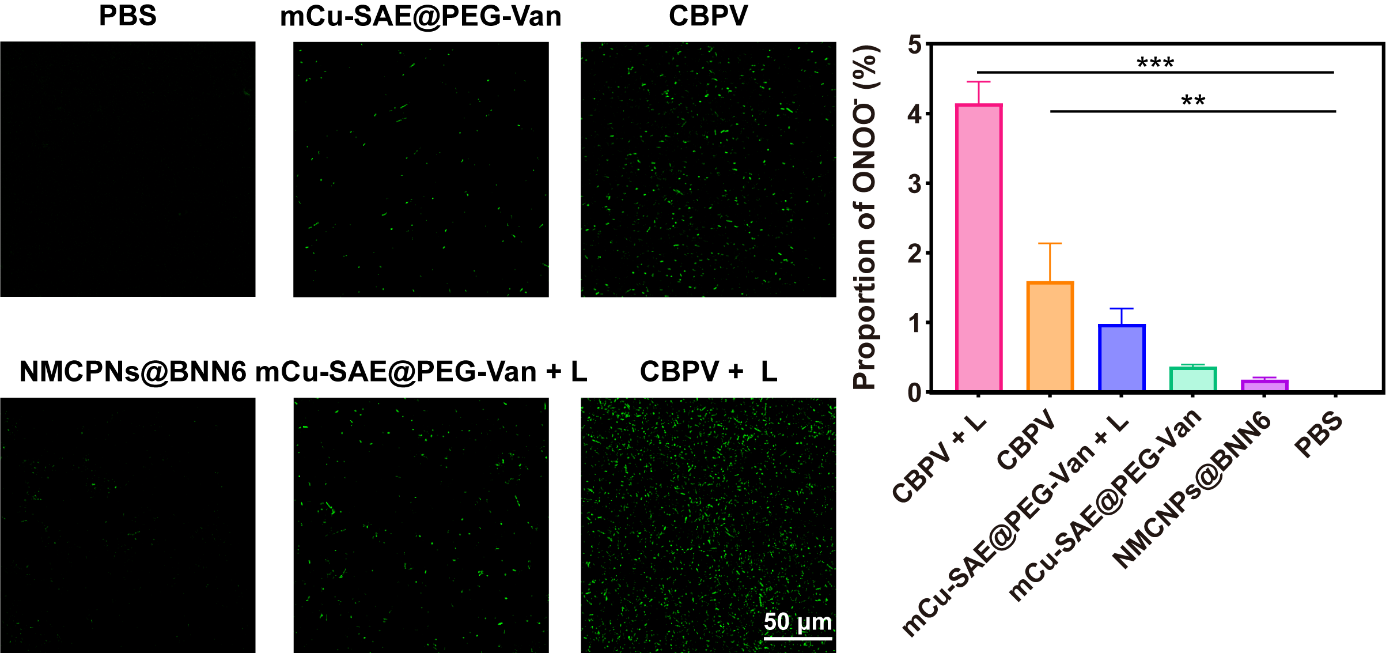


**Figure S29.** DHR123 probe for confocal laser scanning microscopy and quantitative analysis of ONOO^−^ detection under different treatments. Scale bar = 50 μm. Data are presented as mean ± SD (*n* = 3). ***p* < 0.01, ****p* < 0.001.


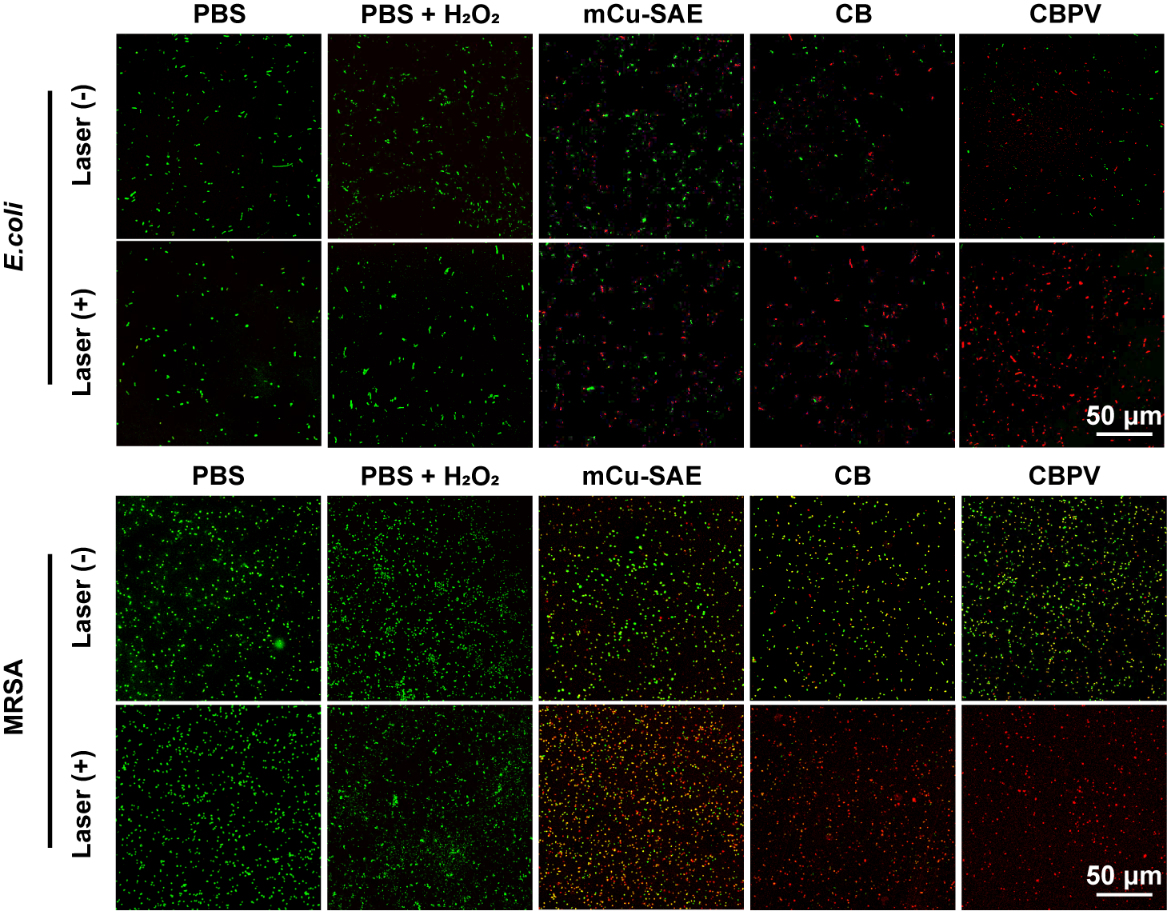


**Figure S30.** Live/dead staining images of *E. coli* and MRSA after various treatments. Scale bar = 50 μm. (Green fluorescence: SYTO-9; Red fluorescence: PI).


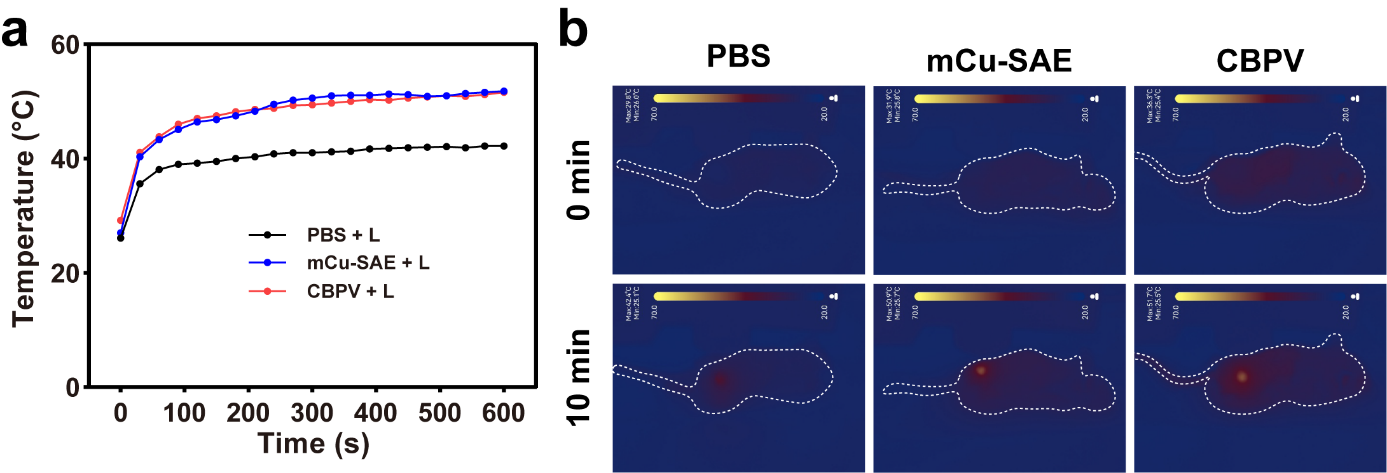


**Figure S31.** a) Temperature change curve at the epidermal wound site during 1064-nm laser irradiation. b) Infrared thermal images of mouse wounds treated with PBS, mCu-SAE, and CBPV under NIR-II laser (1064 nm, 1.25 W cm^-2^) irradiation.


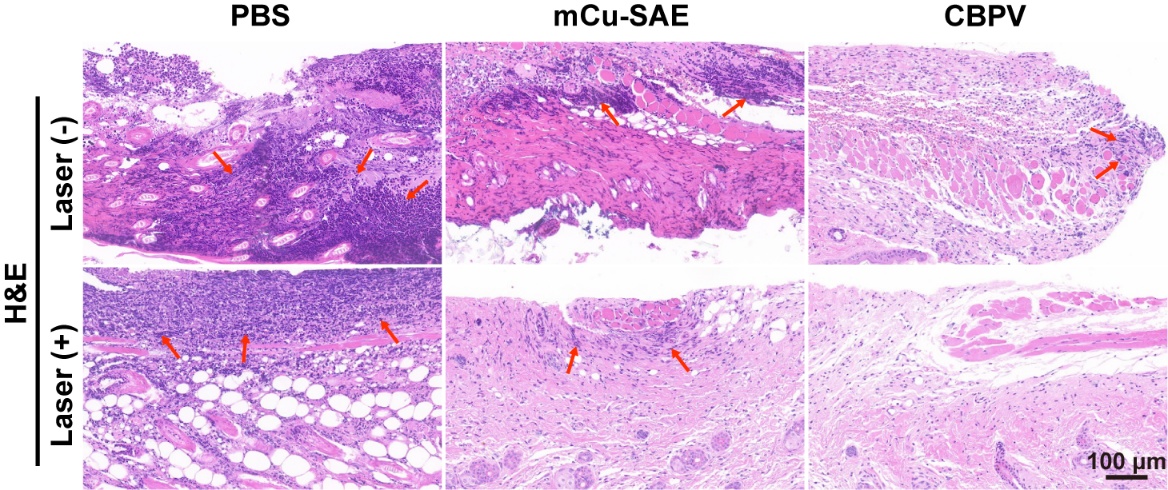


**Figure S32.** H&E staining of regenerated skin surrounding the wound on day 3. Scale bar = 100 μm. (Red arrow: inflammatory cells).


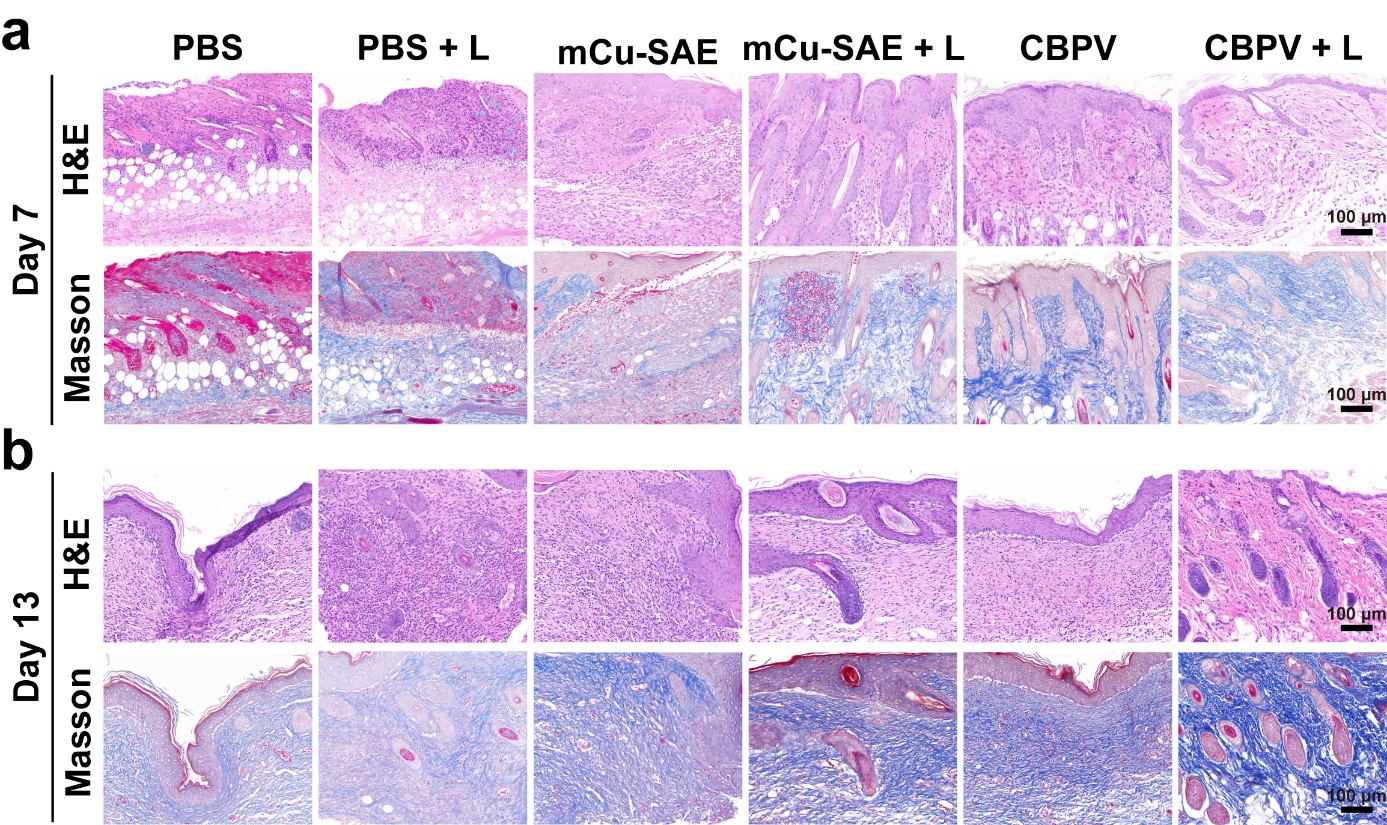


**Figure S33.** a) H&E staining and Masson's trichrome staining of regenerated skin on day 7. Scale bar = 100 μm. b) H&E staining and Masson's trichrome staining of regenerated skin on day 13. Scale bar = 100 μm.


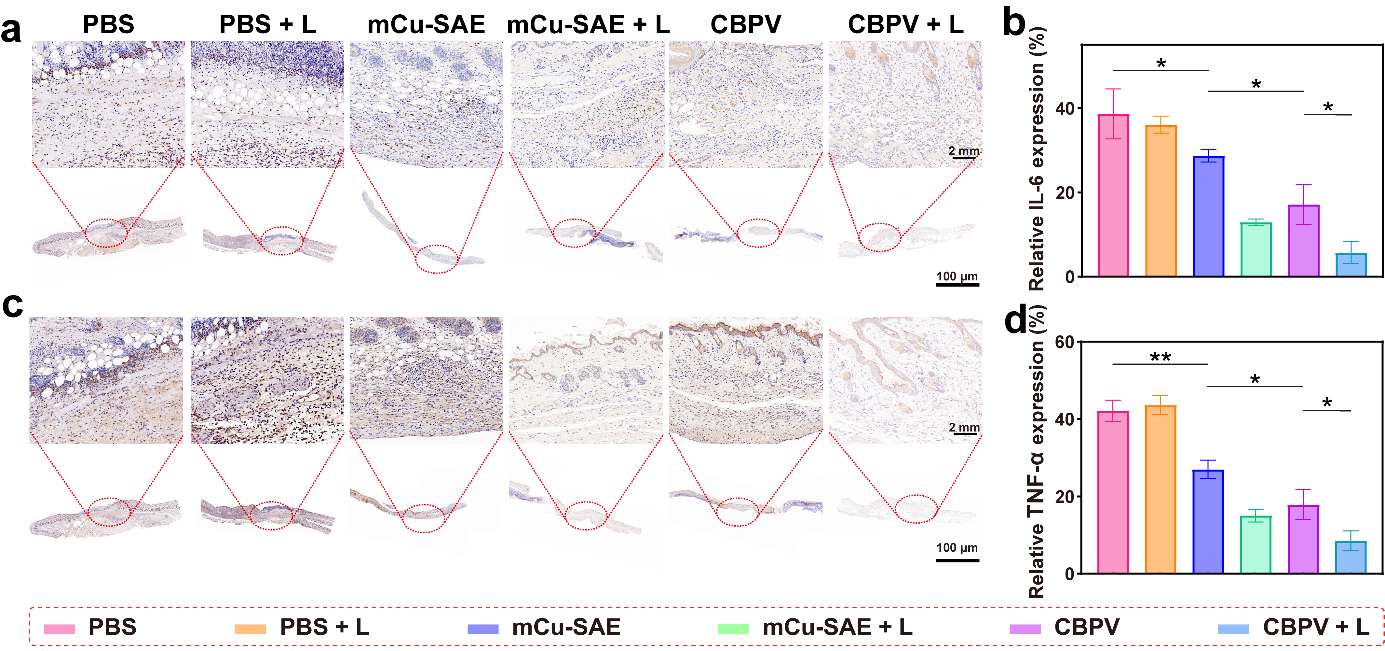


**Figure S34.** a) Immunohistochemical staining of IL-6 in the skin around the wound on day 7 in the epidermal wound model mice. Scale bar = 2 mm. Illustration: Local images of immunohistochemistry. Scale bar = 100 μm. b) Quantitative analysis of IL-6 immunohistochemical staining. c) Immunohistochemical staining of TNF-α in the skin around the wound on day 7 in the epidermal wound model mice. Scale bar = 2 mm. Illustration: Local images of immunohistochemistry. Scale bar = 100 μm. d) Quantitative analysis of TNF-α immunohistochemical staining. Data are presented as mean ± SD (*n* = 3). **p* < 0.05 ***p* < 0.01.


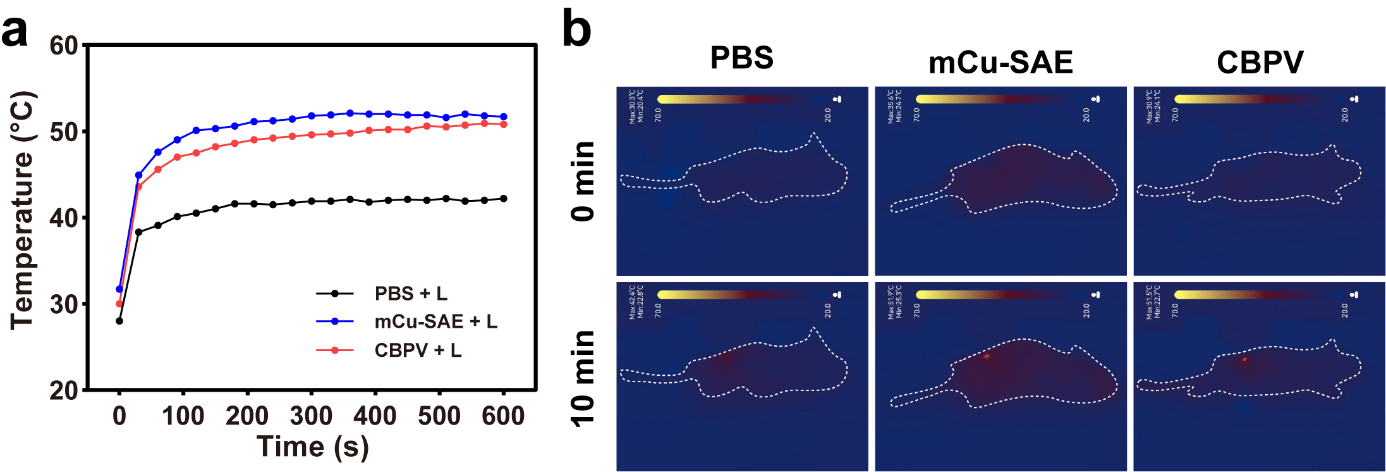


**Figure S35.** a) Temperature change curve at the subcutaneous cyst site during 1064-nm laser irradiation. b) Infrared thermal images of subcutaneous cysts in mice treated with PBS, mCu-SAE, and CBPV under NIR-II laser (1064 nm, 1.25 W cm^-2^) irradiation.


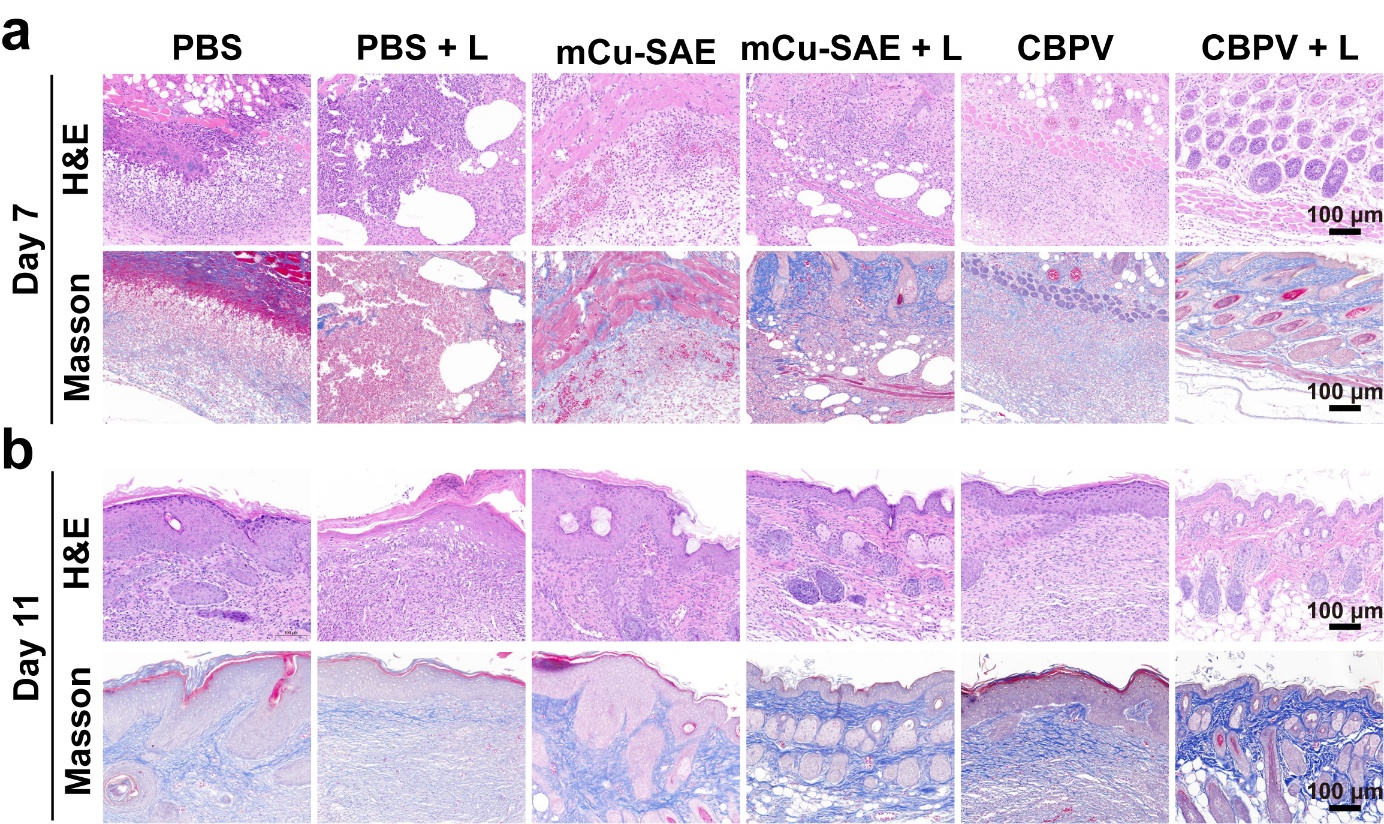


**Figure S36.** a) H&E staining and Masson's trichrome staining of regenerated skin on day 7. Scale bar = 100 μm. b) H&E staining and Masson's trichrome staining of regenerated skin on day 11. Scale bar = 100 μm.


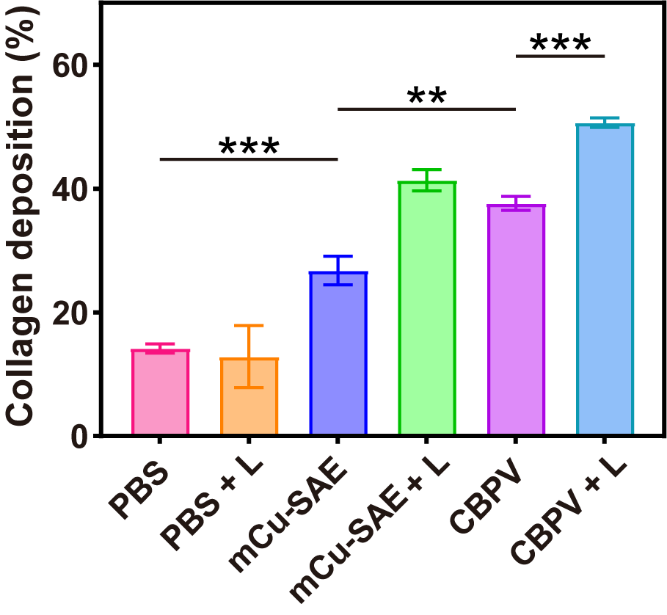


**Figure S37.** Quantitative analysis of collagen deposition in the subcutaneous cyst model. Data are presented as mean ± SD (*n* = 3). ***p* < 0.01, ****p* < 0.001.


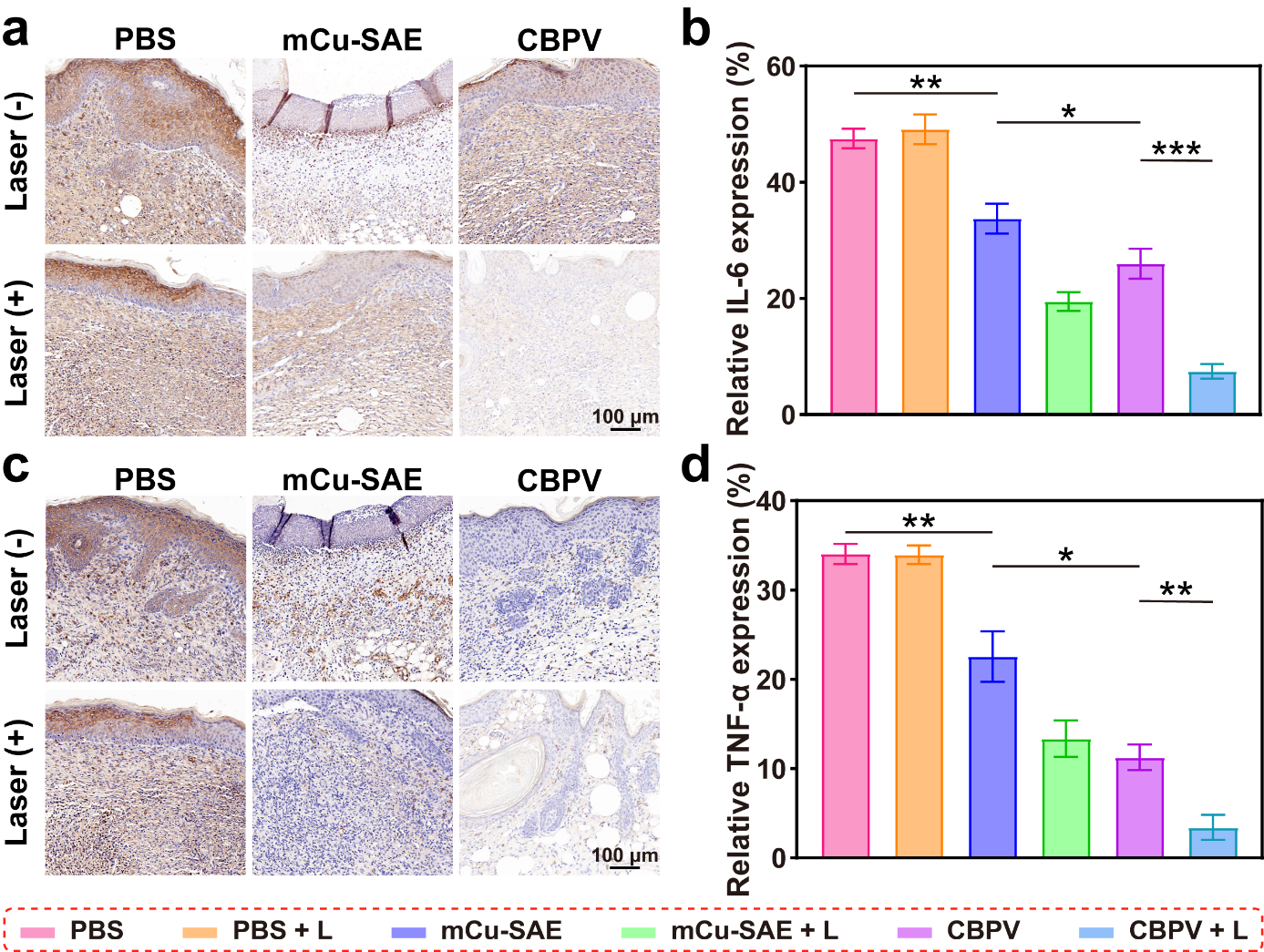


**Figure S38.** a) Immunohistochemical staining of IL-6 in the skin around the cyst on day 11 in the subcutaneous cyst model mice. Scale bar = 100 μm. b) Quantitative analysis of IL-6 immunohistochemical staining. c) Immunohistochemical staining of TNF-α in the skin around the cyst on day 11 in the subcutaneous cyst model mice. Scale bar = 100 μm. d) Quantitative analysis of TNF-α immunohistochemical staining. Data are presented as mean ± SD (n = 3). **p* < 0.05, ***p* < 0.01, ****p* < 0.001.


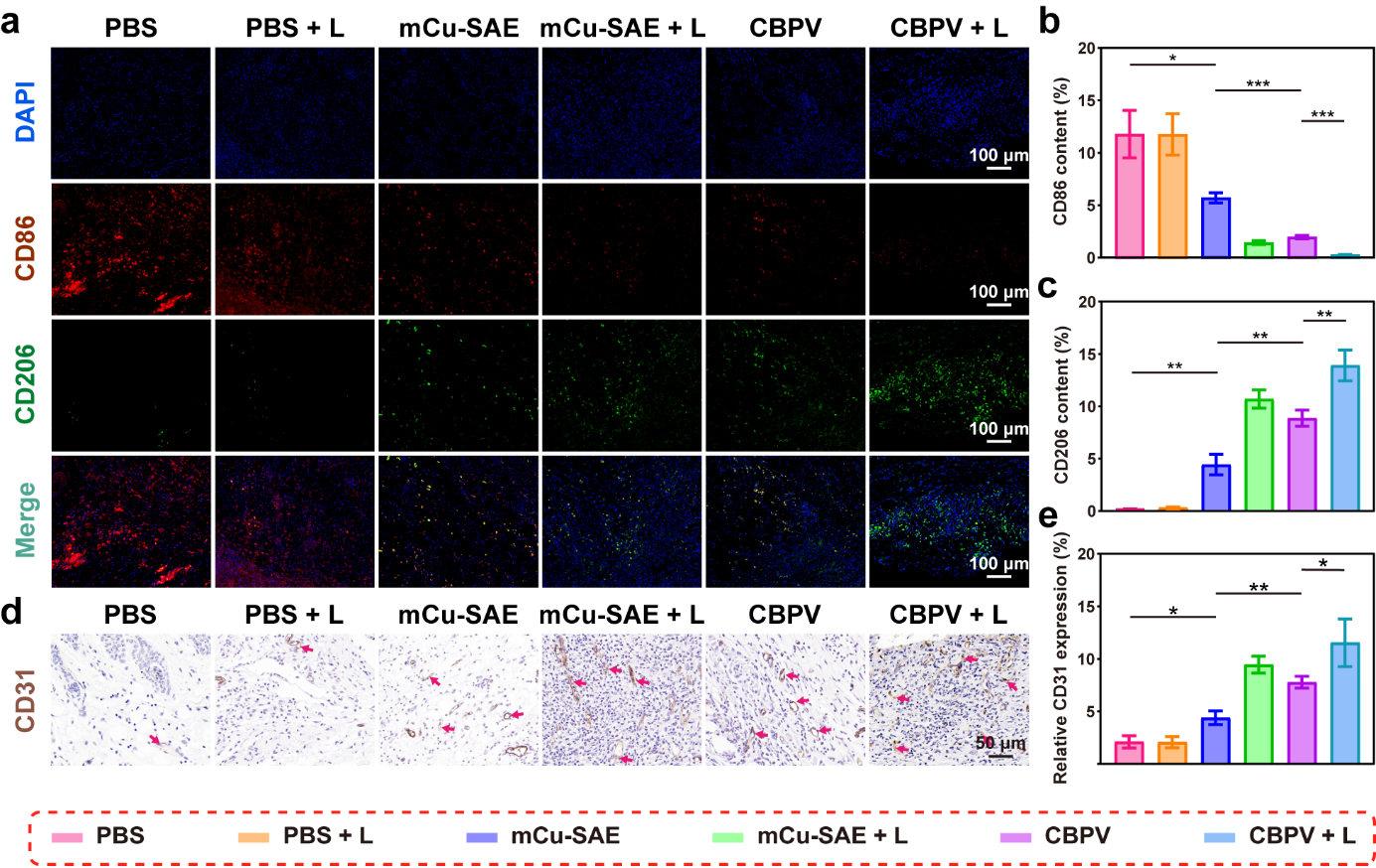


**Figure S39.** Immunofluorescence and immunohistochemical analyses of regenerated skin tissue surrounding subcutaneous cysts in treated model mice. a) Immunofluorescence staining was performed to evaluate the expression of CD86 and CD206 in the regenerated skin around the cysts in each group on day 11 post-treatment. Scale bar = 100 μm. b) Quantitative analysis of CD86. c) Quantitative analysis of CD206. d) Immunohistochemical analysis of CD31 expression on day 11. Scale bar = 50 μm. (Pink arrow: CD31-positive endothelial cells). e) Quantitative analysis of CD31 in the subcutaneous cyst model. Data are presented as mean ± SD (*n* = 3). **p* < 0.05, ***p* < 0.01, ****p* < 0.001.


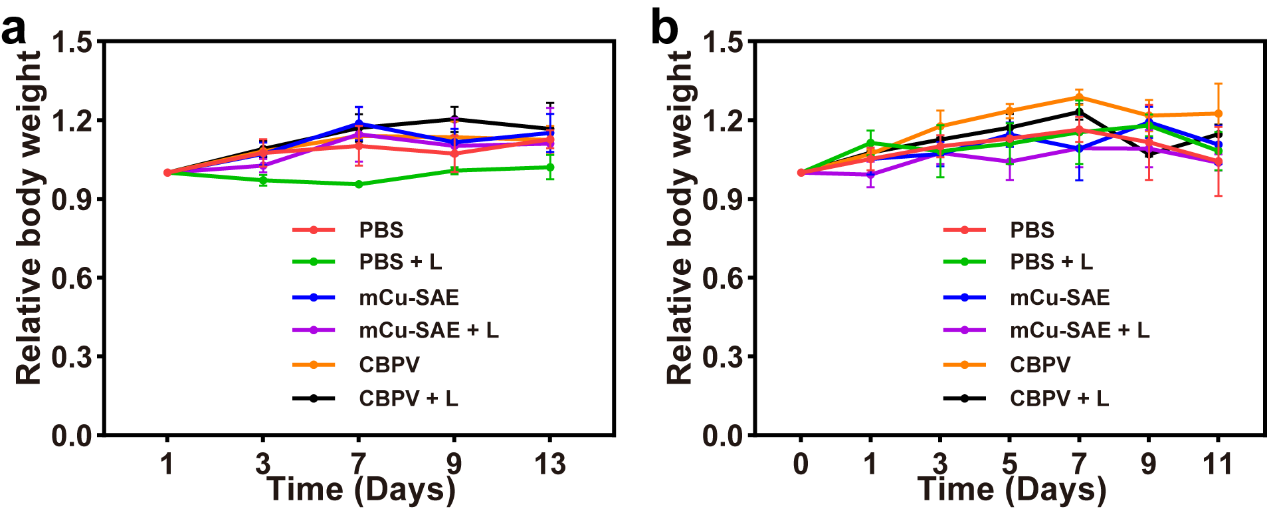


**Figure S40.** Body weight of mice in different treatment groups for a) diabetic epidermal wound and b) subcutaneous cyst models. Data are presented as mean ± SD (*n* = 3).


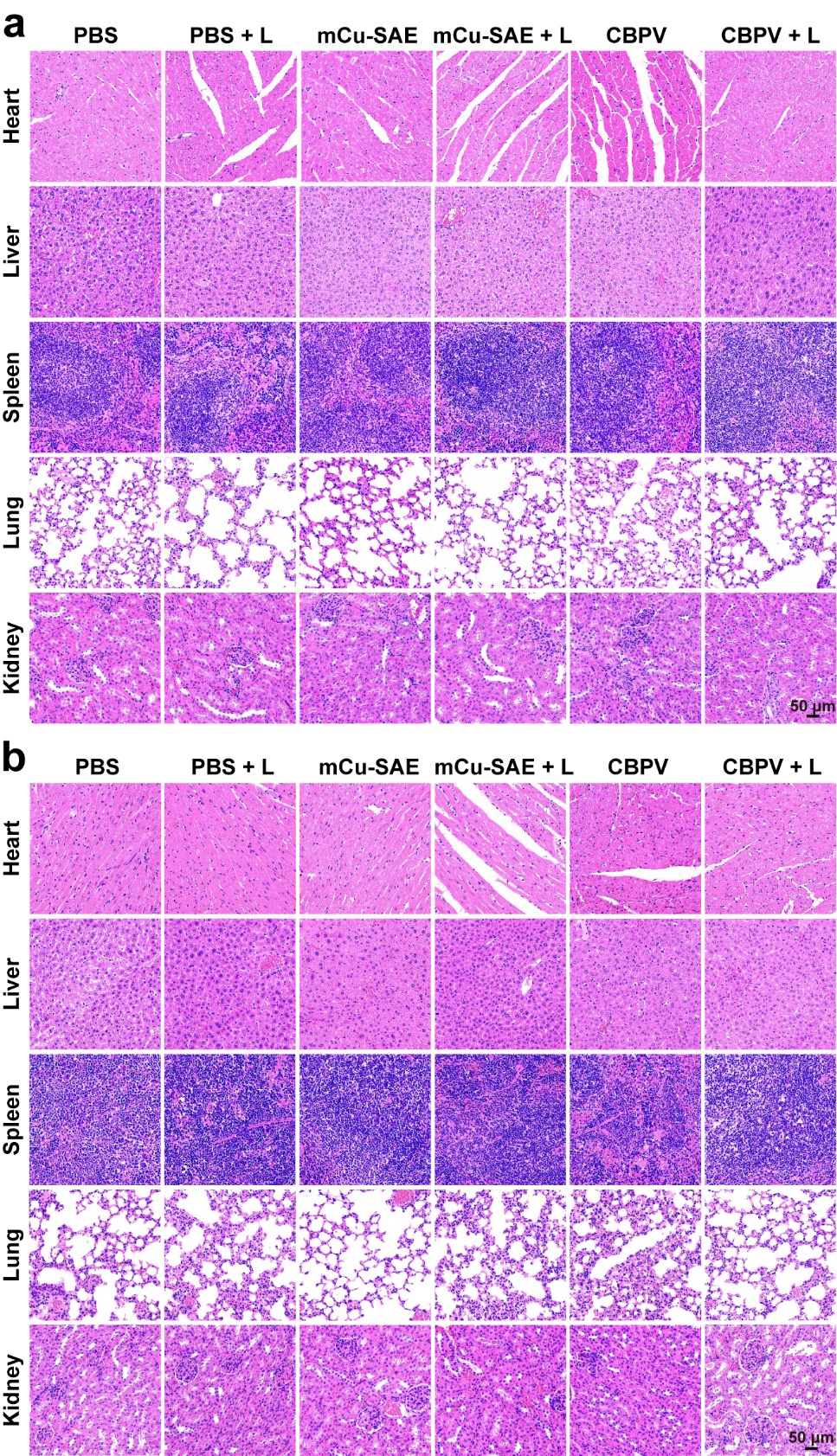


**Figure S41.** a) H&E staining of major organs from different groups of mice after treatments in the epidermal wound model. Scale bar = 50 μm. b) H&E staining of major organs from different groups of mice after treatments in the subcutaneous cyst model. Scale bar = 50 μm.


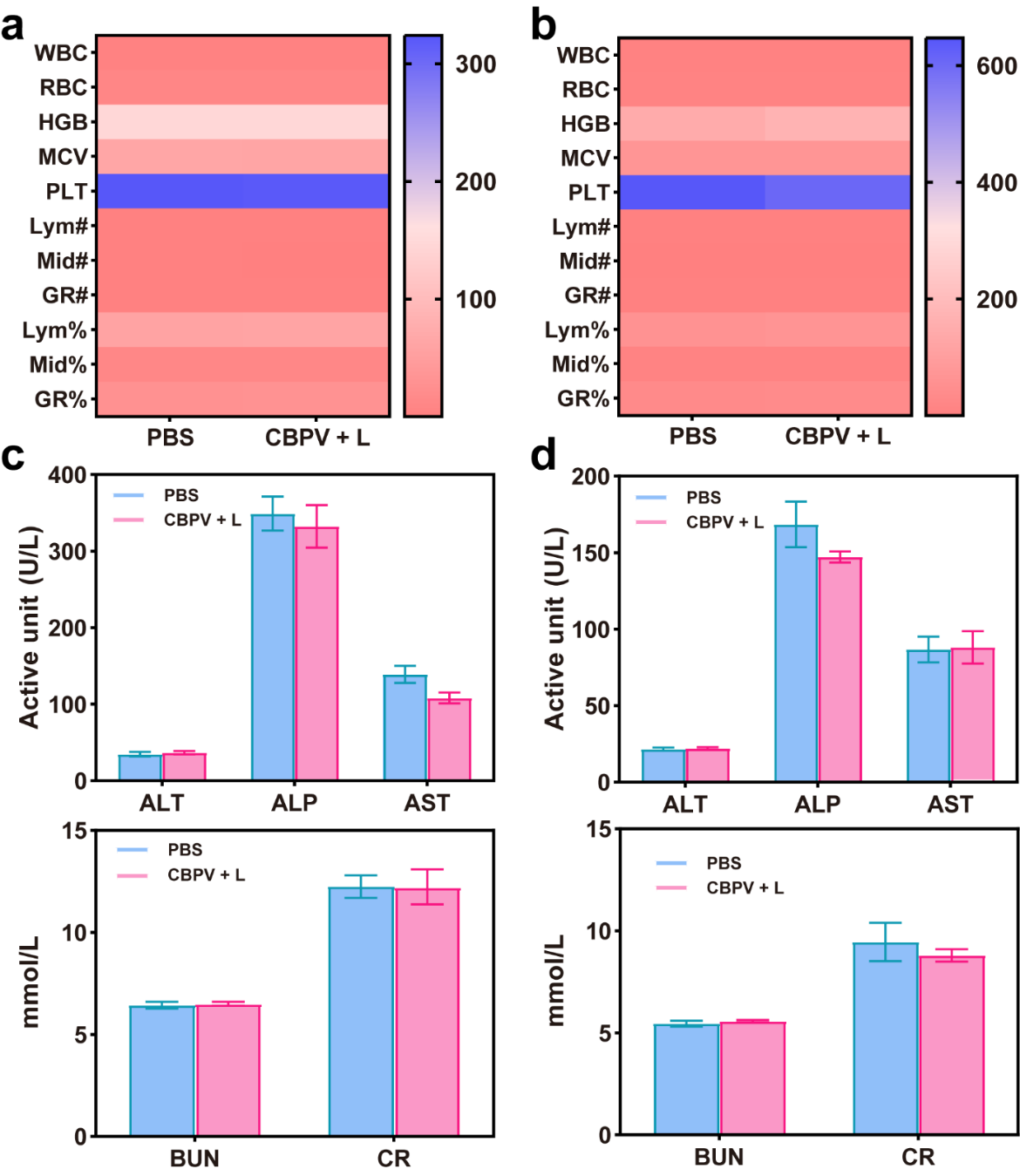


**Figure S42.** a) and b) Analysis of relevant blood routine indicators in the epidermal wound model and subcutaneous cyst model mice, respectively. c) and d) Blood biochemical analysis of liver and kidney-related indicators in the epidermal wound model and subcutaneous cyst model mice, respectively. (ALT: alanine aminotransferase, ALP: alkaline phosphatase, AST: aspartate aminotransferase, BUN: urea, CR: creatinine). Data are presented as mean ± SD (*n* = 3).

**Supporting Table**

**Table S1.** EXAFS fitting parameters at the Cu *K*-edge for various sample (*Ѕ*_0_^2f)^=0.87)

| Sample | Shell | *CN*^a)^ | *R*(Å)^b)^ | *σ*^2^(Å^2^)^c)^ | Δ*E*_0_(eV)^d)^ | *R* factor^e)^ |
| --- | --- | --- | --- | --- | --- | --- |
| Cu foil | Cu-Cu | 12^g)^ | 2.54±0.01 | 0.0088 | 4.5±0.5 | 0.0029 |
| mCu-SAE | Cu-N | 3.2±0.2 | 1.95±0.01 | 0.0101 | 5.0±1.7 | 0.0071 |
| Cu_2_O | Cu-O | 1.7±0.1 | 1.85±0.01 | 0.0026 | 8.2±0.8 | 0.0067 |
|  | Cu-Cu | 9.4±0.7 | 3.02±0.01 | 0.0193 |  |  |
|  | Cu-O1 | 4.6±0.8 | 3.54±0.03 | 0.0116 |  |  |
| CuO | Cu-O | 3.9±0.1 | 1.95±0.01 | 0.0041 | 6.7±0.4 | 0.0017 |
|  | Cu-Cu | 1.4±0.1 | 2.89±0.01 | 0.0042 |  |  |
|  | Cu-Cu | 3.5±0.4 | 3.42±0.01 | 0.0044 |  |  |
|  | Cu-O1 | 6.1±0.6 | 3.54±0.02 | 0.0010 |  |  |

a) coordination number. b) the distance to the neighboring atom. c) the Mean Square Relative Displacement (MSRD). d) inner potential correction. e) *R* factor indicates the goodness of the fit. f) *S*_0_^2^ was fixed to 0.87, according to the experimental EXAFS fit of Cu foil by fixing *CN* as the known crystallographic value. g) This value was fixed during EXAFS fitting, based on the known structure of Cu. Fitting range: 3.0 ≤ *k* (/Å) ≤ 13.4 and 1.0 ≤ *R* (Å) ≤ 3.0 (Cu foil); 3.0 ≤ *k* (/Å) ≤ 10.2 and 1.0 ≤ *R* (Å) ≤ 2.1 (mCu-SAE); 3.0 ≤ *k* (/Å) ≤ 11.5 and 1.0 ≤ *R* (Å) ≤ 3.5 (Cu_2_O); 3.0 ≤ *k* (/Å) ≤ 11.5 and 1.0 ≤ *R* (Å) ≤ 3.5 (CuO). A reasonable range of EXAFS fitting parameters: 0.700 < *Ѕ*_0_^2^ < 1.000; *CN* > 0; *σ*^2^ > 0 Å^2^; |Δ*E*_0_| < 10 eV; *R* factor < 0.02.

**Table S2.** The kinetic parameters of Cu_3_P/CDs, Fe_3_O_4_/CDs, FeP, and CBPV.

| **Catalyst** | **Substrate** | $\text{K}\text{m}$ **/ mM** | ***V*_max_ / (10^-6^ Μ s^-1^)** | **Ref.** |
| --- | --- | --- | --- | --- |
| CBPV | TMB | 2.035 | 0.82 | This work |
|  | H_2_O_2_ | 0.099 | 0.556 |  |
| Cu_3_P/CDs | TMB | 16.05 | 0.084 | [2] |
|  | H_2_O_2_ | 45.74 | 0.268 |  |
| Fe_3_O_4_/CDs | TMB | 22.34 | 0.53 | [3] |
|  | H_2_O_2_ | 59.14 | 0.071 |  |
| FeP | TMB | / | / | [4] |
|  | H_2_O_2_ | 0.29 | 0.052 |  |

**References**

[1] a) H. Wang, Z. Zhang, X. Wang, X. Jin, X. Gao, L. Yu, Q. Han, Z. Wang, J. Song, *Nano Lett.* **2024**, *24*, 9700; b) Y. Su, F. Wu, Q. Song, M. Wu, M. Mohammadniaei, T. Zhang, B. Liu, S. Wu, M. Zhang, A. Li, J. Shen, *Biomaterials* **2022**, *281*, 121325; c) J. Ye, W. Lv, C. Li, S. Liu, X. Yang, J. Zhang, C. Wang, J. Xu, G. Jin, B. Li, Y. Fu, X. Liang, *Adv. Funct. Mater.* **2022**, *32*, 2206157.

[2] J. Dong, G. Liu, Y. V. Petrov, Y. Feng, D. Jia, V. E. Baulin, A. Yu Tsivadze, Y. Zhou, B. Li, *Adv.* *Healthcare Mater.* **2024**, *13*, 2402568.

[3] L. Jiao, J. B. Wu, H. Zhong, Y. Zhang, W. Q. Xu, Y. Wu, Y. F. Chen, H. Y. Yan, Q. H. Zhang, W. L. Gu, L. Gu, S. P. Beckman, L. Huang, C. Z. Zhu, *ACS Catal.* **2020**, *10*, 6422.

[4] D. Chao, Z. Yu, J. Chen, Q. Dong, W. Wu, Y. Fang, L. Liu, S. Dong, *Nano Res.* **2023**, *16*, 189.
